# Computationally Generated Plant-Derived Berberine-Based Hybrid Compounds as Potential Dual Binders to *Staphylococcus aureus* FtsZ/FabI Enzymes: A Ligand-Based Approach

**DOI:** 10.3390/ijms27157035

**Published:** 2026-08-05

**Authors:** Julio César Robles-Romero, Jael Quintero-Vargas, Karen Ochoa Lara, Mario Alberto Leyva-Peralta, Milagros Aguilar-Martínez, Luis Eduardo Hernandez-Dominguez, Francisco José Palacios-Can, Simon Bernard Iloki-Assagna, Rodrigo Said Razo-Hernández, Juan Carlos Gálvez-Ruiz

**Affiliations:** 1 Departamento de Ciencias Químico-Biológicas, Universidad de Sonora, Blvd. Luis Encinas y Rosales s/n, Hermosillo C.P. 83000, Sonora, Mexico; a213207118@unison.mx (J.C.R.-R.); simon.iloki@unison.mx (S.B.I.-A.); 2 Departamento de Ciencias de la Salud, Universidad de Sonora, Blvd. Bordo Nuevo s/n, Ciudad Obregón C.P. 85039, Sonora, Mexico; jael.quintero@unison.mx; 3 Departamento de Investigación en Polímeros y Materiales, Universidad de Sonora, Rosales y Encinas s/n, Hermosillo C.P. 83000, Sonora, Mexico; karen.ochoa@unison.mx; 4 Departamento de Ciencias Químico Biológicas y Agropecuarias, Universidad de Sonora, Avenida K s/n, Caborca C.P. 83600, Sonora, Mexico; mario.leyva@unison.mx (M.A.L.-P.); milagros.aguilar@unison.mx (M.A.-M.); 5 Laboratorio de Quimioinformática y Diseño de Fármacos, Centro de Investigación en Dinámica Celular (CIDC), Instituto de Investigación en Ciencias Básicas y Aplicadas (IICBA), Universidad Autónoma del Estado de Morelos, Av. Universidad No. 1001, Cuernavaca C.P. 62209, Morelos, Mexico; luis.hernandezdom@uaem.edu.mx (L.E.H.-D.); fjpc@uaem.mx (F.J.P.-C.)

**Keywords:** berberine, FtsZ, FabI, hybrid compounds, multitarget approach, lipophilic acids

## Abstract

*Staphylococcus aureus* (*S. aureus*) remains a major global pathogen and a significant public health concern due to its antibiotic resistance. This has spurred the search for new treatments, resulting in the discovery of two promising targets: FtsZ and FabI. Naturally occurring compounds berberine and lipophilic acids are known to bind these enzymes, respectively. This study aims to improve berberine’s binding affinity for FtsZ and enhance its interaction with FabI by designing hybrid compounds that could serve as dual inhibitors, targeting both active and allosteric sites. Forty-eight hybrids, derived from berberine and lipophilic acids with 10 to 22 carbons, were modeled. Molecular docking against five *S. aureus* enzyme crystal structures identified six compounds with geranic acid chains (**1s**, **1t**, **1u**, **2s**, **2t**, **2u**) that showed the strongest binding. Among these, **2s**, **2t**, and **1t** showed the greatest affinity for FtsZ, while **2u**, **1u**, and **1s** targeted FabI, with binding energies around −8.2 to −10.5 kcal/mol. QSAR models estimated MICs within known inhibitor ranges, implying potential effectiveness. Hydrophobic and flexible features correlated with stronger interactions and activity. ADMET analysis indicated low toxicity for these hybrids. Modifying berberine with lipophilic acids appears to be a promising approach for developing plant-based dual inhibitors against *S. aureus*.

## 1. Introduction

One of the most significant challenges in public health is the increasing prevalence of antibiotic-resistant bacteria, accompanied by the concurrent expansion of their antibiotic-resistance repertoires. The World Health Organization (WHO) has reported that various bacteria, such as *E. coli* and *S. aureus*, have developed resistance to commonly used antibiotics, including methicillin, as well as to last-resort antibiotics, such as fluoroquinolones [1].

Specifically, *S. aureus* is one of the most important bacteria in the ESKAPE classification, a list of multidrug-resistant pathogens of critical priority. Although *S. aureus* is a commensal Gram-positive bacterium that is part of our microbiota, mainly on the skin, various pathogenic strains have posed a significant challenge in healthcare due to their remarkable adaptability to environmental stressors, which can be summarized into two main categories: (1) external agents and (2) nutrient scarcity [2,3]. *S. aureus* can develop resistance to many commonly used and last-resort antibiotics, including various beta-lactams (like methicillin), glycopeptides, fluoroquinolones, aminoglycosides, tetracyclines, and macrolides. Part of its resistance mechanisms involves modifying target sites where common antibiotics bind. For example, it can change DNA gyrase, the target of fluoroquinolones [3]. Additionally, resistance to multiple bacteriophages has been reported, compromising even this alternative therapy. On the other hand, under stress conditions such as iron scarcity, *S. aureus* employs various strategies, including the production of staphyloferrin, which influences the presence of commensal nasal bacteria [3].

Therefore, the search for new strategies to enable the rational design of compounds with antibacterial activity against multidrug-resistant (MDR) bacteria, especially *S. aureus*, is a worldwide goal. Plants have long served as sources of traditional remedies in ancient medicinal practices. Some phytochemicals isolated from these remedies have been studied for potent and/or selective biological activities that could be used to treat diseases. The broad biological activity and chemical diversity of natural products have led to the discovery of new drugs. It is known that more than 41.9% of the approved drugs in the last 40 years have been derived from natural resources. If synthetic molecules with a natural-product pharmacophore or a natural-product mimic are included, the percentage increases to 56% [4]. Clinically important anticancer agents, such as paclitaxel, camptothecin, and vinblastine, as well as many other promising compounds, are plant-derived [5]. Currently, approximately 58% of antibacterial drugs are derived from natural products [4]. More recently, computational approaches to address the MDR problem have gained traction in the discovery of new antibacterial entities [6,7]. Alongside this, considering specific chemical properties, such as hydrophobicity, could be a practical approach for semi-synthesizing new compounds or repurposing existing ones. One example is ibuprofen, an anti-inflammatory drug that has recently been shown to exhibit antibacterial activity against *S. aureus*, primarily by disrupting the bacterial cell wall [8,9]. Another approach to tackling this challenge is to explore new biological targets, such as FtsZ and FabI. Inhibiting these proteins directly leads to bacterial cell death [10,11].

**Figure 1:**
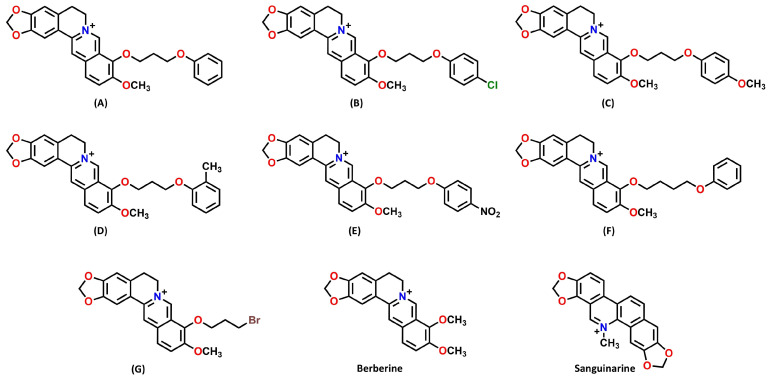
2D representations of berberine and its 9-phenoxyalkyl-substituted derivatives synthesized by Sun et al. [11]. (**A**) Berberine derivative 1, (**B**) berberine derivative 2, (**C**) berberine derivative 3, (**D**) berberine derivative 4, (**E**) Berberine derivative 5, (**F**) Berberine derivative 6, and (**G**) berberine derivative 7. Bromine, chlorine, oxygen, and nitrogen are shown in brown, green, red, and blue, respectively.

The filamentous temperature-sensitive mutant Z (FtsZ) enzyme is responsible for bacterial cell division, specifically binary fission (secondary structure of FtsZ is shown in Figure S1). It works by polymerizing to form the so-called “Z-ring” at the center of the bacterial cell. During constriction, once the bacterium has duplicated its contents, the ring facilitates the partitioning of the cell into two daughter cells. This enzyme comprises two binding sites separated by a central H7 helix at its core: the catalytic site, where GDP binds, and the allosteric site [8]. Research has shown that various inhibitors, such as 9PC, 3-[(6-chloro [1,3]thiazolo [5,4-b]pyridin-2-yl)methoxy]-2,6-difluorobenzamide, bind to the allosteric site, which is primarily hydrophobic [11]. In some cases, alkaloids such as berberine have demonstrated affinity for this enzyme, where structural modifications of these compounds have been shown to improve both their binding affinity for FtsZ and their antibacterial activity against *S. aureus* [12,13] (Figure 1; 3D representation in Figure S2).

**Figure 2:**
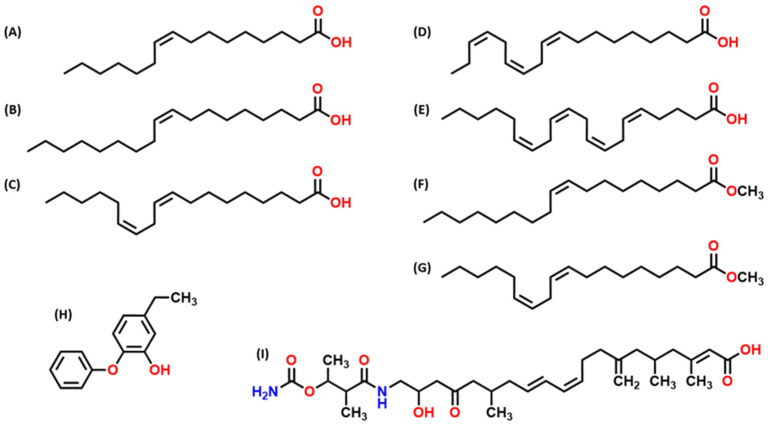
Reported FabI inhibitors (**A**), palmitoleic acid; (**B**), oleic acid; (**C**), linoleic acid; (**D**), α-linolenic acid; (**E**), arachidonic acid; (**F**), methyloleate; (**G**), methyllinoleate; (**H**), 5-ethyl-2-phenoxyphenol (E9P); (**I**), kalimantacin A. Oxygen and nitrogen are shown in red and blue, respectively.

The enoyl-acyl carrier protein reductase (FabI) enzyme, whose secondary structure is shown in Figure S3, elongates endogenous fatty acids, thereby supporting the second stage of fatty acid synthesis for incorporation into the bacterial cell wall. FabI’s catalytic action is mediated by the NADP cofactor. It has been shown that compounds that effectively inhibit this enzyme bind directly to NADP, an essential consideration in the design of FabI-targeting drugs. Inhibiting FabI disrupts cell wall biosynthesis, ultimately leading to bacterial death [13,14]. Kalimantacin A is a potent FabI inhibitor because of its structural mimicry of phosphatidylglycerol, the enzyme’s substrate [15]. In addition, the FabI enzyme is a primary fatty acid-binding enzyme that catalyzes the final step in fatty acid elongation, a process required for the synthesis of bacterial cell wall phospholipids. Although the phospholipid composition varies among bacterial species, the final fatty acid chain length typically remains conserved at 12–14 carbon atoms. Studies have identified palmitoleic and linoleic acids as the highest-affinity natural substrates, with optimal activity observed for 16–18-carbon chains containing ≤2 double bonds (Figure 2; 3D representation in Figure S4) [16]. Adding lipophilic fragments is already a well-established strategy for enhancing biological activity. Specifically, linear lipophilic chains of no more than 12 carbon atoms have been shown to increase the antiproliferative activity of asperigimycins more than cyclic lipophilic fragments. This was reflected in a decrease in IC_50_ values, with antiproliferative activity improving from >10 to 0.072 µM [17].

Another strategy that has gained interest among the scientific community to address the MDR issue is multitarget drug design. Many diseases, including cancer and infectious diseases, have multifactorial origins and multiple response mechanisms, and the best way to treat them is to target multiple biological targets [18,19]. One strategy for designing dual-action drugs is to consider the pharmacophores of two distinct drugs and design a hybrid structure that combines them [20]. Such drugs are called hybrid drugs [21], and they have shown great success in biological evaluations for cancer and antibacterial applications [21,22,23]. Compounds, mainly flavonoids, terpenes, and alkaloids, have been structurally modified, demonstrating increased biological activities, including antibacterial, antifungal, antioxidant, and antiproliferative activities. In particular, alkaloids, such as berberine, are a broad group of structurally diverse plant metabolites with important biological activities, making them an attractive pharmacophore whose pharmacological properties can be modulated by chemical modification [24]. In this regard, berberine hybrid compounds, in combination with other drugs such as chloramphenicol, have a strong record of antibacterial activity against Gram-negative and Gram-positive bacteria, particularly *S. aureus* [25,26,27]. Berberine has also been reported to exhibit anti-inflammatory activity and beneficial effects against metabolic disorders (such as diabetes) and cardiovascular diseases [24].

It is known that ester formation increases the lipophilicity of the parent compounds. However, when aiming to preserve the integrity of the ester in medicines, two main issues arise: (1) the pH of the gastrointestinal tract, and (2) carboxylesterase enzymes (CE) [28]. Specifically, humans possess CE1 and CE2 enzymes. CE1 is mainly expressed in the liver and lungs and hydrolyzes esters bearing small alkoxy groups. In contrast, CE2 is primarily expressed in the small intestine and kidneys and hydrolyzes esters bearing small acyl groups. This has posed a challenge for various prodrugs; for instance, ketoprofen derivatives have exhibited low bioavailability due to hydrolysis by the aforementioned enzymes [29].

On the other hand, diesters have been shown to withstand pH 1.2. Furthermore, their degradation upon exposure to CE1 and CE2 reaches 50% after 6 h, regardless of whether enzymes synthesized by lung or intestinal cells are used, which could be regarded as their plasma half-life [29,30]. These reported results, together with those for ibuprofen and paracetamol, whose half-life ranges from 1.8 to 3 h [31], show that diesters maintain their integrity for a considerable time, even in the presence of esterase enzymes. Therefore, the use of compounds bearing this functional group is a viable strategy [24].

This ester synthesis strategy has previously enabled in vitro evaluations of several berberine derivatives bearing two carboxylic acid chains attached at positions 2 and 3 of berberine, namely geranic acid and sorbic acid. These two lipophilic acids share a common structural motif characterized by two double bonds and a short-chain backbone of no more than ten carbon atoms. While derivatives of both fatty acids exhibited antibacterial activity, the compound bearing two geranic acid moieties showed greater activity [32].

Ester functional groups are often favored over other bioisosteres because their carbonyl groups are more likely to form polar electrostatic interactions with important active-site residues. This can facilitate noncovalent interactions that enhance both affinity and selectivity [33,34], aiding drug development.

In this study, we explore the potential inhibition of FtsZ and FabI from *S. aureus* by novel berberine-hybrid compounds using in silico ligand-based drug design. We combined a plant-derived pharmacophore, the berberine alkaloid [35], with lipophilic acids (geranic, myristic, palmitic, oleic, arachidonic, palmitoleic, stearic, and linoleic acid) to leverage their inhibitory activity against FtsZ [36] for berberine and against FabI for the fatty acids. Esterification of berberine with lipophilic acids could yield hybrid compounds with superior antibacterial activity compared to berberine alone, due to increased hydrophobicity and a better fit within hydrophobic pockets of bacterial targets such as FtsZ or FabI. It has been demonstrated that inhibiting these proteins directly leads to bacterial cell death [10,11]. Berberine and several derivatives obtained by adding fragments at the 8 O-methyl position have been evaluated against FtsZ. However, to date, no in silico experiments have been performed to evaluate their affinity for FabI, nor have studies been conducted to determine the in silico affinity of berberine derivatives bearing two lipophilic acid chains. Therefore, strategically placing esters at key points on a scaffold enhances lipophilicity, affinity, and molecular interactions, ultimately improving drug candidate profiles. Furthermore, the synthesis of similar berberine hybrid compounds has been reported [23], as have other berberine derivatives with structural modifications at the sites of interest [13], supporting our in silico design. Affinity for the biological targets was evaluated by computational molecular docking using different FtsZ and FabI conformations. Finally, in silico ADMET analysis was performed for the best candidates. The results provide a new perspective on how adding one or two lipophilic fragments to either side of the berberine scaffold affects binding to and affinity for FtsZ and FabI, two key bacterial targets.

## 2. Results

### 2.1. Ligand-Based Drug Design

For this study, we designed hybrid compounds (Table 1) by combining two sets of biologically active compounds against *S. aureus* obtained from plant sources: alkaloids and lipophilic acids. For the alkaloid derivatives, we used two scaffolds: berberine (BBR) and 2,3-dihydroxyprotoberberine (2,3-DHP), which are reported as inhibitors of FtsZ [12,13]. For the lipophilic acids, we used eight, including saturated fatty acids (stearic, palmitic, and myristic acids), monounsaturated fatty acids (palmitoleic and oleic acids), and polyunsaturated fatty acids (geranic, linoleic, and arachidonic acids), which have been reported as biologically active against FabI [1].

We modified the berberine and 2,3-dihydroxyprotoberberine scaffolds based on previous work supporting modification of BBR at one of its -OCH_3_ groups, yielding many ether derivatives [13], and the work reported by our group where 2,3-DHP was modified by adding different lipophilic acids over the two -OH after opening the acetal ring, yielding different ester derivatives [23]. Therefore, combining all this information, we designed 48 hybrid berberine-fatty acid derivatives in silico, divided into two sets of 24 compounds. The first set (**1a-x**) uses 2,3-DHP as the scaffold and considers dual esterification of the -OH and individual substitution of each -OH. The second set of compounds (**2a-x**) uses the BBR scaffold and considers the esterification strategy for each individual and dual -OCH_3_ substitution (Table 1).

As shown in Table 1, the structural complexity of these molecules is strongly related to the flexibility and number of carbon atoms of the added lipophilic acid. Therefore, a conformational analysis was performed before docking calculations to obtain more reliable structures for each compound (see Materials and Methods). From these calculations, three conformers were selected based on their ovality: one with the highest (cylinder-type), one with the medium, and one with the lowest (sphere-type) values; see Figures S5 and S6 and Table S1. The major structural changes in these hybrid compounds occur in the alkyl chain, leaving the alkaloid structure unaltered (Table S1).

We used these conformers of each hybrid compound as a seed for molecular docking to obtain the best binding mode (interaction energy) of these compounds.

### 2.2. FtsZ and FabI Molecular Docking Protocol Validation

#### 2.2.1. Binding Mode and Energy Validation Protocol for FtsZ

**Figure 3:**
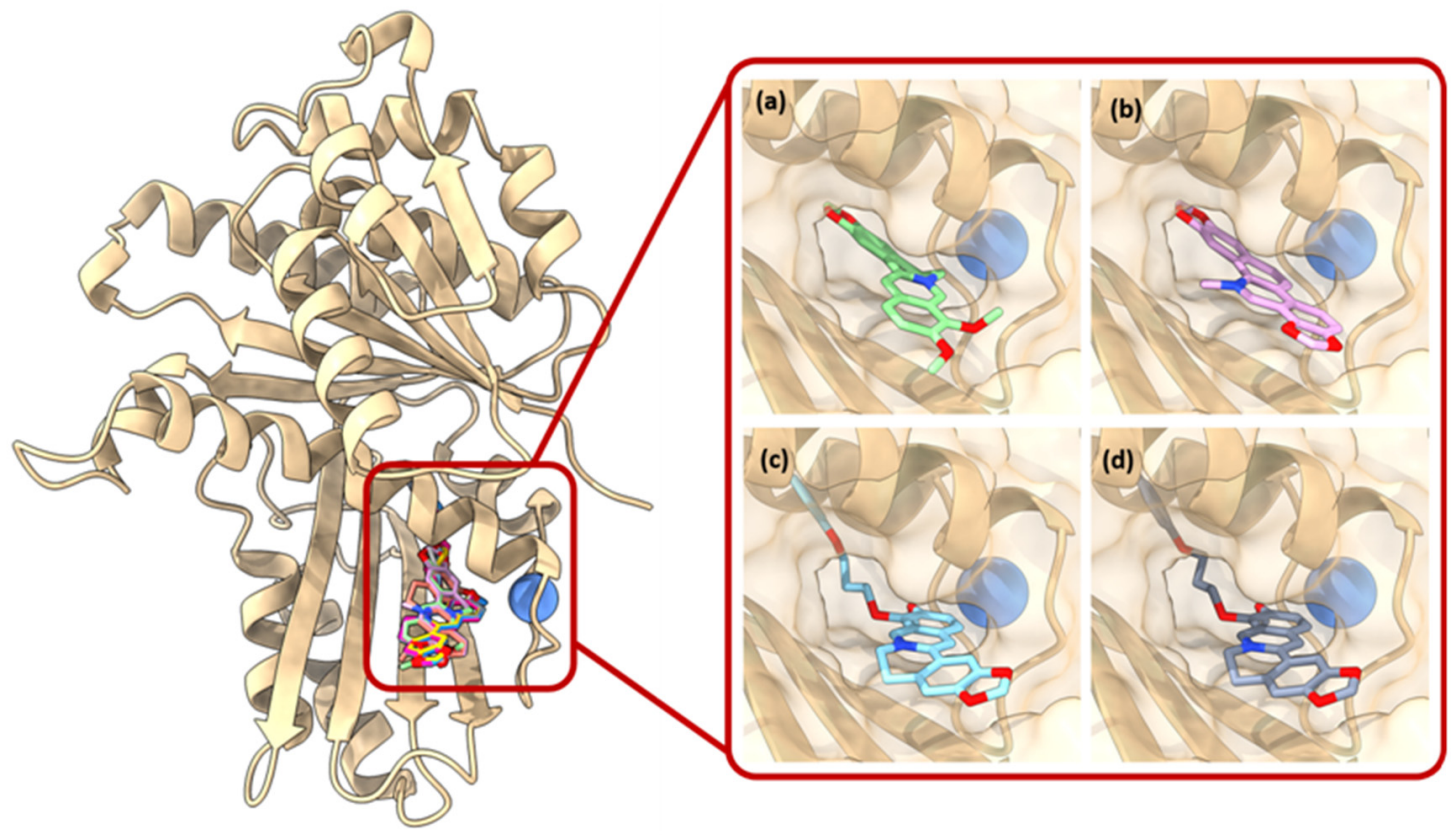
(**left**) Side view of the FtsZ protein (**5xdt**) with the predicted binding modes of selected berberine-geranic acid derivatives. (**right**) Expanded regions of the docked compounds: (**a**) berberine, (**b**) sanguinarine, (**c**) and (**d**) 9-phenoxyalkyl-substituted derivatives into the hydrophobic site of FtsZ. The calcium ion is depicted as a light-blue solid sphere.

To obtain reference data for biologically active compounds with reported antibacterial activity against FtsZ of *S. aureus* [13], we conducted molecular docking of berberine derivatives synthesized by Sun et al. [13], as well as berberine and sanguinarine, to obtain their binding modes with FtsZ. One of the most distinctive structural features of FtsZ is its secondary structure, the α-helix 7 (H7), located in the central domain (Figure 3), which separates the terminal domains of FtsZ [12] and contains the allosteric pocket. In Figure 3, the binding modes of the hybrid compounds with higher interaction energy, as well as berberine and sanguinarine, are displayed.

The binding modes of all the berberine compounds were governed by their hydrophobic properties. For berberine and sanguinarine, the binding mode involves inserting the acetal ring into the hydrophobic subpocket of the allosteric site, with the methoxy groups oriented toward the calcium ion. In contrast, for the derivatives synthesized by Sun et al. [13], the binding mode was opposite: the acetal ring was oriented toward the calcium ion and exposed to the solvent. Meanwhile, the substituted ring bearing the methoxy groups was oriented toward the hydrophobic subpocket, as the added molecular fragment is hydrophobic.

**Figure 4:**
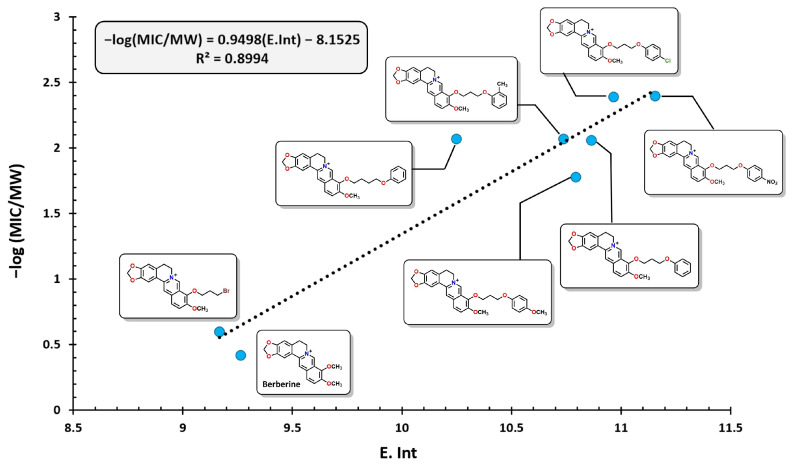
Linear relationship between −*Log*Y and the interaction energy (*E_int_*) of FtsZ from *S. aureus* and berberine derivatives; Y denotes the ratio of MIC to MW. The dotted line shows the ideal fit.

Additionally, to assess the predictive power of our docking protocol, we examined the relationship between the obtained interaction energy (*E_int_*) and the experimental minimum inhibitory concentration (MIC) for FtsZ using a QSAR approach. In Figure 4, the linear relationships between the interaction energy (*E_int_*) obtained from molecular docking and the MIC ratio and the molecular weight (MW) are shown. To display the potency of the compounds in a more interpretable way, the logarithmic transformation was applied to the MIC and *MW* ratio (−*Log*(MIC/MW)), and *E_int_* was multiplied by −1 [26].

From QSAR Equation (1), we can see that increasing the interaction energy (***E_int_***) obtained from molecular docking yields compounds with lower MIC values; the model’s statistical parameters support this.

(1) $$-Log\left(\frac{MIC}{MW}\right)=0.9498E_{inter}+-8.1525$$

R^2^ = 89.94 Q^2^ = 84.04 s = 0.262 F = 53.65 SDEP = 0.2901

The QSAR model is significant because it establishes a quantitative relationship between *E_int_* and −*Log*(MIC/MW) for berberine derivatives and provides structural insights into how *E_int_* changes with structural modifications. This model is limited to berberine derivatives targeting FtsZ in *S. aureus* and will be used to select hybrid compounds designed in this study.

As shown in Figure 4, increasing the alkyl chain length and hydrophobicity of the substituent bound to berberine results in a higher *E_int_*, which correlates with the antibacterial activity (lower MIC values). The substituents in the *para* position on the phenyl ring are preferred, as this molecular shape helps the molecules reach the hydrophobic subpocket of FtsZ (Figure 3). As mentioned, chemical complementarity is crucial for binding; in this case, the electronic (σ-Hammett constant) and hydrophobic (π, Hansch constant) nature of the substituents on the phenyl ring. Additionally, the steric effect is negative for *ortho* substitutions. It favors the *meta* and, to a major extent, the *para* positions for these berberine derivatives, which is directly related to the shape complementarity of the allosteric binding site of FtsZ and its hidden hydrophobic subpocket.

#### 2.2.2. Binding Mode and Energy Validation Protocol for FabI

**Figure 5:**
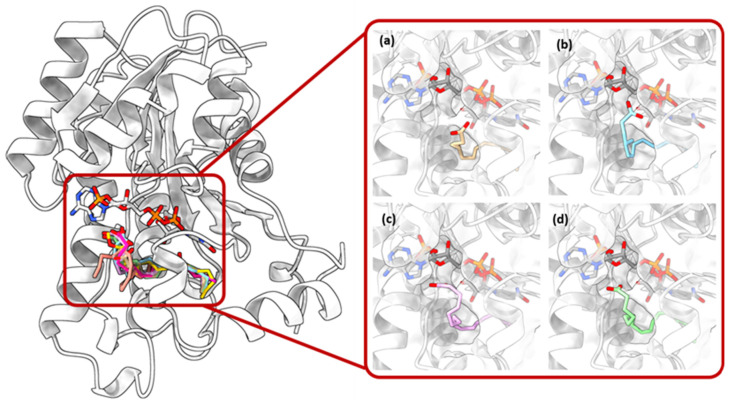
(**left**) Side view of the FabI protein (**4alk**) with the predicted binding modes of selected lipophilic acids. (**right**) Expanded regions of the docked compounds: (**a**) palmitoleic acid, (**b**) oleic acid, (**c**) linoleic acid-18:2, (**d**) α-linolenic acid-18:3, into the hydrophobic site of FabI. The NAD cofactor is also represented as colored sticks.

For the FtsZ system, structural binding modes and interaction energy values for biologically active compounds targeting *S. aureus* FabI were required as references. Therefore, we used the fatty acids reported by Zheng et al. [14] and the set of compounds reported as FabI inhibitors [37]. In Figure 5, the binding modes of four lipophilic acids: palmitoleic acid (A), oleic acid (B), linoleic acid-18:2 (C), and α-linolenic acid-18:3, along with the remaining FabI inhibitors, are displayed.

**Figure 6:**
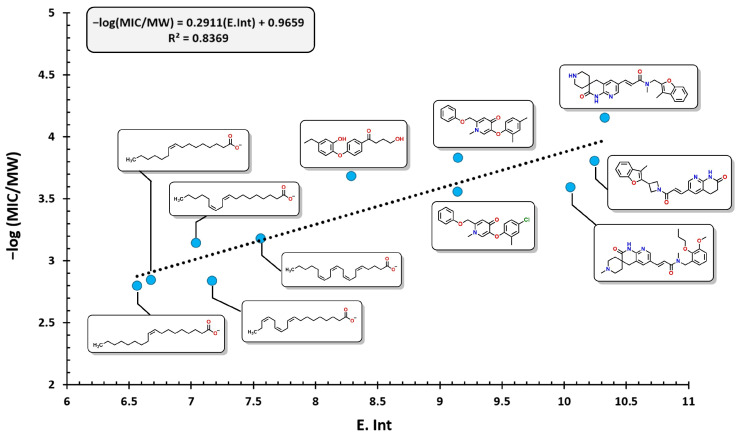
Linear relationship between −*Log*Y and the interaction energy (*E_int_*) of FabI of *S. aureus* and lipophilic acids; Y denotes the ratio of MIC to MW. The dotted line shows the ideal fit.

According to Figure 5, the lipophilic acids bind similarly, with the carboxylate motif located in the center of the catalytic pocket of FabI interacting with the oxygen atoms of the NAD phosphate groups. The alkyl chain binds in the hydrophobic region of the catalytic pocket, accommodating according to its size. As we analyzed the FtsZ system, we examined the relationship between the *E_int_* values obtained from molecular docking of lipophilic acids with FabI and their MIC values (Figure 6).

Regarding FtsZ, our FabI model indicates that strong binding to FabI (*E_int_*) enhances the compound’s antibacterial activity, as reflected in MIC values. This QSAR model (Equation (2)) can assist in optimizing the molecular structure of compounds, such as fatty acids, used as antibacterial agents against *S. aureus* by targeting FabI.

(2) $$-Log\left(\frac{MIC}{MW}\right)=02911E_{inter}+0.9659$$

R^2^ = 83.69 Q^2^ = 75.94 s = 0.198 F = 46.2 SDEP = 0.217

As shown in Figure 4 and Figure 6, a stronger correlation between the experimental data (−*Log* MIC/MW) and the *E_int_* was observed for the FtsZ system than for the FabI system. This may be related to the large number of compounds with biological data and to the compound type (more rigid), compared with the compounds used for FabI, which are more flexible. If a ligand is larger than the binding site, part of the molecule protrudes beyond the pocket and remains outside, even considering different protein conformations. Conversely, a smaller or similarly sized ligand can fit snugly into the binding site. However, even if the dimensions are appropriate for shape complementarity, the chemical properties of the binding site may preclude effective binding. Therefore, it is crucial to consider the chemical properties of amino acids, such as polarity and charge, to achieve chemical complementarity.

Figure 6 shows a correlation between the number and location of unsaturation in the alkyl chains of lipophilic acids, with arachidonic acid having four unsaturations positioned in the center of the alkyl chain, between carbon atoms 4 and 16. This is important because it relates to the molecular shape complementarity of the FabI inhibitors; for example, for linoleic acids, greater potency is achieved with linoleic acid 18:2 than with α-linoleic acid 18:3. Therefore, a rigid and aromatic scaffold, with flexible fragments attached, is necessary for greater inhibition of FabI, like our berberine derivatives.

Additionally, the structural validation of the molecular docking protocol for the entire FtsZ and FabI systems was performed (see Materials and Methods) using the Root Mean Square Deviation (RMSD) as a metric; an RMSD of less than 2.0 Å is generally considered acceptable for system validation [38]. For the **6kvq** and **5xdt** crystals, the RMSD values of the enzyme-inhibitor system were 0.9543 Å and 0.2056 Å, respectively. For the FabI system, the RMSD values for the **4alk** and **6tbb** crystals were 0.1222 Å and 0.9543 Å, respectively. These values show that across all systems, the RMSD profiles do not deviate significantly, confirming the reliability of the docking protocol and supporting the prediction of binding modes for our berberine and lipophilic acid-hybrid compounds in this computational study.

After parametrizing and validating our molecular docking methodology for the two biological systems of interest, we evaluated all designed berberine derivatives to identify the candidates that interact more strongly with the FtsZ and FabI enzymes from *S. aureus*.

### 2.3. Molecular Docking of Berberine-Hybrid Compounds with FtsZ

Various conformations that the FtsZ enzyme naturally adopts during catalysis have recently been described [39,40]. These conformational changes influence the presence, size, and chemical properties of cavities, depending on the specific amino acids involved. Understanding these features and the enzyme’s druggability is essential for determining whether a compound can bind to its active site. Since the hybrid compounds proposed herein are primarily nonpolar, the number of nonpolar amino acids in the cavities is substantial. Conformational changes in the FtsZ enzyme can create new cavities or enlarge existing ones, thereby enhancing its potential to bind drugs.

Three different FtsZ crystals were used to study the allosteric pocket most robustly [39]: one in the apo form (**3voa**) and the other two in complexes with fluoxapiprolin and 2,6-bis(fluoranyl)-3-[[6-(trifluoromethyl)-[1,3]thiazolo[5,4-b]pyridin-2-yl]methoxy]benza-mide (TXA707) as inhibitors, in the **6kvq** and **5xdt** crystals, respectively. The pockets were analyzed using the DoGSiteScorer tool from the Protein Plus platform. The molecular shapes of each allosteric pocket, as determined from the FtsZ crystal structure, are shown. Additionally, the structural and physicochemical properties of each pocket are shown (Table 2).

As shown, the allosteric pocket of each FtsZ crystal varies with its molecular shape and structural parameters, such as volume or hydrophobicity ratio. Nevertheless, based on the drug score, the allosteric pocket is likely to accommodate drug-like molecules. Therefore, blind molecular docking experiments were conducted to assess the affinity of berberine and the hybrid compounds for the FtsZ enzyme. These experiments aimed to identify the specific regions of the enzyme where these compounds are most likely to bind (see Materials and Methods, Section 4.3). The best candidates were selected based on their highest interaction energy (*E_int_*) to each crystal, with the selection criterion requiring consistency across all crystals.

Although the molecular docking was conducted on three crystallographic forms of FtsZ, including one in the apo form (**3voa**) and the other two in complex with an allosteric inhibitor (**6kvq** and **5xdt**), we present and discuss results for the **5xdt** crystal form because the allosteric inhibitor is similar in size to berberine. Table 3 presents the subpocket analysis of the **5xdt** crystal form, while the corresponding analyses for **3voa** and **6kvq** are shown in Tables S2 and S3, respectively. The complete molecular docking analyses for **3voa** and **6kvq** are shown in Figures S6–S9.

Analysis of the subpockets is necessary to understand the binding modes of the berberine candidates. In this crystal, subpocket1 has the largest ligand-binding volume among all analyzed FtsZ structures (V = 672.83 Å^3^) and the highest druggability score (0.79). Meanwhile, subpocket 2 has a volume of V = 301.82 Å^3^ and a druggability score of 0.54. The parameters used to conduct the analysis are described in the Materials and Methods Section.

This subpocket comprises 41 amino acid residues. Notably, it contains a high proportion of valine (Val), an aliphatic hydrophobic amino acid that confers flexibility to this site and is primarily responsible for its hydrophobic character. Previous molecular docking studies using the crystal structures **6kvq** and **5xdt** [36,39] have already identified conformational changes within the β-sheet of this enzyme, which is located in the two subpockets discussed here.

In contrast, subpocket 2 consists of only 11 amino acid residues; seven correspond to isoleucine (Ile), leucine (Leu), and valine (Val). Given that these residues are known to exhibit conformational flexibility and are the predominant components of this binding site, as in subpocket 1, this composition may facilitate conformational rearrangements that enable better ligand accommodation within the binding pocket.

**Figure 7:**
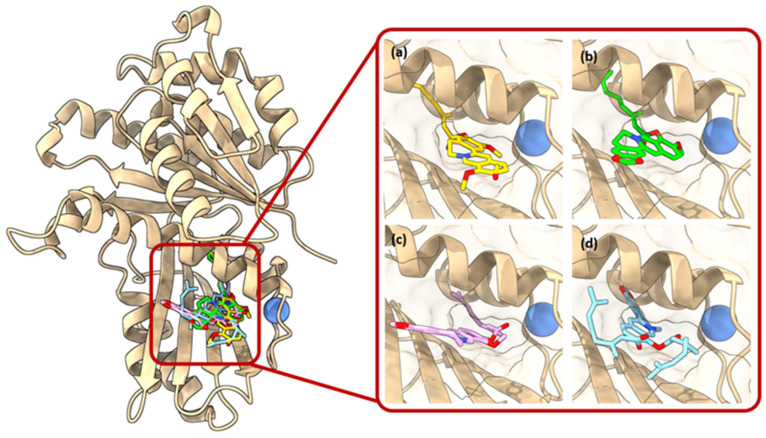
(**left**) Side view of the FtsZ protein (PDB:**5xdt**) with the predicted binding modes of selected berberine-geranic acid derivatives. (**right**) Expanded regions of the docked compounds (**a**) **1t**, (**b**) **2s**, (**c**) **2t**, and (**d**) **2u** into the hydrophobic site of FtsZ. The calcium ion is depicted as a light-blue solid sphere.

In Figure 7, the binding of the candidates with the highest interaction energy with FtsZ is displayed. It is observed that molecules **1t**, **2s**, and **2t** have their alkyl fragments within the hydrophobic subpocket, whereas the alkyl fragments of **2u**, the lipophilic acid, are oriented towards the solvent. The hybrid compounds **1t** and **2s** have the highest calculated interaction energies, *E_int_* = −10.57 and −10.59 kcal/mol, respectively. For **2t** and **2u**, high calculated interaction energies were also observed: *E_int_* = −10.44 and *E_int_* = −10.05 kcal/mol, respectively. The binding of **2t** is similar to that displayed by **1t** and **2s**, in which the geranic acid fragment is within the hydrophobic subpocket of FtsZ, and the 1,3-benzodioxole is exposed to the solvent. Contrary to **2t**, **2u** binds by inserting the 1,3-benzodioxole fragment into the lipophilic subpocket of FtsZ, due to the substantial increase in volume induced by the two geranic acid fragments added.

In comparison, the inhibitor TXA707 exhibited an *E_int_* value of −11.17 kcal/mol, which is close to the *E_int_* values of compounds **1t** and **1u**. Notably, TXA707 contains four fluorine atoms, which confer predominantly hydrophobic characteristics that facilitate binding to the FtsZ enzyme [13]. The closeness of the interaction energy between the hybrid compounds **1t** and **1u** and the reference inhibitor suggests that these berberine-derived compounds effectively exploit the available hydrophobic interactions within the binding site via their hydrophobic moieties.

**Figure 8:**
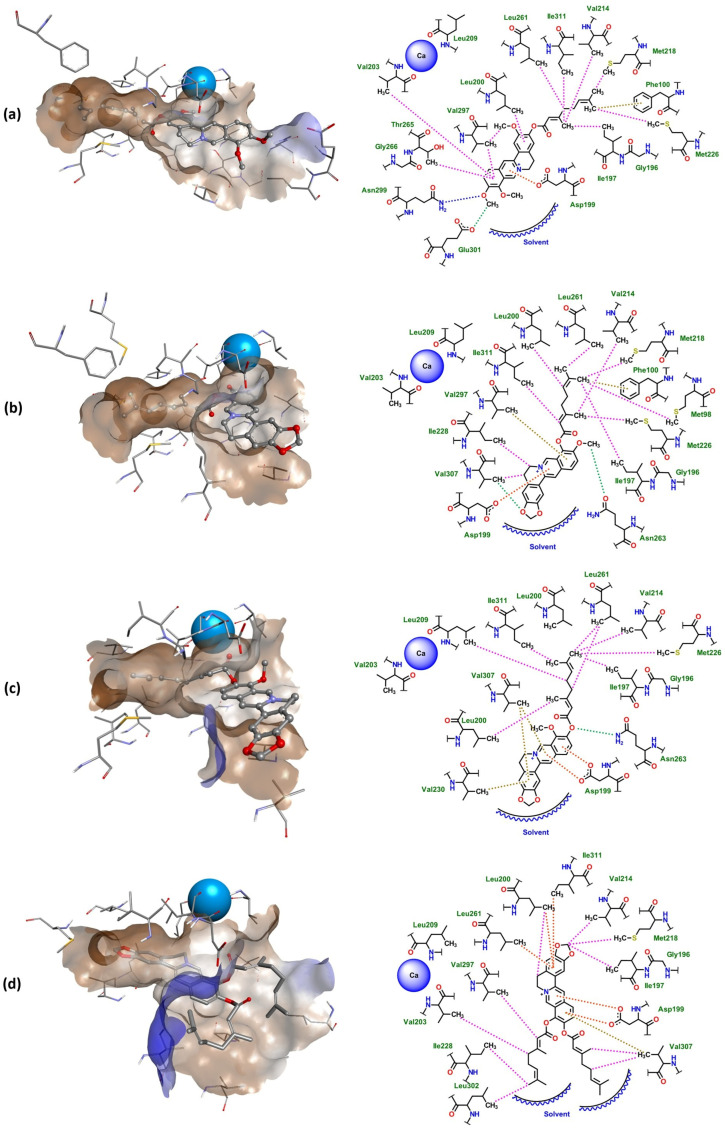
Hydrophobic surface representation of the FtsZ binding pocket (PDB:**5xdt**) in complex with compounds (**a**) **1t**, (**b**) **2s**, (**c**) **2t**, and (**d**) **2u**. The hydrophobic sites are depicted in brown/white, while hydrophilic regions are shown in blue. Hydrophobic interactions are represented in pink; π-CH interactions in orange; and hydrogen bonds in greenish dotted lines.

Figure 8 displays the hydrophobic interaction profile on FtsZ, depicted as its hydrophobic surface, along with the non-covalent interactions involving FtsZ residues.

A larger number of hydrophobic contacts, along with shorter distances between hydrophobic species, will increase the interaction energy. It is worth noting that Met98 and Phe100 interact with the methyl groups of the geranic acid alkyl chain in **2s**, forming alkyl-π interactions, which may explain the increase in the *E_int_* value of this compound. Meanwhile, for **1t**, the explanation for the increase in its *E_int_* is related to the different binding mode displayed, increasing the hydrophobic interactions with Met98, Phe100, Met226, Met218, Ile311, Leu261, Ile197, and Leu200 by inserting the geranic acid fragment into the lipophilic subpocket, which increases in almost 3 kcal/mol the *E_int_*. In the case of **2t** and **2u**, they bind similarly to **6kvq**. Nevertheless, an increase in their *E_int_* of ≈2 kcal/mol per candidate was observed. The flatter shape of subpocket1 in **5xdt**, compared to that of the subpocket in **6kvq**, facilitates alkyl-π interactions between the rings of the berberine scaffold and Val230 and Val307. We associate this molecular shape with the more cylindrical form of the cocrystallized ligand, which lacks the BODIPY fragment, thereby exposing other residues, such as Asn263, that are responsible for the subpocket’s flatness. Additionally, in **2t**, another interesting interaction occurs between the 1,3-benzodioxole fragment and Gln192, mediated by strong non-classical hydrogen bonds. Finally, for **2u**, the increase in *E_int_* can be explained by increased alkyl-π interactions, as previously mentioned, as well as by interactions between the 1,3-benzodioxole fragment and Leu200, Leu261, Ile197, and Ile311.

Consistent with the findings for the **5xdt** crystalline form, **3voa** and **6kvq** reproduced the same six best candidates, **1s**, **1t**, **1u**, **2s**, **2t**, and **2u**, based on their *E_int_* values and binding modes at the FtsZ allosteric site (Figures S7–S10).

### 2.4. Molecular Docking of Berberine-Hybrid Compounds with FabI

The same binding-site analysis used for FtsZ was applied to FabI, using the **4alk** and **6tbb** crystal structures (Table 5); both crystals are inhibitor-FabI complexes. In **6tbb**, FabI is bound to kalymantacine (a large compound); the binding site volume is 1791.36 Å^3^, maintaining hydrophobic properties that enhance the binding of drug-like compounds (hydrophobic ratio = 0.4). In the **4alk** crystal, FabI is bound to 5-ethyl-2-phenoxyphenol (E9P), a small compound, and the volume (V = 1352.64 Å^3^) is larger than the hydrophobic ratio (Hyd_ratio_ = 0.45). Both conformations contain the NADP cofactor. Using these crystal structures, we performed molecular docking of berberine and its hybrid compounds with FabI to assess their binding affinities. The conformations are optimal for binding drug-like compounds (drug score = 0.81), as calculated by the DoGSiteScorer tool on the Protein Plus platform (Table 4). Notably, NADP remains in both complexes.

**Table 4:** Structural and Physicochemical Characteristics of the Catalytic Site of FabI.

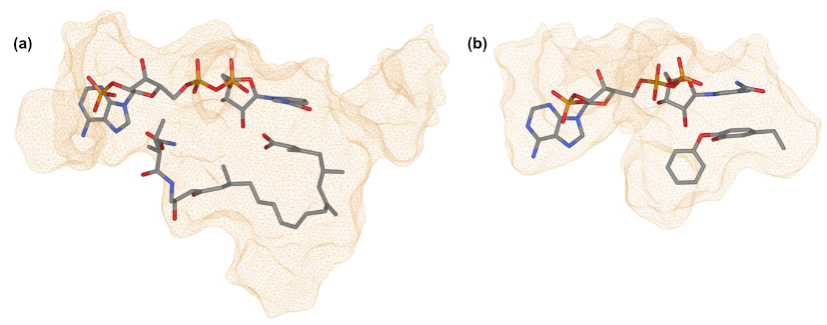							
Crystal Structure	Volume [Å^3^]	Surface [Å^2^]	Depth [Å]	Hydrophobicity Ratio	aPolar Ratio	Polar Ratio	Drug Score
**(a) 6tbb**	1791.36	1940.47	26.42	0.40	0.44	0.38	0.81
**(b) 4alk**	1352.64	1402.37	22.66	0.45	0.49	0.33	0.81

As can be appreciated, the cavities of the FabI have different molecular shapes; the inductive effect of the inhibitor was severe, as reflected in Figures (a) and (b) of Table 4. Even the cofactor changed its conformation. Therefore, these systems were studied separately to identify subpockets and analyze the physicochemical features of each, thereby better understanding the docking results. The most representative crystal structure to explore the inductive effect of an inhibitor at the FabI catalytic site is **6tbb**, which features kalimantacin, an inhibitor that mimics myristic acid behavior. In this FabI crystal form, five subpockets define the catalytic site: one of greater volume, where kalimantacin is located and therefore of greatest interest to our study (Table 5). In Table S4, all subpockets of the catalytic site in the (**4alk**) crystal are shown. The molecular docking poses of **4alk** are shown in Figures S11 and S12.

**Table 5:** Structural and Physicochemical Characteristics of the Binding Subpockets of the FabI Catalytic Site.

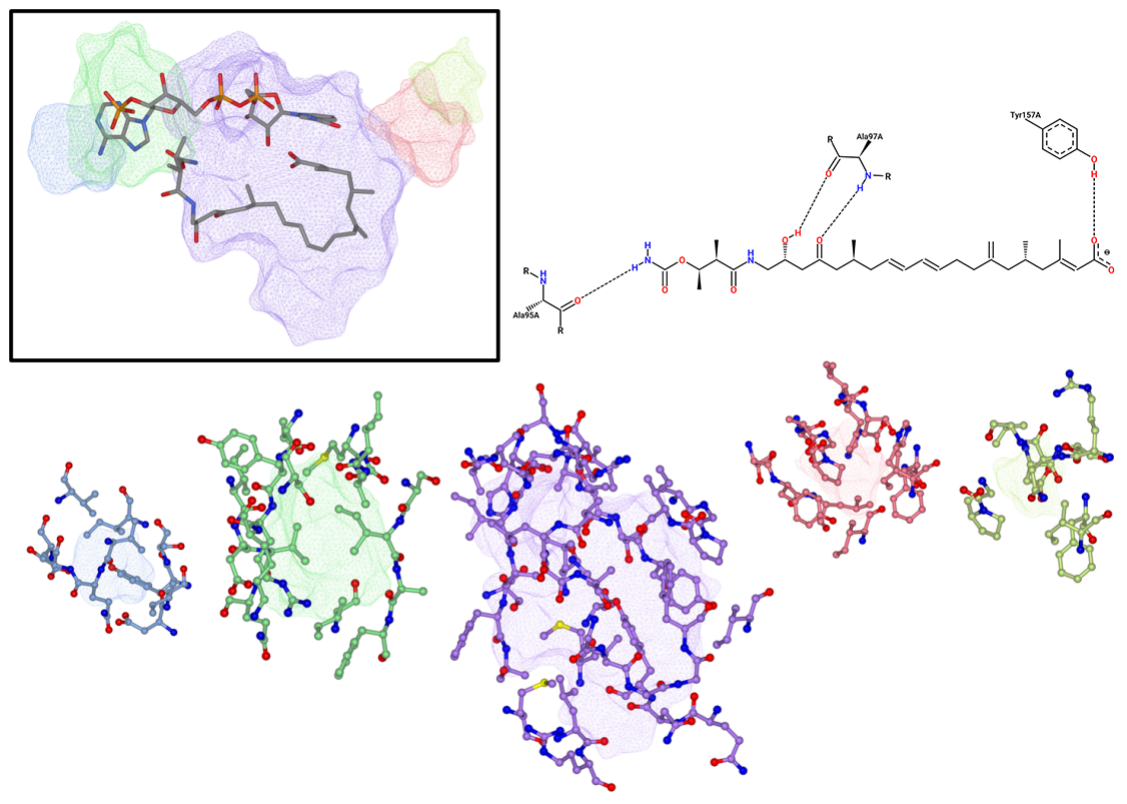							
PDB: 6tbb	Volume [Å^3^]	Surface [Å^2^]	Depth [Å]	Hydrophobicity Ratio	aPolar Ratio	Polar Ratio	Drug Score
Subpocket 1 (purple)	1211.07	1293.11	23.81	0.48	0.49	0.31	0.87
Subpocket 2 (green)	354.30	451.14	15.01	0.24	0.50	0.33	0.40
Subpocket 3 (red)	109.25	178.95	1.74	0.33	0.50	0.29	0.32
Subpocket 4 (blue)	72.06	201.63	0.69	0.13	0.40	0.40	0.30
Subpocket 5 (yellow)	44.67	92.89	0.89	0.12	0.44	0.33	0.19

Subpocket 1, where the kalimantacine binds, is the same region where E9P binds, but the binding of kalimantacine deforms the hydrophobic region in a greater way (V = 1211.07 Å^3^). In this case, the highly lipophilic region of subpocket 1 is composed of Ala95, Phe96, Ala97, Leu102, Tyr147, Val154, Tyr157, Met160, Pro192, Leu196, Ala198, Val201, Phe204, and Ile207. Additionally, its binding involves a hydrogen bond between the carboxylate group and the fructose ring of NADPH, as well as between the carboxylate group and Tyr157. All the candidates bind in subpocket 1 due to its larger size and hydrophobicity compared to the other subpockets.

**Figure 9:**
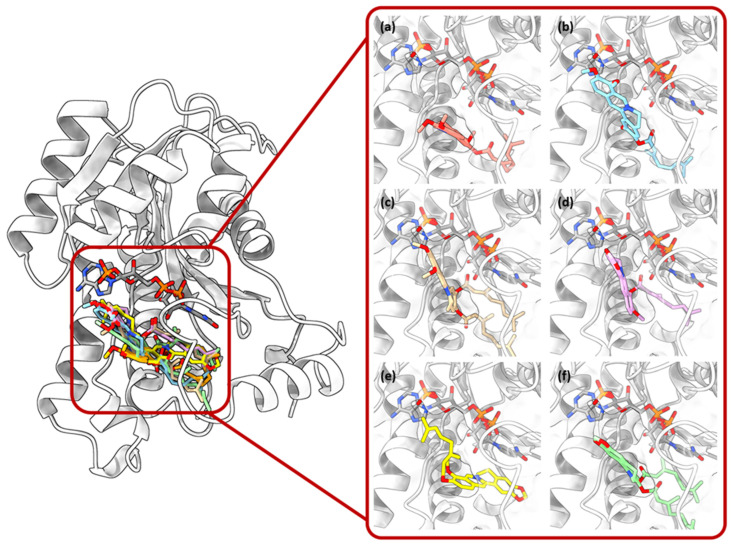
(**left**) Side view of the FabI protein (**6tbb**) with the predicted binding modes of selected berberine-fatty acid derivatives. (**right**) Expanded regions of the docked compounds (**a**) **1s**, (**b**) **1t**, (**c**) **1u**, (**d**) **2s**, (**e**) **2t**, and (**f**) **2u** into the hydrophobic site of FabI.

In Figure 9, the binding of **1s**, **1t**, **1u**, **2s**, **2t**, and **2u** within the catalytic site of the **6tbb** crystal is shown. All compounds insert the geranic acid fragment into the hydrophobic region, except **2t**, which inserts the berberine scaffold; even **1u** and **2u** insert their two geranic acid fragments into this pocket. Meanwhile, the berberine scaffold is exposed to the solvent area.

**Figure 10:**
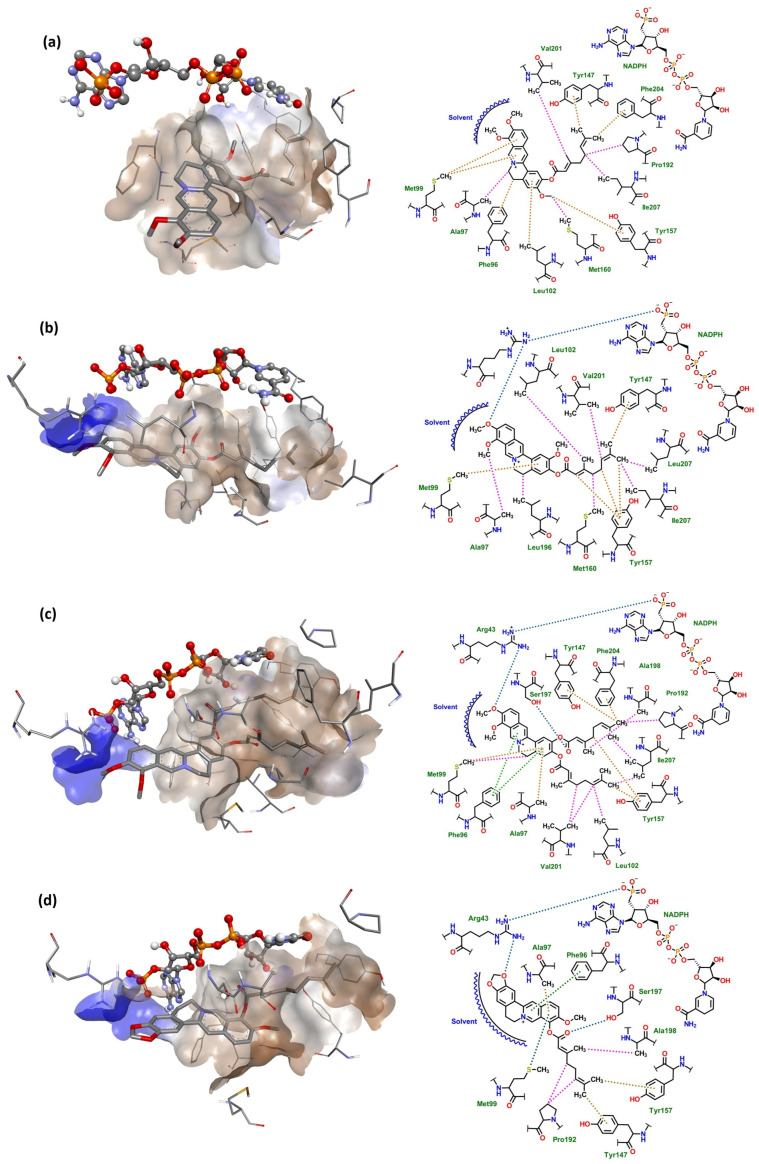
Hydrophobic surfaces and 2D representation of the FabI binding pocket (PDB: **6tbb**) in complex with compounds (**a**) **1s**, (**b**) **1t**, (**c**) **1u**, and (**d**) **2s**, and NADPH. The hydrophobic sites are depicted in brown/white, while hydrophilic regions are shown in blue. Pink dashed dots represent non-covalent interactions between alkyl groups; π-CH interactions in orange; π-π interactions in green; and hydrogen bonds in blue dotted lines. For **2s**, a particular sulfur-π interaction in gray is observed.

It is observed that the calculated interactions of molecules **1s, 1t**, and **1u** with FabI in this crystal are higher than those of **4alk**: *E_int_* = −8.16, −8.09, and −9.73 kcal/mol, respectively, due to the improved shape complementarity arising from the increased hydrophobicity of this FabI conformation. This increase in volume allowed the insertion of the two geranic acid fragments of **1u**, which increased the interaction energy by more than 2 kcal/mol. This effect also occurs in the binding of **2u**; the two geranic acid fragments fit optimally into the hydrophobic pocket. **2s** had a higher binding energy than in **4alk**, as in **2u**, where both compounds maintained a similar binding mode in this FabI conformation, inserting the geranic acid fragment. For **2t**, binding occurred via insertion of the berberine scaffold into the lipophilic pocket, which may explain the small decrease in the *E_int_* value. The interaction profile of each complex is shown in Figure 10.

Although **1s**, **1t**, and **1u** bind in a similar way, inserting the geranic acid fragment into the hydrophobic pocket (Tyr157, Ala198, Val201, Phe204, and Ile207), the binding mode of the berberine scaffold differs, especially for **1s**, which interacts with Met99 and Leu102. Meanwhile, the berberine scaffold of **1t** and **1u** interacts with Arg43 and Leu196. In this binding mode, the berberine scaffold adopts an orientation similar to that observed in compounds **2s** and **2u**. In contrast to **2t**, which interacts with the same residues but at different sections of its structure, this is the berberine scaffold with Tyr157, Ala198, Val201, Phe204, and Ile207, and the geranic acid with Arg43 and Leu196.

Some of the hybrid compounds **1u**, **2u**, and **2t** showed *E_int_* values of −9.73, −9.86, and −9.62 kcal/mol, respectively, which are higher than the corresponding *E_int_* values of the co-crystallized FabI inhibitors, E9P and kalimantacin A, which exhibited *E_int_* values of −9.59 and −8.57 kcal/mol, respectively. Notably, for compounds **2u** and **2t**, the lipophilic acid chains were positioned within the binding cavity, in an analogous manner to the inhibitor kalimantacin A. The referred hybrid compounds interact with the same residues (Leu102, Tyr147, Gln155, Asn156, Tyr157, Met160, Pro192, Ala198, Val201, Phe204, and Ile207) as the so-called left side of kalimantacin A [15].

In summary, the *E_int_* values for the best poses of our designed compounds that interact more strongly with the FtsZ and FabI targets of *S. aureus* are displayed in Table 6.

Table 6 shows that, depending on the crystal structure used for each biological target, the molecular docking results vary. These changes can be related to the ligand’s binding mode, which depends on the molecular shape of the protein’s binding site (cavities) and on the number and types of intermolecular interactions associated with cavity modifications. Our group has previously used these approximations in other systems, yielding similar conclusions; the structural variability of the biological target is key to robust molecular docking analysis [23,30,36,41].

### 2.5. Physicochemical Properties Analysis of Berberine Hybrid Compounds

**Figure 11:**
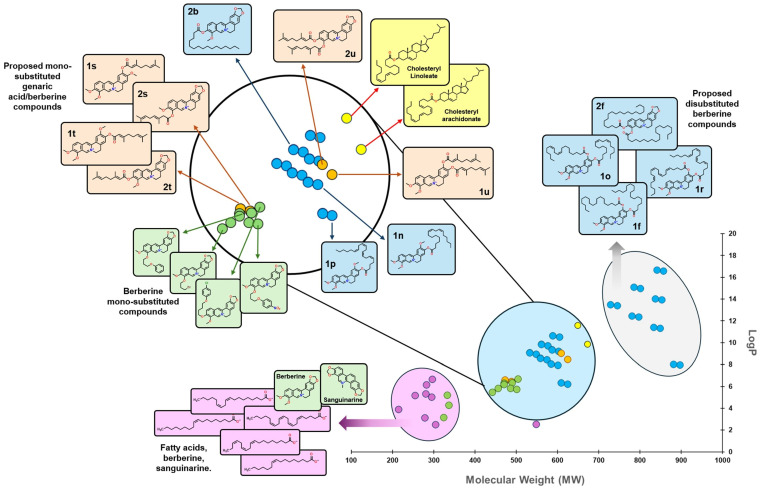
Molecular weight (MW) vs. *Log*P plot for berberine, fatty acid derivatives, and proposed new hybrid compounds. Lipophilic acids are highlighted in purple dots, while green dots represent berberine mono-aryl derivatives. Yellow dots indicate two known anti-staphylococcal cholesterol derivatives. Cyan dots are shown for several mono- and di-substituted berberine-lipophilic acid hybrid compounds. The orange circles highlight the most promising candidates **1s**, **1t**, **1u**, **2s**, **2t,** and **2u**, as determined by molecular docking results.

In silico ADMET properties were examined for the new berberine hybrid molecules alongside known compounds with documented antibacterial activity. Lipophilicity, a crucial property in drug development, influences a drug’s affinity for non-polar environments and its ability to permeate cell membranes. Typically, lipophilicity increases with molecular weight, especially with the addition of fatty acid alkyl chains. According to Lipinski’s rules, drug candidates usually have *LogP* values between 0 and 5, although some compounds exceed this range yet remain bioactive [42]. In this study, compounds **1a-x** and **2a-x** are predicted to have *LogP* values from 6.22 to 16.67, reflecting their high lipophilicity due to multiple alkyl chains. Figure 11 illustrates the correlation between *LogP* and molecular weight, showing that six promising molecules fall within the range of others with known antibacterial activity. For optimal oral bioavailability, TPSA values ≤ 140 Å^2^ are preferred [43]. The six top candidates—**1s**, **1t**, **1u**, **2s**, **2t**, and **2u**—have TPSA values ranging from 57.87 to 74.94 Å^2^, consistent with their lipophilic profiles. The analyzed cholesteryl derivatives display TPSA values around 26.3 Å^2^, marking a threshold for fatty acid derivatives below which antibacterial activity remains effective.

**Figure 12:**
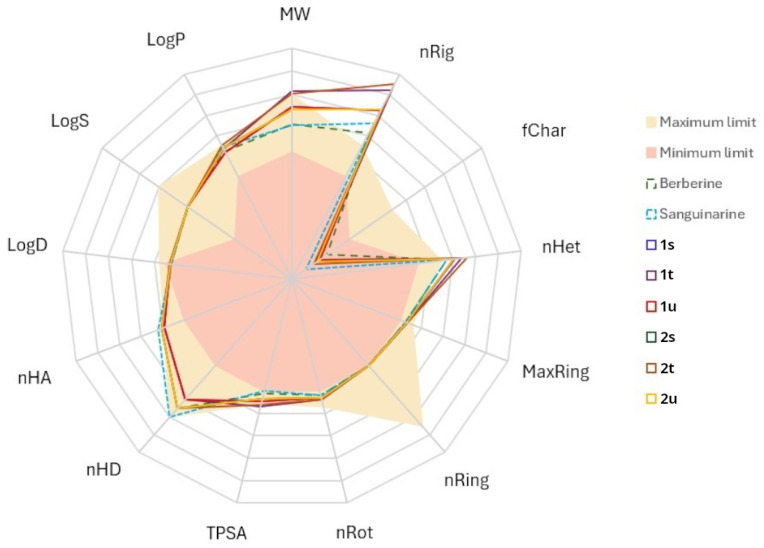
Radial plot of molecular descriptors related to the pharmacokinetics of berberine, sanguinarine, and **1s**, **1t**, **1u**, **2s**, **2t**, **2u**. *Log*S: logarithm of aqueous solubility; *Log*P: logarithm of the water/octanol partition coefficient; MW: molecular weight; nRig: number of rigid bonds; nRing: number of ring structures; nRot: number of rotatable bonds; nHD: number of hydrogen donors; nHA: number of hydrogen acceptors; TPSA: topological polar surface area; *Log*D: *Log*P at physiological pH 7.4; MaxRing: number of atoms in the largest ring; fChar: formal charge; nHet: number of heteroatoms.

Comparative physicochemical properties of **1s**, **1t**, **1u**, **2s**, **2t**, **2u**, and the berberine-based core are shown in Figure 12, in which only the newly proposed compounds exhibit a high number of rigid bonds (nRig).

Disubstituted berberine candidates are likely to be highly lipophilic, which could decrease their bioactivity. The predicted *Log*D (*log*P at pH 7.4) values for compounds **1s**, **1t**, **2s**, and **2t** range from 4.1 to 4.2, while ideal values are between 1.0 and 3.0, indicating a favorable ADMET profile. Most of these molecules also exhibit a high number of rotatable bonds (nRot up to 38), whereas drug candidates typically have 0 to 11. Promising compounds such as **1s**, **1t**, **1u**, **2s**, **2t**, and **2u** have nRot values between 7 and 14, fitting within the acceptable range. Additionally, due to the high count of rigid bonds, these molecules are predicted to be relatively inflexible.

Based on in silico toxicity predictions, the oral rat acute toxicity LD_50_ values of **2t** ranged from 1140 to 1461 g/kg (2.413 to 3.092 mol/kg), with the highest LD_50_ value. Although this compound ranks among the most toxic derivatives in the series, the LD_50_ falls within the grams-per-kilogram range, indicating a wide safety margin and, consequently, low in vivo toxicity. These compounds tested negative in the AMES toxicity assays, indicating they are nonmutagenic and noncarcinogenic. They also showed no signs of hERG I, hepatotoxicity, skin sensitization, or Minnow toxicity, although all compounds were predicted to inhibit hERG II. Additional details are available in Tables S5 and S6. Nonetheless, toxicological testing is necessary to fully evaluate the safety of these molecules. Molecular descriptors and in silico determination of parameters re-lated to the ADMET values of FtsZ and FabI inhibitors, and designed berberine derivatives are shown in Tables S7 and S8.

## 3. Discussion

The discovery of new antibacterial drugs faces an unprecedentedly high failure rate and escalating costs, making it essential to optimize each stage of the development process to maximize the chances of success [43]. In this context, computational chemistry has become an indispensable tool in the early phases of drug discovery. Structure-based virtual screening, molecular docking, and molecular dynamics simulations enable the evaluation of the binding affinity of a large number of candidate compounds for a given biological target before their chemical synthesis [44,45]. This approach acts as a virtual filter that prioritizes molecules with greater steric and energetic complementarity, thereby significantly increasing the hit rate. For instance, recent studies have demonstrated that integrating these techniques with machine learning has improved hit rates by up to 90-fold compared with high-throughput experimental screening, while also identifying active compounds that might otherwise have been overlooked [46].

Structure-based drug design is a common approach when the biological target’s structure is known, often surpassing ligand-based methods. Molecular docking plays a crucial role in this process and relies heavily on the accuracy of available structural data, such as crystal or NMR structures. The flexibility of binding pockets adds both robustness and complexity to the design process. To address these challenges, various strategies have been employed, including molecular dynamics simulations to sample different receptor conformations—especially when only limited structural data are available [47]. Another method involves using multiple receptor structures derived from crystal complexes with different ligands [48], as demonstrated here, where the lipophilic region of the FtsZ and FabI binding pockets exhibited structural variations depending on the ligand (inductive effect). Conversely, ligand-based drug design aims to optimize the molecular properties of a lead compound to enhance biological activity, even without knowing the biological target [49]. Ultimately, the most successful drug design efforts often combine both strategies [50], as we have done in this work.

FtsZ and FabI are two highly relevant antibacterial targets because of their essential roles in bacterial viability and their absence in mammals, which confers high selectivity and a low risk of toxicity [51]. Inhibiting FtsZ disrupts cytokinesis, leading to cell death [52,53]. Inhibiting FabI blocks fatty acid elongation, which is essential for membrane integrity [54], and has even shown synergism with conventional antibiotics to restore their activity against resistant strains [55,56]. These enzymes represent promising targets for developing new antibiotics to overcome the growing threat of multidrug-resistant bacterial infections.

Despite promising results for the calculated interaction energies of the hybrid compounds with the enzymes FtsZ and FabI, concerns remain regarding antibacterial activity.

Using the equations derived for FtsZ and FabI (Figure 4 and Figure 6, respectively), the potential MICs of the geranic acid-derived compounds were calculated, and the corresponding values are presented in Table S9. For FabI, linear regression analysis of derivative compounds, based on IC_50_ values for different fatty acids, indicates that compounds **1u** and **2t** are closest to the previously reported MIC values of known inhibitory fatty acids [16]. For compound **1u**, the predicted IC_50_ was 0.159 mM, which is lower than reported for linoleic acid (0.2 mM) [16]. Compound **2t** exhibited a predicted MIC of 0.188 mM, suggesting it could inhibit the enzyme similarly to linoleic acid. These values fall within the range reported for fatty acids with the highest affinity for FabI, 0.200 mM for oleic acid and 0.80 mM for linoleic acid. Furthermore, the IC values predicted by linear regression for compounds **1u** and **2t** correlate with their respective predicted *E_int_* values of −9.73 and −9.48 kcal/mol, respectively.

Compounds **1u** and **2t** remained within the range of the most active fatty acid inhibitors against FabI. Adding two geranic acid chains at the R_1_ and R_2_ positions of the berberine scaffold increased lipophilicity, improving the predicted IC_50_ from 0.456 mM (**1s**) to 0.159 mM (**1u**). This improvement was accompanied by an increase in *E_int_* from −8.16 to −9.73 kcal/mol. Likewise, adding two geranic acid chains at the R_3_ and R_4_ positions improved the IC_50_ from 0.188 to 0.146 µM and decreased *E_int_* from −9.48 to −9.86 kcal/mol. These findings support the initial hypothesis that adding lipophilic acid fragments at the R_1_, R_2_, R_3_, and R_4_ positions enhances inhibitory activity.

Notably, among the six top-performing derivatives, the compounds with the best *E_int_* values toward FabI were those bearing two geranic acid lipophilic fragments, compounds **2u** and **1u**, with *E_int_* values of −9.86 and −9.73 kcal/mol, respectively. These values are close to those reported for fabimycin, whose *E_int_*, determined by ITC, is −11.36 kcal/mol. Fabimycin has been shown to form predominantly hydrophobic interactions within the same binding site [57], as the hybrid compounds herein designed.

For FtsZ, **1u** has an MIC of 0.254 mM, whereas **1t**, bearing a modification at R3 of the berberine core, shows an MIC of 0.013 mM. This difference may be related to their *E_int_* values: **1u** has −9.21 kcal/mol and **1t** has −10.57 kcal/mol. A similar trend is observed for modifications at positions 8 and 9 of berberine. In the case of FtsZ, one of the most affine compounds was **2s**, with a MIC value of 0.012 mM as determined by linear regression analysis. Compared to inhibitor B (Figure 1), whose MIC value is 0.038 mM [13], compounds **1t**, **2s**, and **2t** exhibited higher predicted in silico IC_50_ values, 0.013, 0.012, and 0.017 mM, respectively. Although these findings require further in vitro validation, the *E_int_* values of the best-performing candidates correlate with their predicted IC_50_ values against FtsZ. The addition of two geranic acid chains at the R_1_ and R_2_ positions was more favorable in terms of IC_50_ improvement when comparing compounds **1s** and **1u**, with values increasing from 1.297 to 0.2542 mM, and *E_int_* changed from −8.57 to −9.21 kcal/mol. However, for compounds bearing modifications at the R_3_ and R_4_ positions, the addition of the lipophilic fragments led to a decrease in inhibitory activity, as evidenced by an IC_50_ increase from 0.012 to 0.040 mM for compounds **2s** and **2u**, and an *E_int_* increase from −10.59 to −10.44 kcal/mol, respectively. Therefore, for FtsZ, the proposed hypothesis is satisfied only when modifications are introduced at positions R_1_ and R_2_.

The fact that the increase in in silico affinity, as determined by *E_int_* and predicted IC_50_ values against FtsZ and FabI, was maintained upon introducing modifications at the R_1_ and R_2_ positions indicates that adding lipophilic fragments at these positions to synthesize potential antibacterial agents is plausible.

Numerous compounds derived from natural sources, as well as early-generation synthetic analogs, exhibit MIC values ranging from 0.143 to 0.286 mM against Gram-positive bacteria, including *S. aureus*. Although these concentrations are elevated relative to those of established clinical antibiotics (MIC < 0.029 mM), they are nonetheless regarded as viable leads for further medicinal chemistry refinement, especially when the compounds exhibit novel mechanisms of action or low cytotoxicity [57,58].

The QSAR models presented here were derived from experimental MIC values and interaction energies of berberine, its derivatives, and lipophilic acids. Consequently, their applicability is confined to these molecular frameworks. A notable limitation is the lack of field-based analyses and structural alignments; while shape and chemical complementarity can estimate interaction energies, these models do not determine the binding conformation. However, the statistical validation confirms their predictive reliability, consistently showing that lower MICs are associated with more negative *E_int_* values. Additionally, these models could be enhanced by including additional descriptors, particularly those related to conformational flexibility, given the lipophilic nature of the acid fragments.

Regarding toxicity, it is also important to note that berberine is known to be toxic, and indirect evidence indicates that the catechol ring is responsible for this toxicity. In fact, palmatine, a berberine derivative without this ring, exhibits lower toxicity [59], but its abundance in plants is much lower than that of berberine. For this reason, modifying the catechol ring in berberine, as presented herein, represents a plausible strategy in the search for novel drugs based on plant-derived pharmacophores with lower toxicity and greater affinity for the key enzyme that ultimately triggers bacterial cell death.

Nevertheless, it is important to note that adding carboxylic acid fragments to the berberine structure can increase its *Log*P to the point that it becomes unsuitable for oral administration [60,61]. For this reason, various plant-derived compounds with promising properties are discarded from in vivo assays due to their poor permeability. Fortunately, there are several alternatives to address this problem. One of them is loading these molecules into delivery systems such as liposomes, micelles, and nanoparticles. Another one is increasing intestinal absorption, as in the case of piperidine [62,63].

The synthesis of the most promising compounds may pose a challenge. However, we previously reported the synthesis of **1u** and several related compounds via Steglich esterification. **1u** was obtained in 56%, and some related derivatives bearing different lipophilic acids afforded yields ranging from 30% to 50%, suggesting that their synthesis may be feasible [35]. Although the synthesis of **2s** has not been reported, various alkyl groups have been introduced specifically at the R3 position [13]. Additionally, berberrubine, a derivative in which the methyl group at carbon 9 is converted to a ketone, has been obtained via thermal dechloromethylation of berberine. This ketone could then be reduced to a hydroxyl group, thereby allowing the addition of geranic acid via either of the two previously mentioned routes [64].

Regarding the stability of the compounds in blood plasma and upon entry into bacterial cells, previous in vitro assays assessing the biological activity of berberine, fatty acids, and their derived compounds have shown that ester derivatives retain the biological activity, and it is higher than the separated components, berberine and lipophilic acids [32,35]. This represents stability against carboxylesterase action [27,28]. Even if carboxylesterase were to break the molecule into its components, berberine would retain its activity against FtsZ, and the lipophilic acids would retain their activity against FabI.

## 4. Materials and Methods

### 4.1. Hybrid Compounds Conformational Analysis

3D structures of all molecules in this study were constructed using Spartan 24 (Wavefunction, Irvine, CA, USA) [65]. Conformational analysis was performed using molecular mechanics theory with the MMFF force field to obtain the equilibrium conformer of each compound (cation). Once the equilibrium conformer was obtained, the conformational distribution was calculated using the same theoretical framework—molecular mechanics and the MMFF force field—while maintaining the 1+ charge (cation) and examining a maximum of 10,000 conformations. Once the conformational distributions were obtained, ovality values were calculated, and the conformations showing the greatest differences among them were retained for each molecule [66,67,68]. This same protocol was applied to the reference molecules used in this study: berberine derivatives synthesized by Sun et al. [13], berberine, sanguinarine, lipophilic acids, and FabI inhibitors.

### 4.2. Characterization of the Binding Pocket

The binding pocket was predicted using the ProteinPlus server https://proteins.plus (accessed on 22 February 2023) [60,61,62,63,64,65,66,67,68,69,70], which includes the DoGSiteScorer tool that identifies pockets and subpockets [71,72,73].

### 4.3. Molecular Docking

For the molecular docking calculations, three conformers per ligand were used as molecular seeds (starting points) for further population generation (conformers) within the Vina search algorithm [74]. For the iteration process, the scoring function implemented in the Vina program was empirical and used Gaussian 9 (Wallingford, CT, USA) to model hydrophobic, steric, and hydrogen-bond interactions.

To consider the flexibility of the receptor binding sites, molecular docking was performed on five different crystal structures: three FtsZ structures from *S. aureus* (**5xdt** [39], **6kvq** [36], and **3voa** [40]), co-crystallized with cofactor Ca^2+^ and ligands like fluoxapiprolin (**6kvq**) and TXA707 (**5xdt),** and one without any bound in the allosteric site (**3voa**). Two FabI structures from *S. aureus* (**6tbb** [15] and **4alk** [75]) bound to the NADPH cofactor were conserved during the docking experiments, along with the co-crystallized ligands E9P (**4alk**) and kalimantacin A (**6tbb**). The validation and docking experiments were performed using AutoDock Vina 1.2.3 (La Jolla, CA, USA) on the command line, with different parameters for each crystal (Table S10).

The protocol was considered validated when the RMSD was below 2 Å for each co-crystallized ligand. For all systems, the exhaustiveness parameter was set to 8, and 3 modes were returned (Figure S13). The energies of each docking result were recorded in a database, and the best candidates were filtered out for each case. These were grouped by protein and sorted by energy from lowest to highest to select the best candidates. For FtsZ, the order was based on **6kvq**, as its cavity conformation maximized contact with long aliphatic chains. For FabI, the energies obtained in **6tbb** were used for the previously mentioned reasons.

The best docking candidates were evaluated based on their interaction energies and analyzed with Discovery Studio Visualizer to understand the binding mode and interaction profile.

### 4.4. Molecular Properties

Molecular properties were calculated using the SwissADMET platform https://www.swissadme.ch/index.php# (accessed on 4 May 2025) from their SMILES codes; this platform provides parameters such as lipophilicity, hydrophilicity, MW, and other ADMET properties. These molecules were modeled in 2D using their respective SMILES codes in MarvinSketch 24, available at the same SwissADMET platform [76]. The prediction of the toxicity of the molecules analyzed was performed on the online platform pkCSM-pharmacokinetics https://biosig.lab.uq.edu.au/pkcsm/ (accessed on 9 February 2026), which is based on 10 parameters as follows: AMES toxicity, maximum tolerated dose (human), hERG I and II inhibition, oral rat acute toxicity (LD_50_), oral rat chronic toxicity (LOAEL), hepatotoxicity, skin sensitization, *T. periformis* toxicity, and minnow toxicity [77,78].

### 4.5. QSAR Model Determination

To determine the relationship between the interaction energy of the best berberine-derived candidates and that with lipophilic acids, a QSAR model was developed using alvaModel software 3.0 (Alvascience, Lecco, Italy). For these models, MIC was normalized by dividing it by the molecular weight, and *E_int_* was used as an independent variable. For the statistical validation, we used the following statistical parameters: coefficient of determination (R^2^), cross-validated test, the leave-one-out method (Q^2^), standard deviation (s), and Fisher test (F) 212 For both QSAR models, the MIC values of previously reported inhibitors were used: for FtsZ, berberine derivatives [13], and for FabI, lipophilic acids and another set of inhibitors [14,15,79]. Microsoft Excel (Redmond, WA, USA) from the Microsoft Office Suite was used to construct graphs.

## 5. Conclusions

Using berberine as a plant-derived pharmacophore to generate hybrid compounds with lipophilic acids represents a viable strategy in the ongoing search for novel drugs based on plant-derived pharmacophores that, in computational studies, exhibit lower toxicity and greater interaction energy with FtsZ and FabI. These key enzymes ultimately trigger bacterial cell death.

The hydrophobic properties of the hybrid compounds modeled here increased the interaction energy between the allosteric site of FtsZ and the active site of FabI. Furthermore, adding these chains broadens the range of physicochemical properties, including increased hydrophobicity and flexibility conferred by the carbon chains, which align with chemical and shape complementarity, respectively. This indicates a key consideration in drug design based on berberine and lipophilic acids: the addition of carbon chains should be carefully controlled, with a maximum length of 12 carbons, and should contain unsaturation.

In this context, including geranic acid fragments increased the computationally calculated interaction energy for both enzymes. Our research highlights the potential of **1u** and **2t** as candidates for further development targeting the FabI and FtsZ enzymes, respectively, based on their predicted activity and synthetic feasibility.

According to the molecular docking and QSAR models developed herein, the addition of lipophilic fragments of no more than 12 carbons to the berberine scaffold is a plausible strategy for synthesizing compounds with affinity for the FabI and FtsZ enzymes, potential antibacterial properties, and low toxicity (predicted by ADMET).

## Figures and Tables

**Table 1:** Chemical structures of berberine derivatives **1a-x** and **2a-x**. Oxygen and nitrogen are shown in red and blue, respectively.

	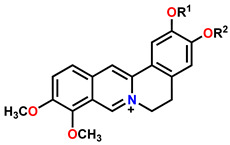			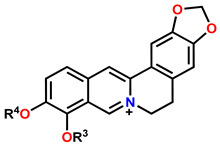	
Compound	R^1^	R^2^	Compound	R^3^	R^4^
**1a**	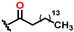	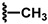	**2a**	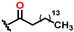	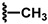
**1b**	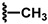	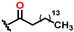	**2b**	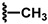	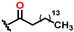
**1c**	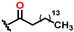	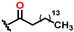	**2c**	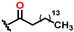	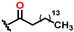
**1d**	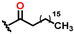	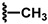	**2d**	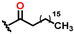	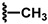
**1e**	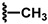	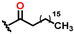	**2e**	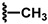	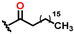
**1f**	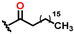	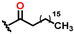	**2f**	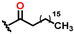	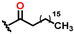
**1g**	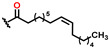	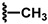	**2g**	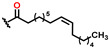	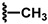
**1h**	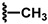	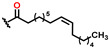	**2h**	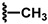	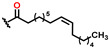
**1i**	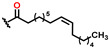	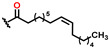	**2i**	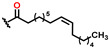	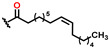
**1j**	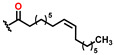	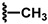	**2j**	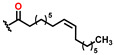	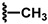
**1k**	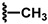	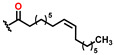	**2k**	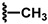	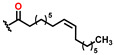
**1l**	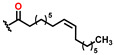	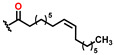	**2l**	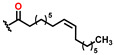	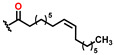
**1m**	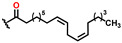	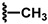	**2m**	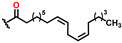	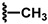
**1n**	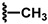	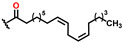	**2n**	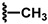	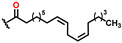
**1o**	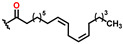	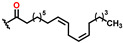	**2o**	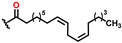	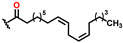
**1p**	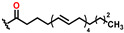	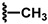	**2p**	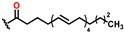	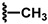
**1q**	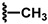	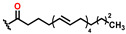	**2q**	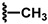	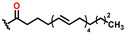
**1r**	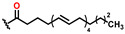	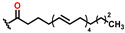	**2r**	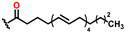	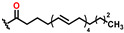
**1s**	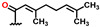	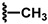	**2s**	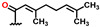	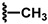
**1t**	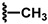	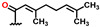	**2t**	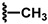	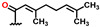
**1u**	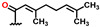	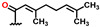	**2u**	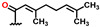	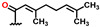
**1v**	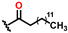	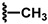	**2v**	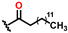	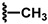
**1w**	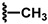	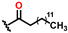	**2w**	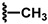	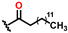
**1x**	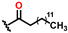	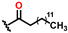	**2x**	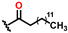	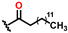

**Table 2:** Structural and Physicochemical Characteristics of the Allosteric Binding Pocket of FtsZ.

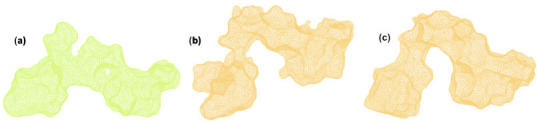							
Crystal Structure	Volume [Å^3^]	Surface [Å^2^]	Depth [Å]	Hydrophobicity Ratio	aPolar Ratio	Polar Ratio	Drug Score
**(a) 3voa**	757.00	868.86	20.65	0.28	0.60	0.31	0.84
**(b) 6kvq**	1019.01	1161.25	22.03	0.38	0.56	0.34	0.82
**(c) 5xdt**	974.66	1055.576	20.16	0.39	0.61	0.31	0.82

**Table 3:** Structural and Physicochemical Characteristics of the Allosteric Binding Subpockets of FtsZ (**5xdt**).

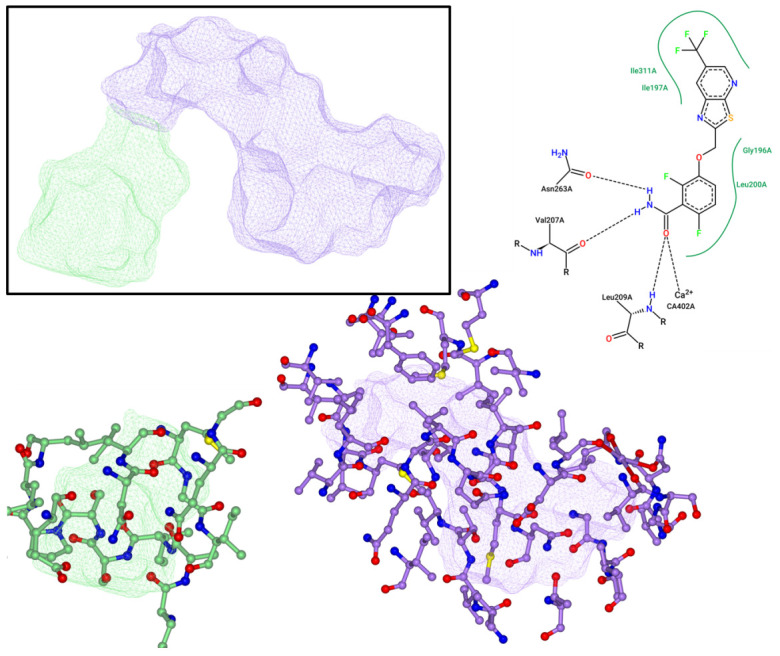							
PDB:5xdt	Volume [Å^3^]	Surface [Å^2^]	Depth [Å]	Hydrophobicity Ratio	aPolar Ratio	Polar Ratio	Drug Score
Subpocket 1 (purple)	672.83	707.96	20.16	0.42	0.64	0.31	0.79
Subpocket 2 (green)	301.82	441.12	11.52	0.37	0.56	0.33	0.48

**Table 6:** *E_int_* values (kcal/mol) of the molecular docking experiments of the berberine derivatives over the crystal forms of FtsZ (**3voa**, **6kvq**, and **5xdt**) and FabI (**4alk** and **6tbb**) of *S. aureus*.

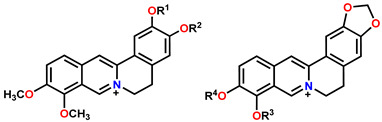						
Compound	Lipophilic Acid	3voa	6kvq	5xdt	4alk	6tbb
**2s**	R3-Geranic	−7.45	−9.89	−10.59	−8.15	−8.98
**1t**	R2-Geranic	−7.19	−7.79	−10.57	−7.05	−8.09
**2t**	R4-Geranic	−8.00	−8.54	−10.44	−9.62	−9.48
**2u**	R3-, R4-Geranic	−7.21	−7.87	−10.05	−9.20	−9.86
**1u**	R1-, R2-Geranic	−6.90	−7.93	−9.21	−6.90	−9.73
**1s**	R1-Geranic	−7.15	−10.47	−8.57	−8.72	−8.16
**2h**	R4-Palmitoleic	−6.83	−7.46	−8.52	−7.87	−8.50
**2q**	R4-Arachidonic	−6.07	−7.31	−8.06	−8.04	−8.99
**2n**	R4-Linoleic	−6.23	−6.46	−8.05	−8.34	−8.79
**2k**	R4-Oleic	−6.51	−6.75	−7.98	−7.82	−8.60
**2p**	R3-Arachidonic	−6.91	−7.52	−7.96	−8.66	−8.76
**2w**	R4-Myristic	−6.19	−7.17	−7.94	−8.24	−7.76
**1g**	R1-Palmitoleic	−5.89	−7.13	−7.88	−8.08	−8.19
**2m**	R3-Linoleic	−6.53	−7.86	−7.80	−8.39	−8.36
**2b**	R4-Palmitic	−6.13	−6.27	−7.74	−7.72	−8.01
**2e**	R4-Stearic	−6.24	−6.46	−7.72	−8.20	−8.10
**2j**	R3-Oleic	−6.07	−7.75	−7.70	−7.35	−7.77
**2a**	R3-Palmitic	−6.42	−7.28	−7.65	−7.84	−7.90
**2g**	R3-Palmitoleic	−6.52	−9.37	−7.54	−8.25	−8.50
**1p**	R1-Arachidonic	−6.09	−8.10	−7.52	−8.31	−8.47
**2v**	R3-Myristic	−6.78	−9.45	−7.47	−8.30	−7.58
**1q**	R2-Arachidonic	−6.50	−6.93	−7.43	−8.05	−8.96
**1v**	R1-Myristic	−5.78	−9.20	−7.40	−7.98	−7.18
**2d**	R3-Stearic	−6.32	−8.88	−7.32	−7.64	−8.67
**1m**	R1-Linoleic	−5.52	−7.69	−7.30	−8.16	−8.06
**2f**	R3-, R4-Stearic	−5.51	−7.38	−7.17	−6.61	−6.42
**1j**	R1-Oleic	−5.70	−6.86	−7.16	−7.75	−8.19
**1h**	R2-Palmitoleic	−5.93	−7.52	−7.14	−7.93	−8.01
**1e**	R2-Stearic	−5.35	−6.57	−7.08	−7.27	−8.05
**1w**	R2-Myristic	−5.83	−7.30	−7.06	−7.97	−7.94
**1b**	R2-Palmitic	−5.78	−6.91	−7.04	−7.73	−7.52
**1n**	R2-Linoleic	−5.80	−7.31	−7.02	−7.74	−8.36
**1d**	R1-Stearic	−5.91	−6.63	−6.99	−7.47	−7.52
**1a**	R1-Palmitic	−5.85	−8.63	−6.91	−7.66	−7.91
**1o**	R1-, R2-Linoleic	−6.32	−5.40	−6.88	−6.35	−6.46
**1k**	R2-Oleic	−5.84	−6.41	−6.60	−7.27	−8.47
**2r**	R3-, R4-Arachidonic	−6.23	−6.89	−6.56	−6.97	−7.41
**2i**	R3-, R4-Palmitoleic	−5.69	−8.06	−6.54	−6.68	−6.97
**1l**	R1-, R2-Oleic	−4.81	−5.94	−6.43	−6.77	−7.25
**1r**	R1-, R2-Arachidonic	−4.76	−7.76	−6.39	−6.50	−8.04
**2o**	R3-, R4-Linoleic	−5.32	−6.08	−6.36	−6.75	−7.60
**2x**	R3-, R4-Myristic	−6.38	−6.29	−6.34	−6.85	−6.93
**2c**	R3-, R4-Palmitic	−5.33	−7.25	−6.16	−7.13	−6.58
**1c**	R1-, R2-Palmitic	−5.84	−6.00	−6.09	−6.86	−6.90
**1x**	R1-, R2-Myristic	−5.10	−5.82	−5.93	−7.10	−7.12
**1i**	R1-, R2-Palmitoleic	−5.21	−5.95	−5.93	−6.90	−7.30
**1f**	R1-, R2-Stearic	−5.85	−5.68	−5.88	−6.54	−6.20
**2l**	R3-, R4-Oleic	−6.64	−5.78	−5.79	−6.48	−7.24
**TXA707**	-	-	-	−11.18	-	-
**Fluoxapiprolin**	-	-	−11.73	-	-	-
**E9P**	-	-	-	-	−9.59	-
**Kalimantacin**	-	-	-	-	-	−8.67

## Data Availability

Data can be obtained by contacting the corresponding authors.

## Supplementary Materials

The following supporting information can be downloaded at https://www.mdpi.com/article/10.3390/ijms27157035/s1.
