# Supplementary material for: Computationally Generated Plant-Derived Berberine-Based Hybrid Compounds as Potential Dual Binders to Staphylococcus aureus FtsZ/FabI Enzymes: A Ligand-Based Approach
Source: Int J Mol Sci. 2026 Aug 5;27(15):7035. doi: 10.3390/ijms27157035 (PMC13467142; doi:10.3390/ijms27157035)
Supplement: Supplementary file 1 [file ijms-27-07035-s001.zip › ijms-4391748-supplementary.pdf]

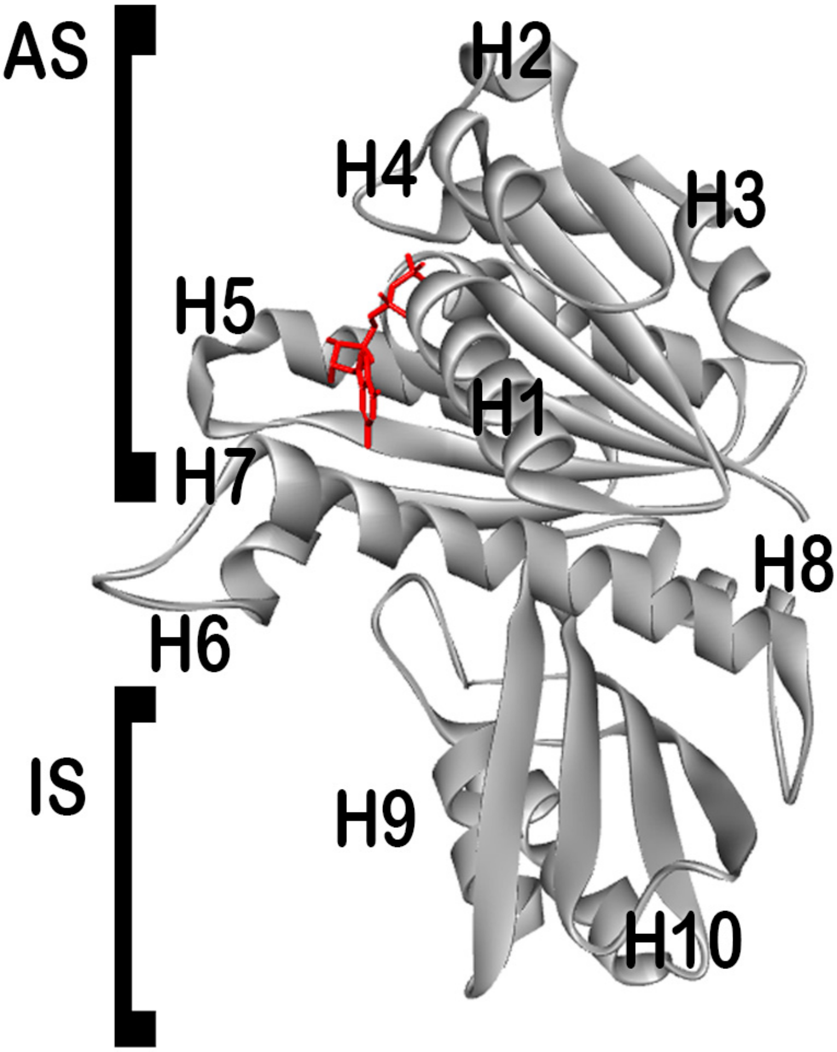

Figure S1. Secondary Structure of FtsZ enzyme. GDP molecule is represented as the red ligand. PDBID: 4DXD.

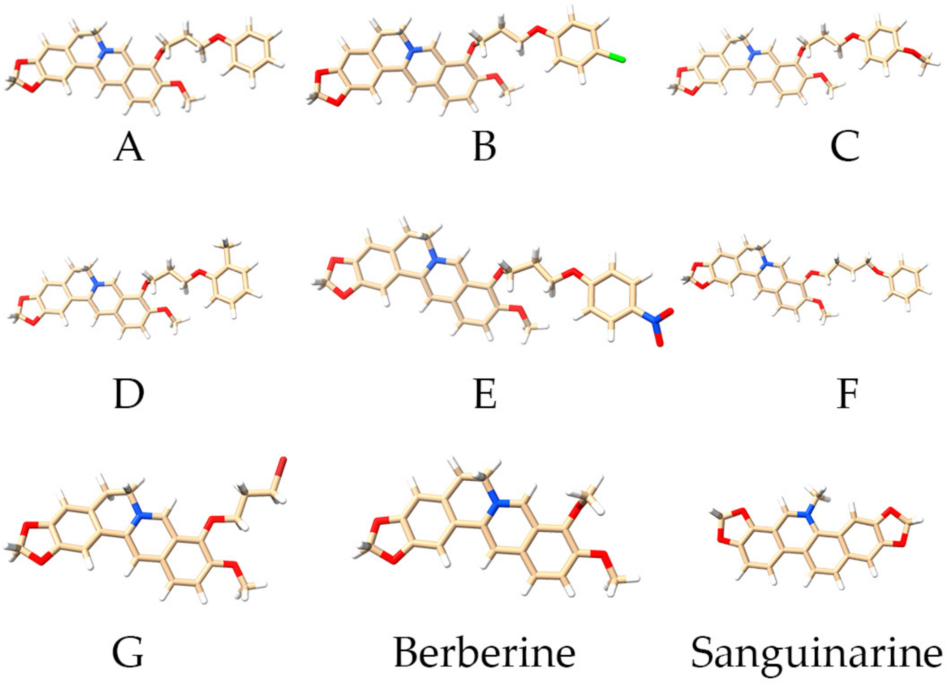

Figure S2. 3D representation of berberine and its 9-phenoxyalkyl-substituted derivatives synthesized by Sun et al. [11].

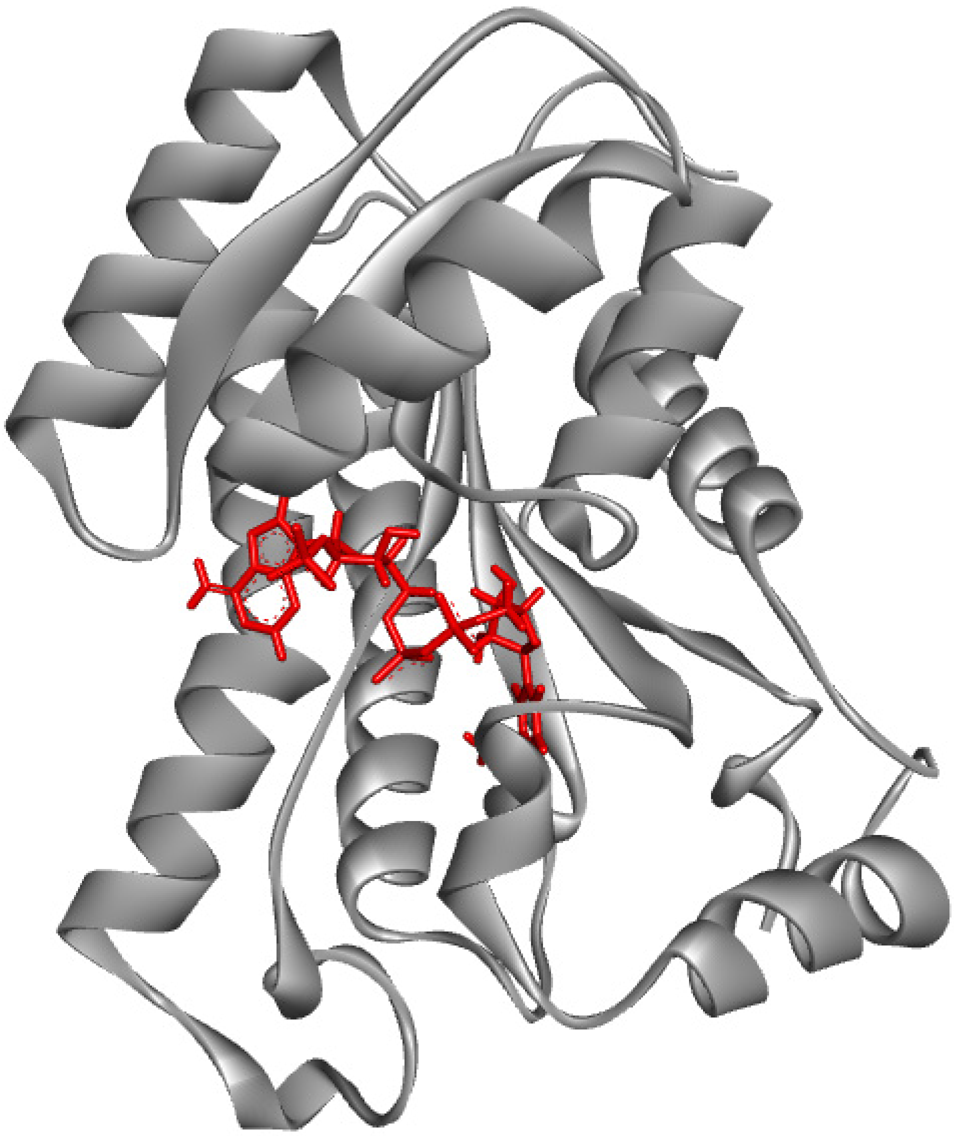

Figure S3. Secondary structure of FabI enzyme. NADP molecule is represented as the red ligand. PDBID: 4ALK.

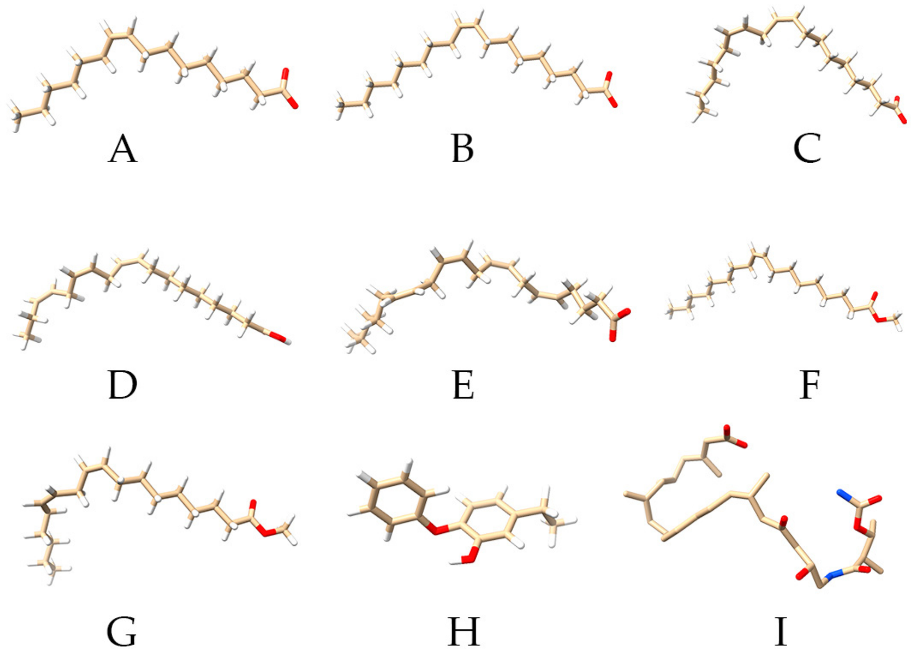

Figure S4. 3D representation of FabI inhibitors.

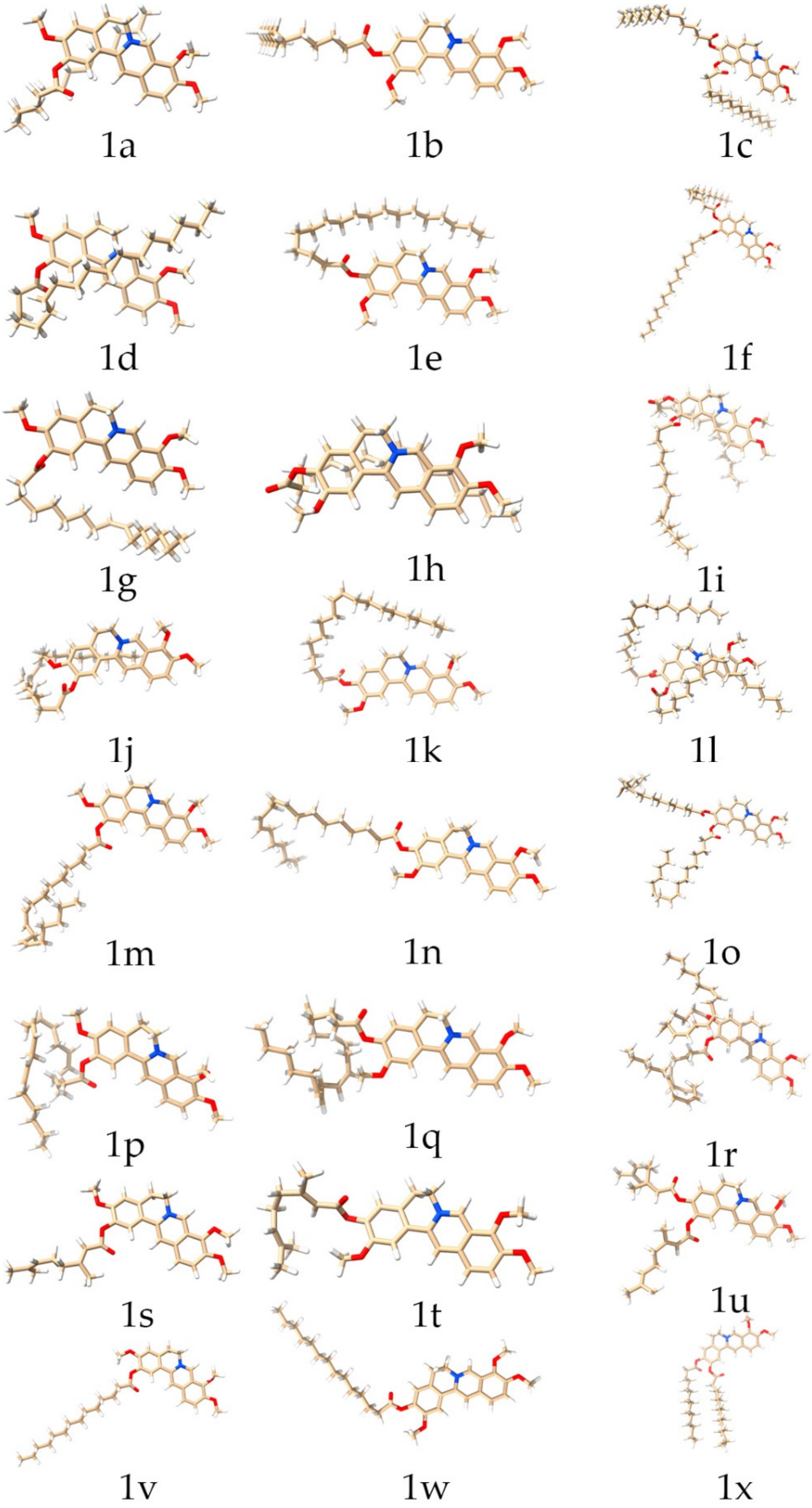

Figure S5. 3D representation of berberine 1-derivatives.

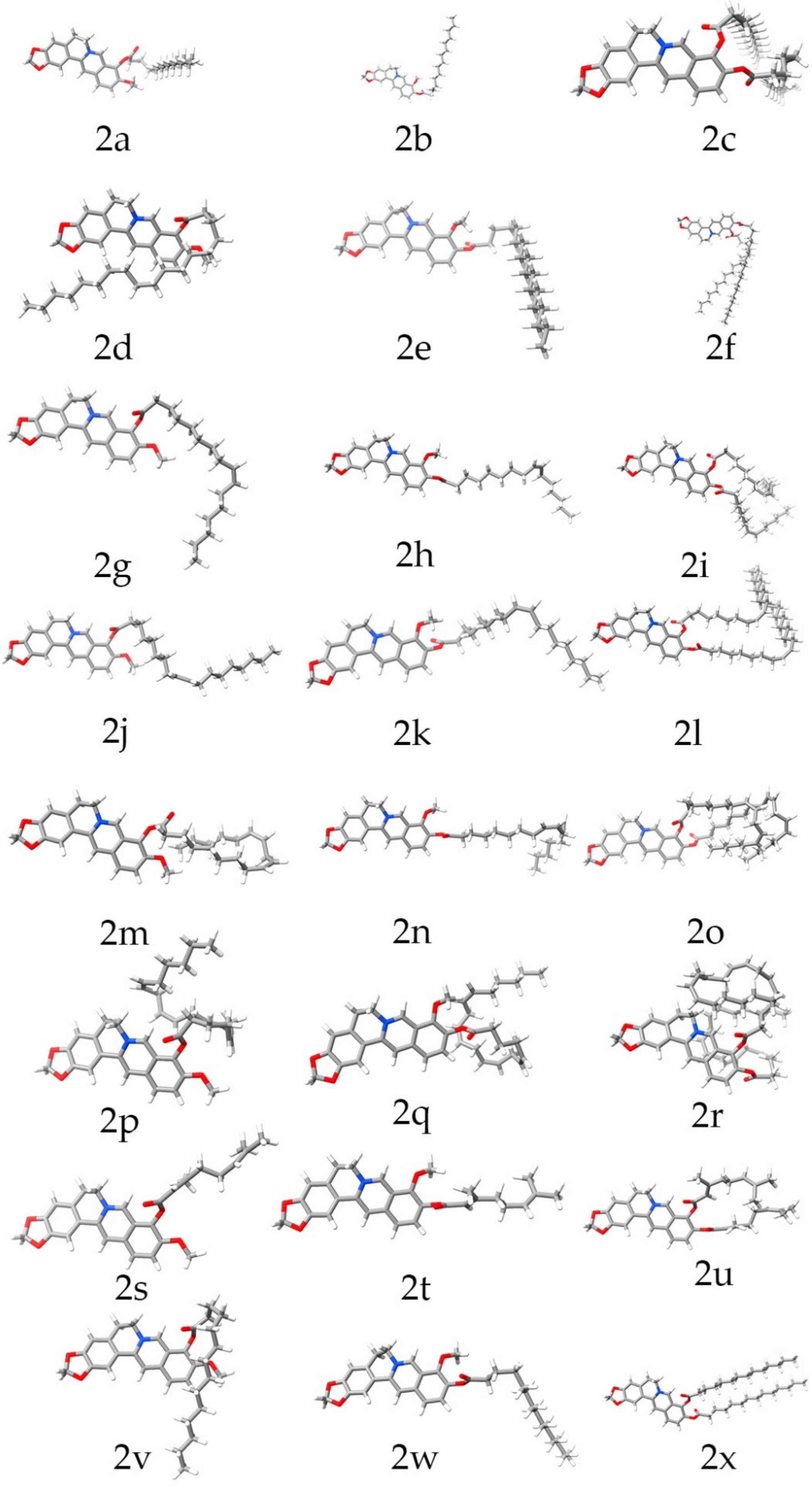

Figure S6. 3D representation of berberine 2-derivatives.

Table S1. Representative structures for conformer distribution of each berberine derivate and their ovality.

Mol.	Conformer a	Conformer b	Conformer c
**1a**	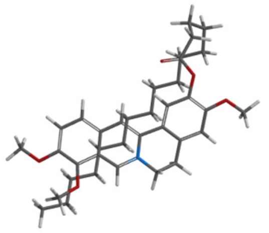 1.87	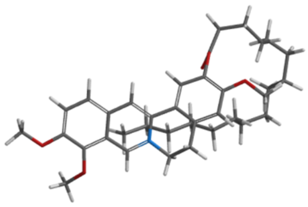 1.87	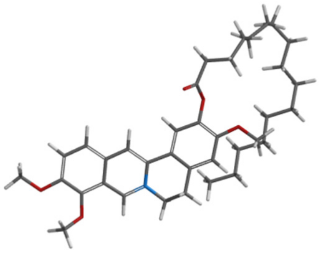 1.88
**1b**	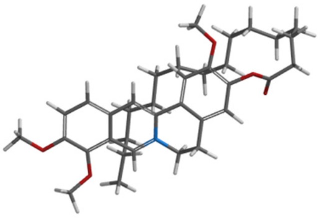 1.86	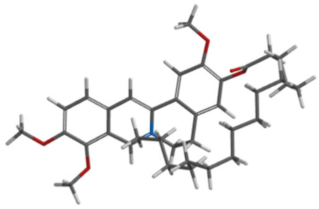 1.87	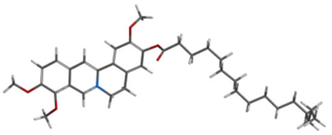 1.88
**1c**	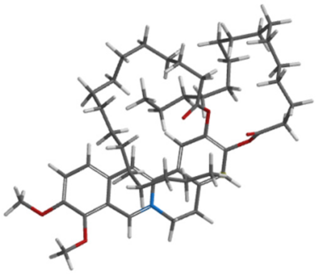 2.11	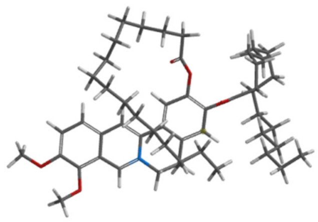 2.13	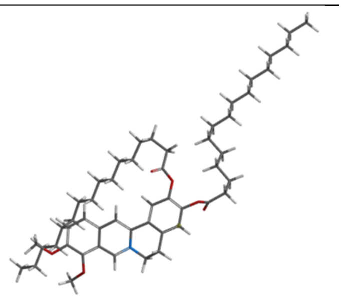 2.14
**1d**	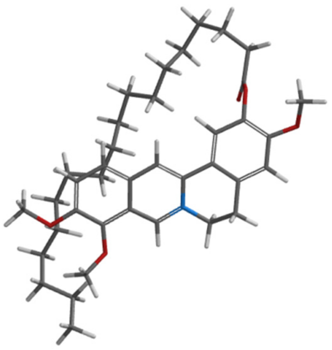 1.90	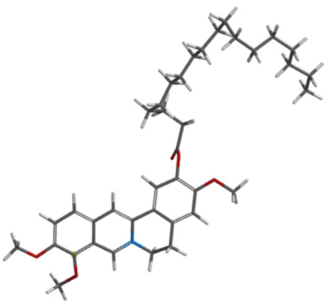 1.91	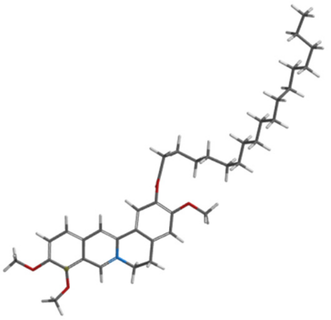 1.92

**1e**	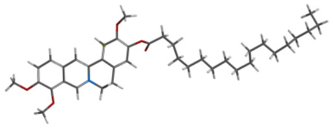 1.91	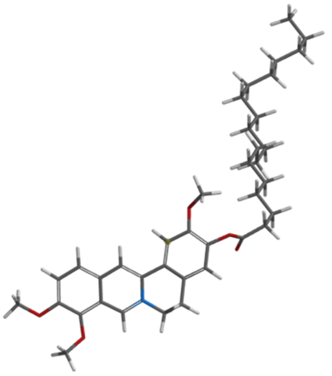 1.92	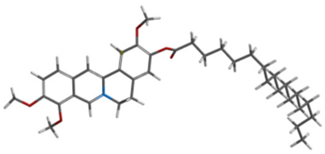 1.92
**1f**	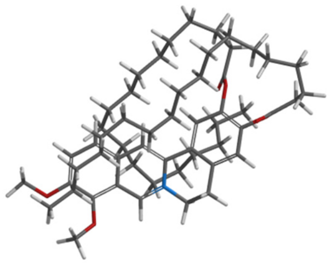 2.16	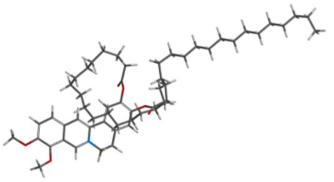 2.18	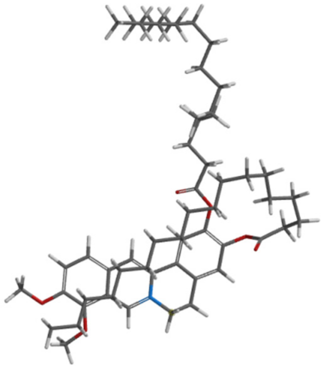 2.19
**1g**	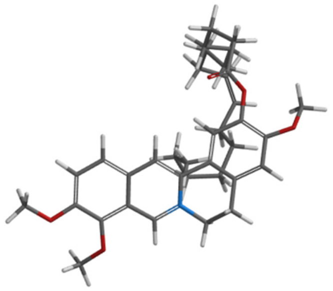 1.86	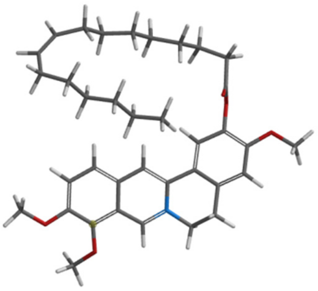 1.87	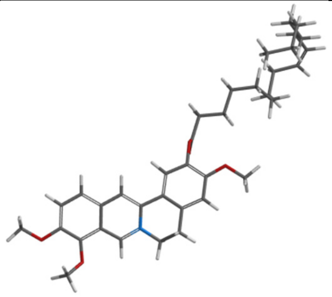 1.87
**1h**	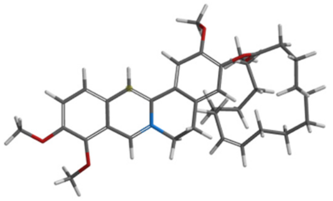 1.85	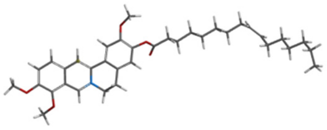 1.87	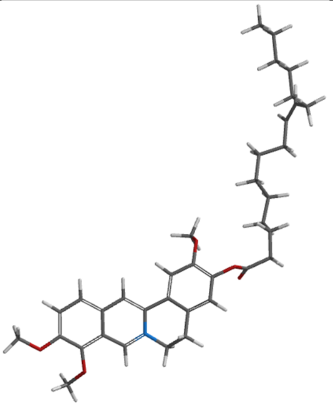 1.88

**1i**	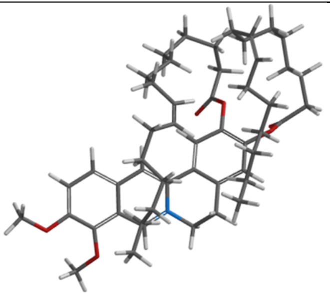<br>2.10	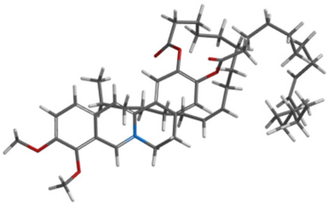<br>2.11	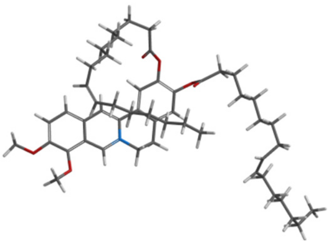<br>2.13
**1j**	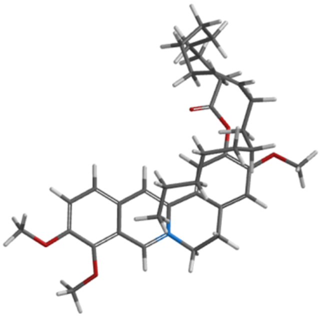<br>1.89	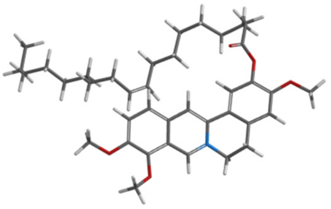<br>1.90	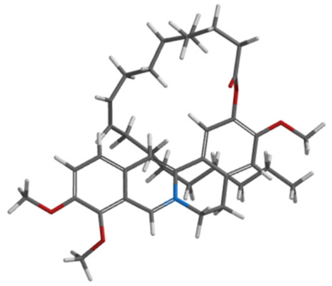<br>1.91
**1k**	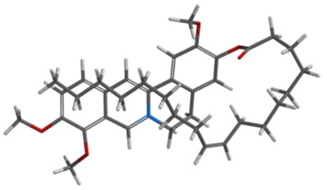<br>1.90	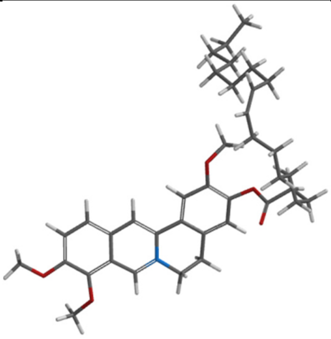<br>1.90	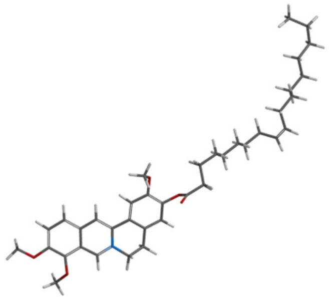<br>1.91
**1l**	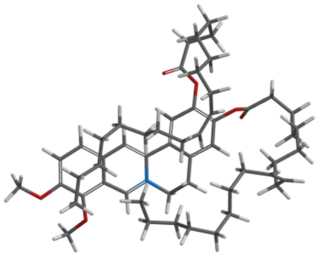<br>2.16	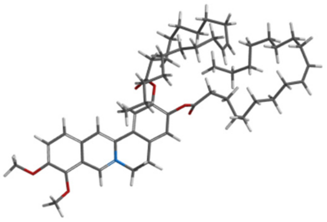<br>2.18	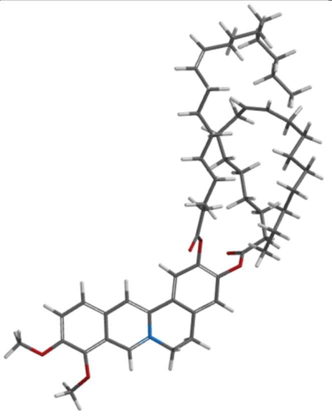<br>2.19

**1m**	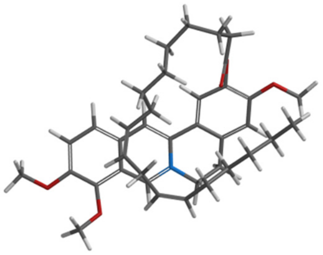 1.88	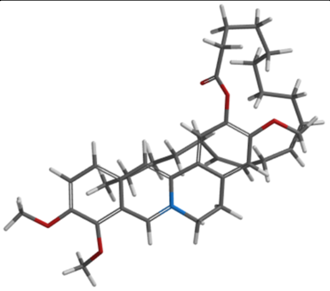 1.89	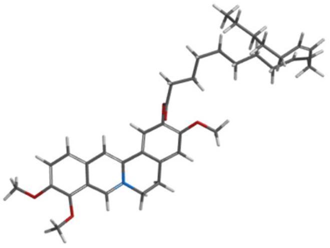 1.91
**1n**	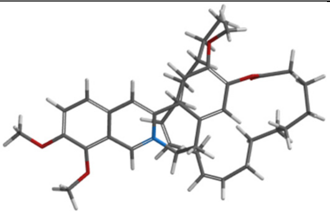 1.87	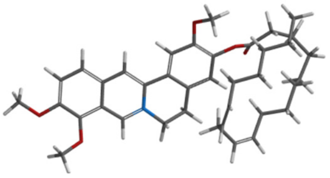 1.89	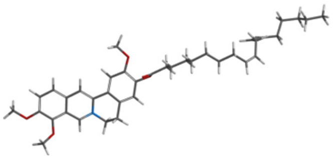 1.91
**1o**	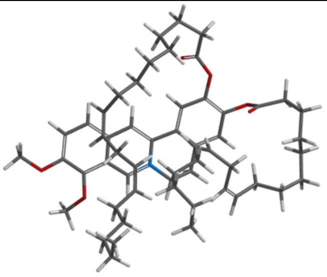 2.14	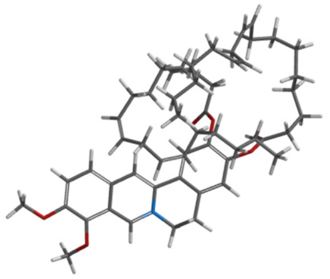 2.16	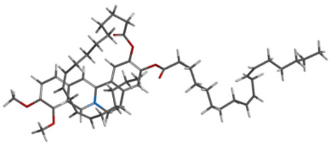 2.17
**1p**	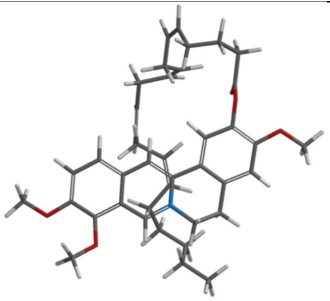 1.91	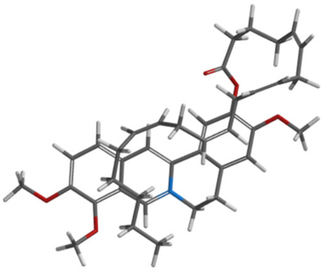 1.92	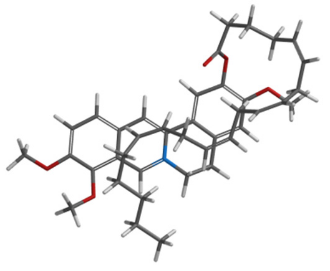 1.93
**1q**	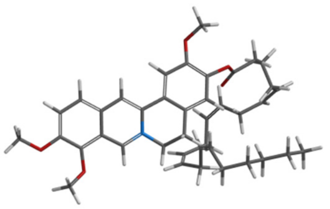 1.92	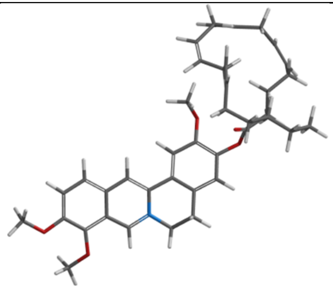 1.93	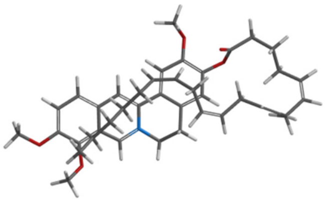 1.93

**1r**	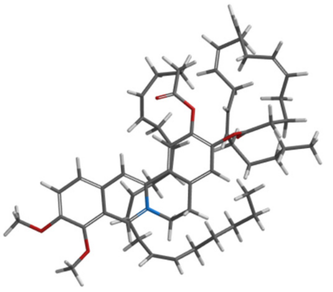 2.18	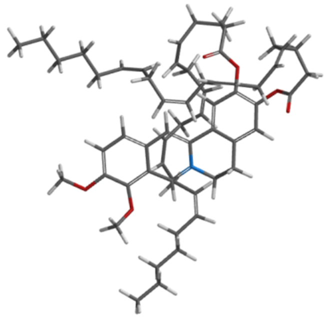 2.19	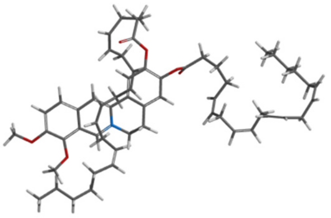 2.20
**1s**	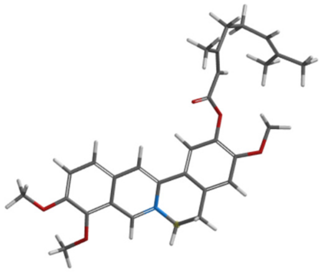 1.72	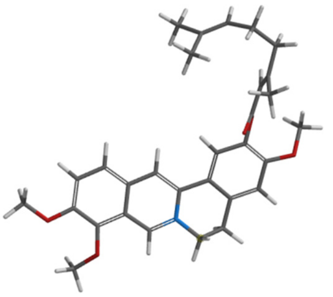 1.73	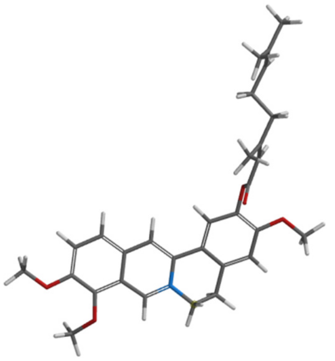 1.74
**1t**	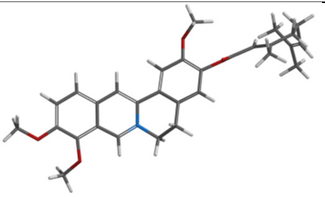 1.72	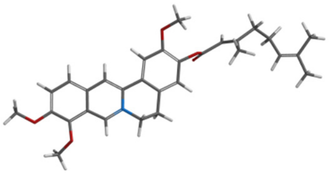 1.73	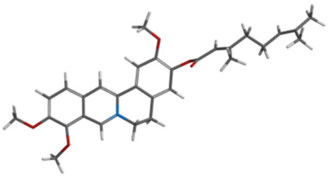 1.74
**1u**	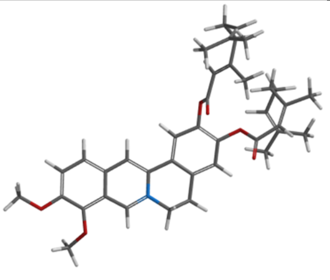 1.89	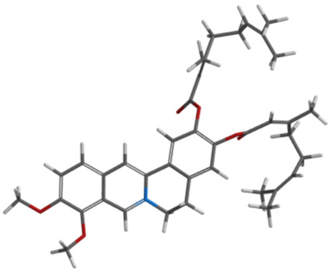 1.90	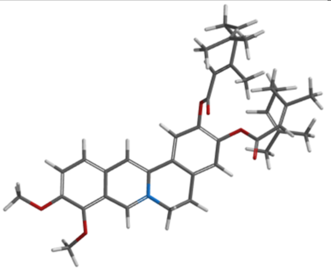 1.91
**1v**	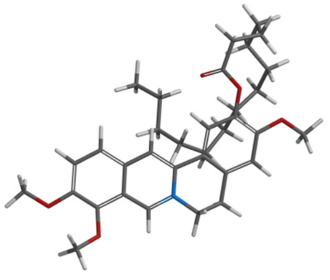 1.82	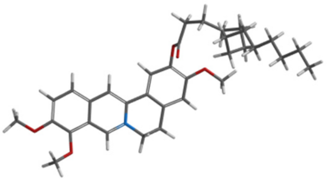 1.83	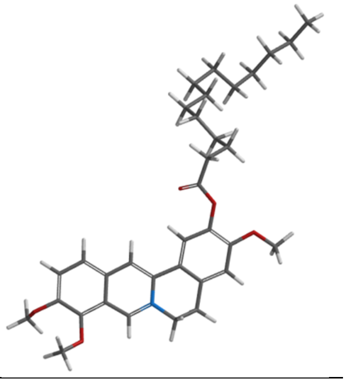

			1.84
**1w**	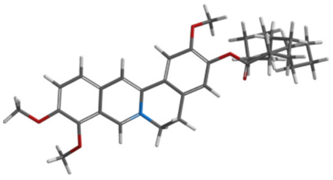 1.82	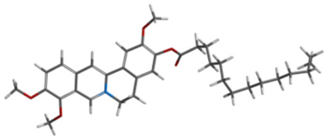 1.83	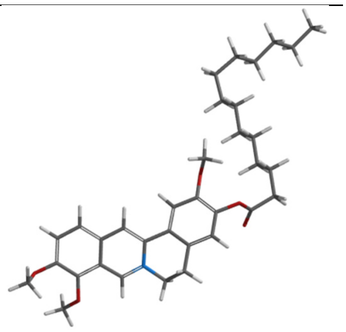 1.85
**1x**	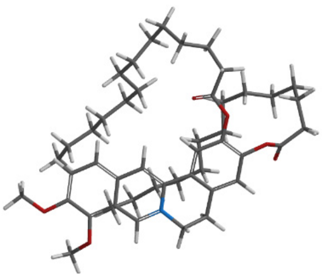 2.05	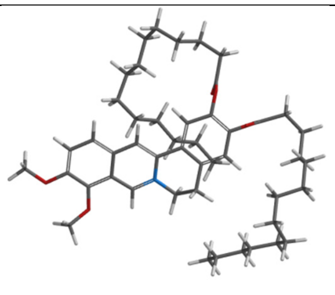 2.06	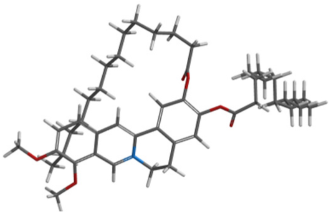 2.08
**2a**	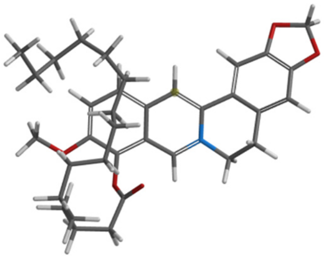 1.82	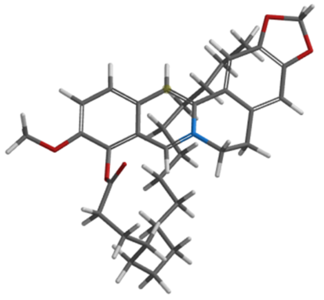 1.83	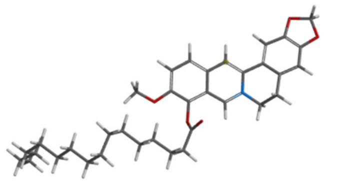 1.84
**2b**	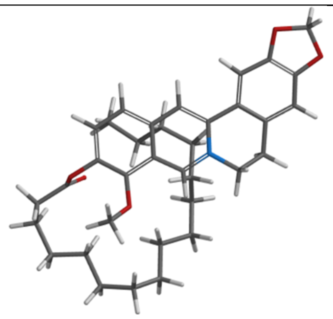 1.82	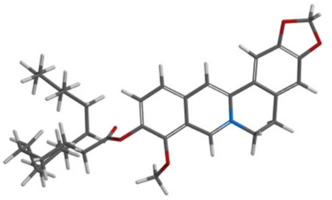 1.83	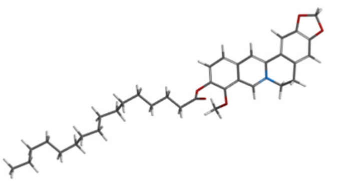 1.84

**2c**	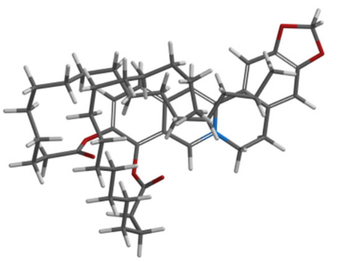 2.07	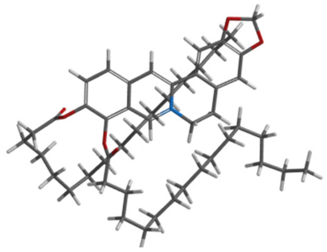 2.10	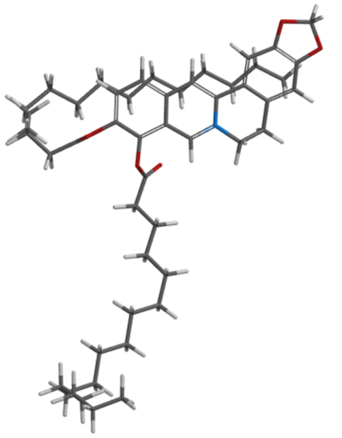 2.11
**2d**	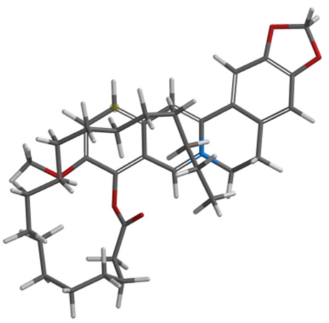 1.86	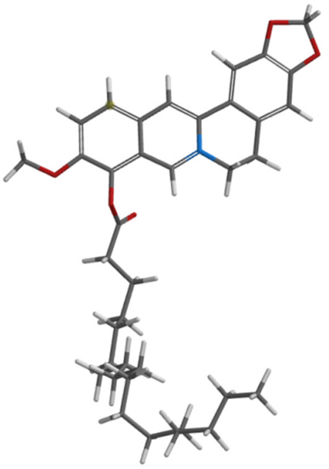 1.87	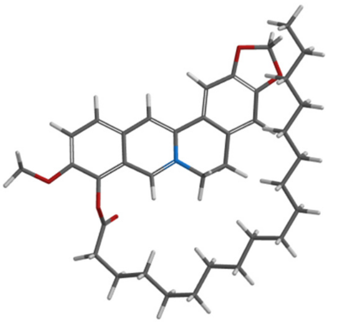 1.88
**2e**	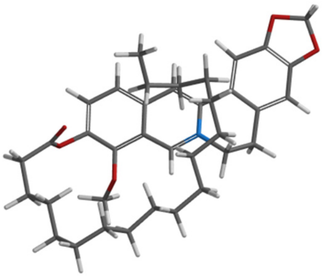 1.86	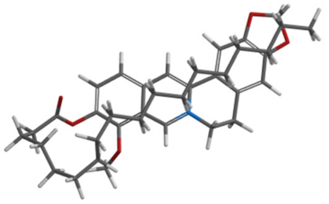 1.87	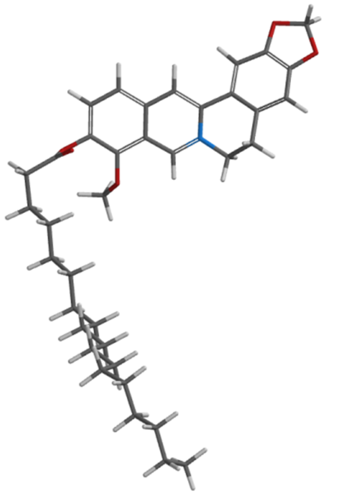 1.89

**2f**	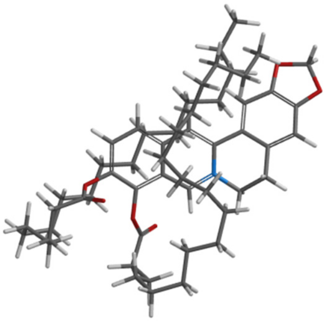 2.14	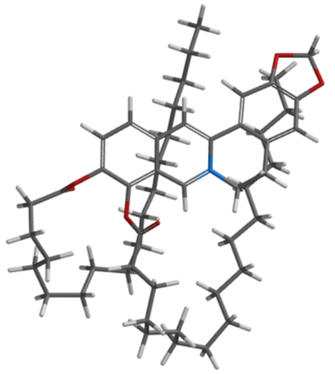 2.16	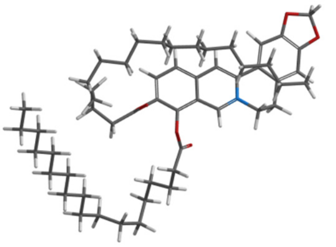 2.16
**2g**	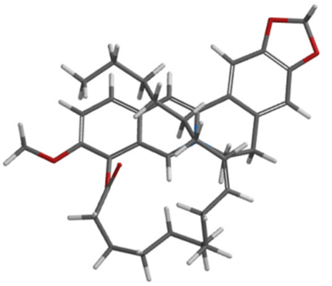 1.81	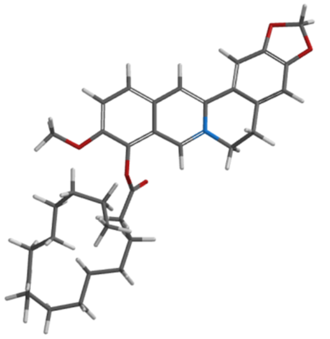 1.83	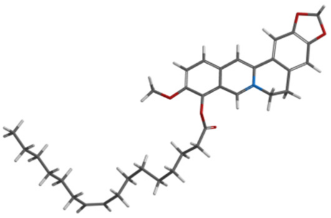 1.84
**2h**	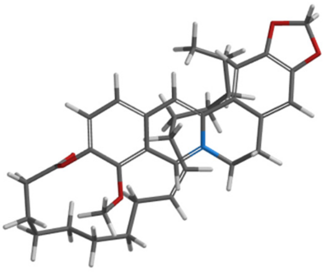 1.81	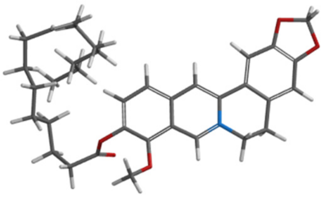 1.82	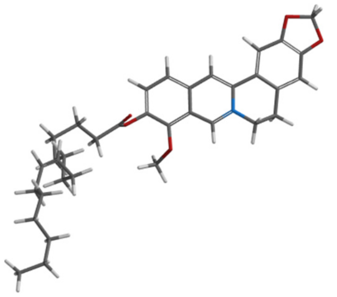 1.83
**2i**	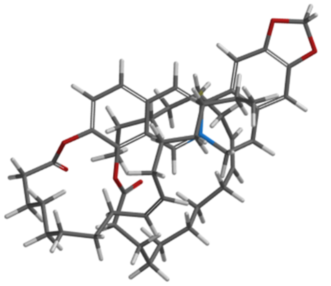 2.06	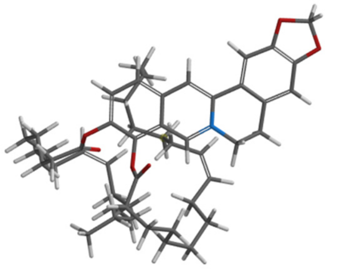 2.07	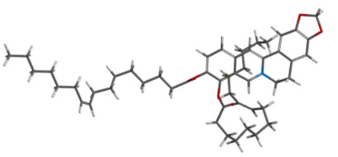 2.09

**2j**	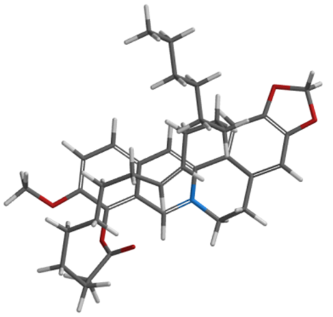 1.85	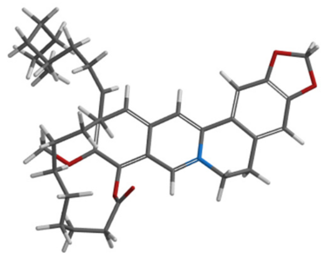 1.86	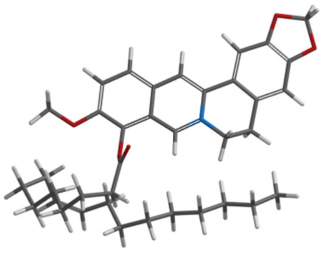 1.87
**2k**	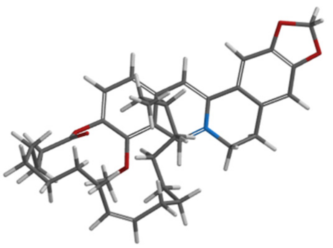	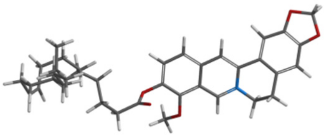 1.87	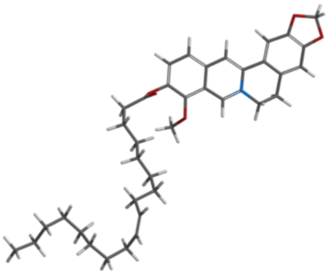 1.87
**2l**	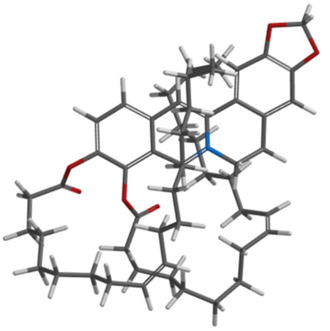 2.12	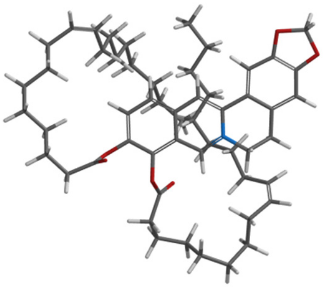 2.14	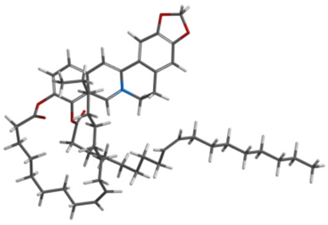 2.16
**2m**	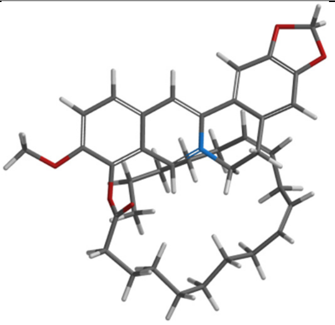 1.85	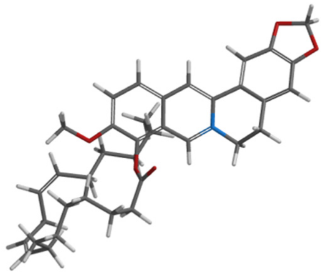 1.86	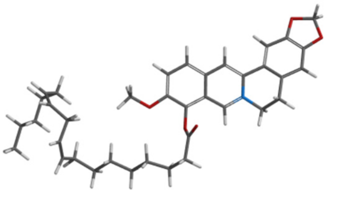 1.86

**2n**	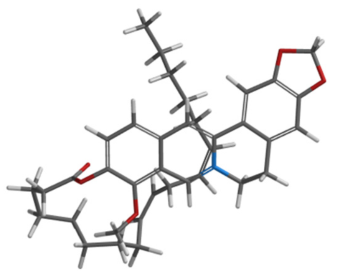<br>1.84	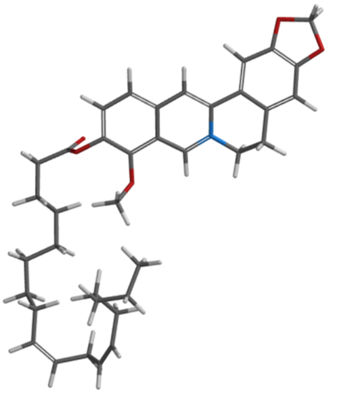<br>1.86	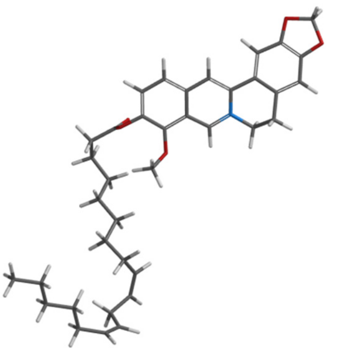<br>1.87
**2o**	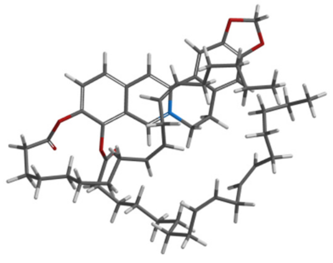<br>2.11	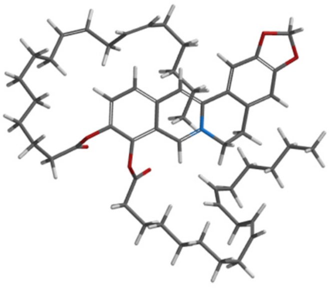<br>2.13	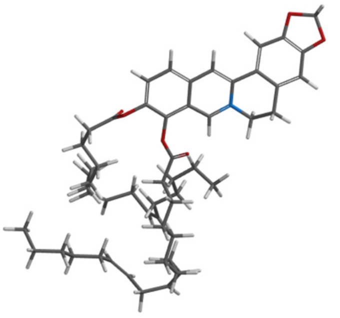<br>2.14
**2p**	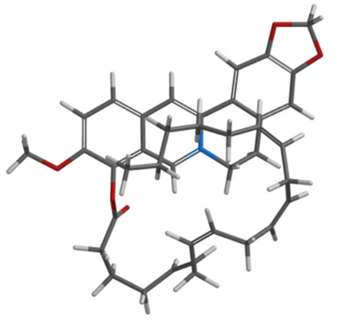<br>1.86	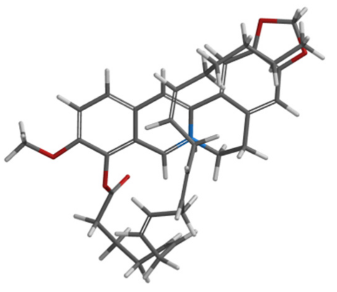<br>1.87	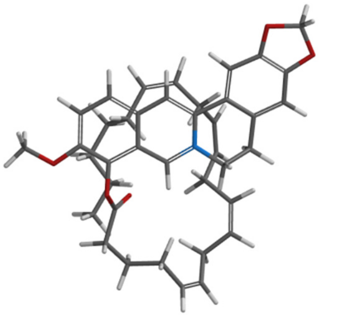<br>1.89
**2q**	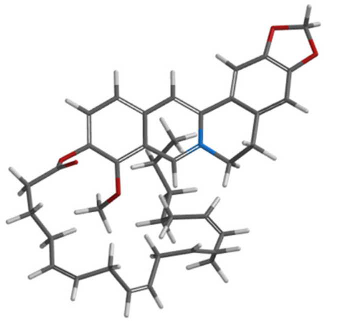<br>1.87	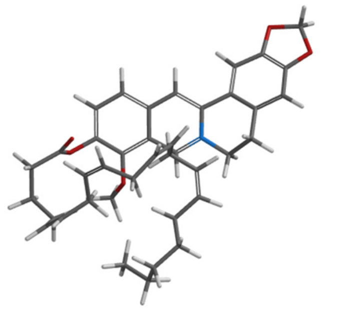<br>1.88	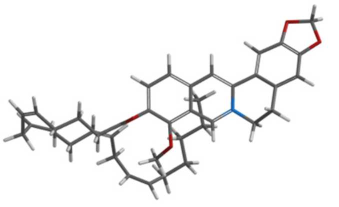<br>1.89

**2r**	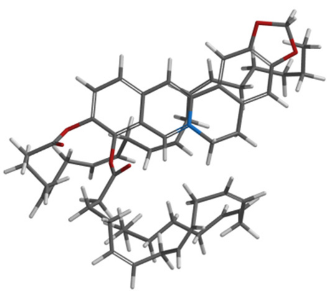 2.14	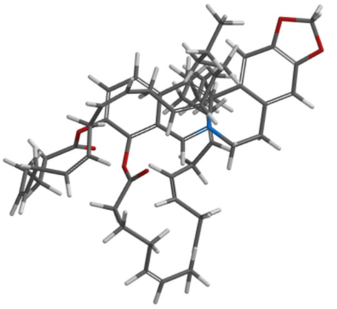 2.16	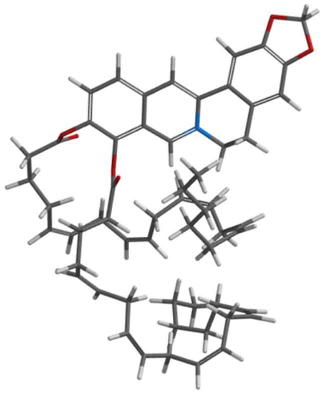 2.18
**2s**	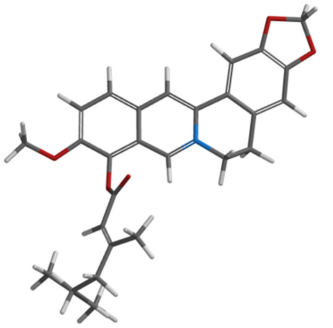 1.68	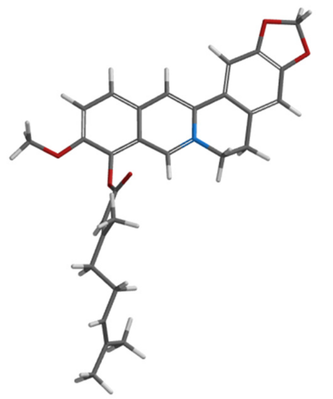 1.69	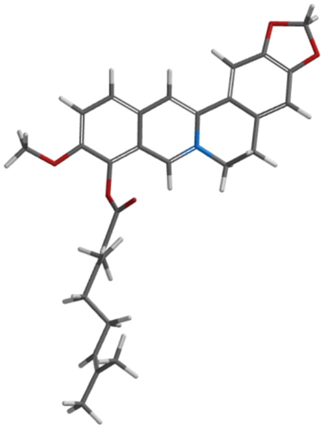 1.69
**2t**	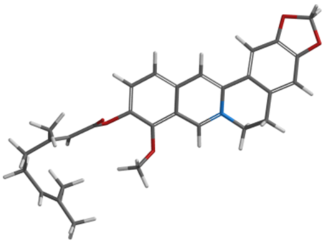 1.68	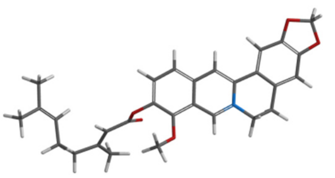 1.69	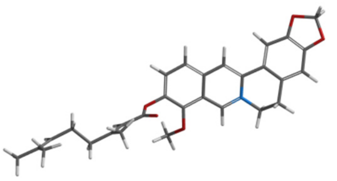 1.69
**2u**	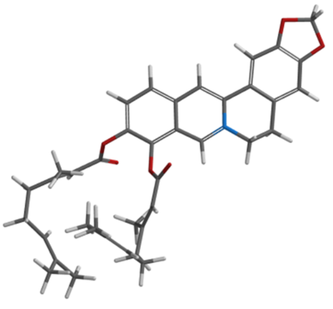 1.84	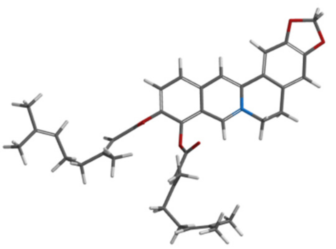 1.85	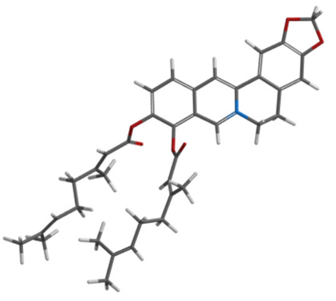 1.87

**2v**	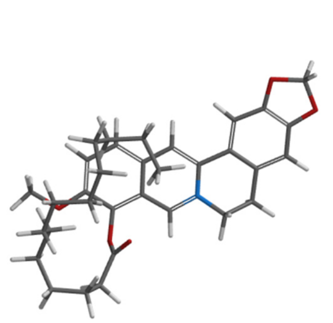<br>1.77	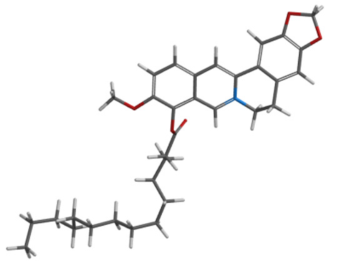<br>1.79	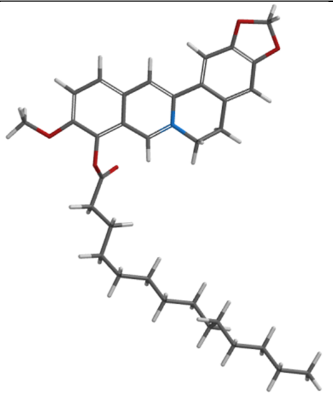<br>1.80
**2w**	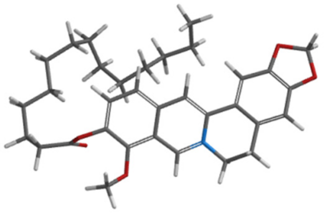<br>1.77	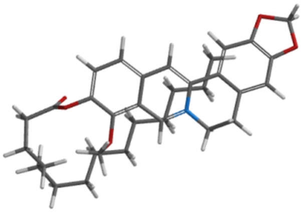<br>1.80	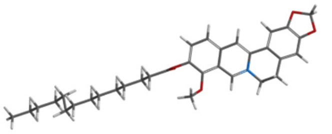<br>1.80
**2x**	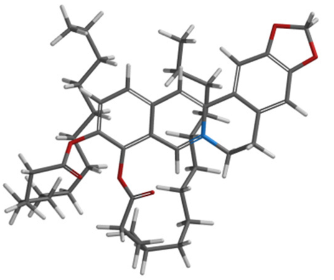<br>2.01	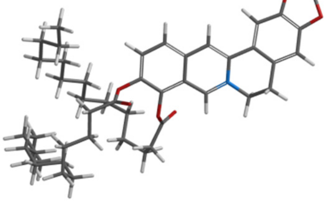<br>2.03	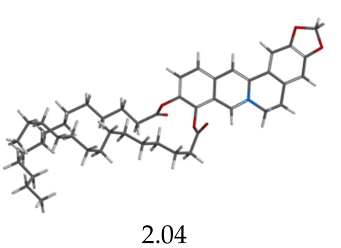<br>2.04

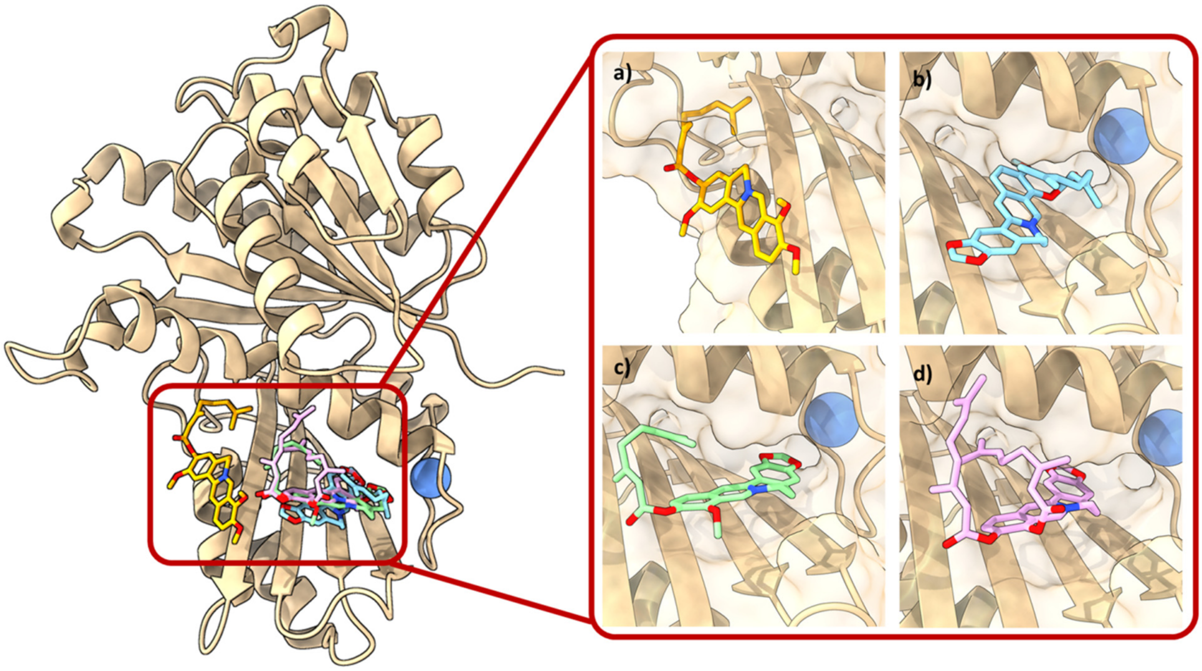

Figure S7. (*left*) Sideview of the FTsZ protein (**3voa**) with the predicted binding modes of selected berberine-geranic acid derivatives. (*right*) Expanded regions of the docked compounds (a) **1t**, (b) **2s**, (c) **2t**, and (d) **2u** into the hydrophobic site of FTsZ. Calcium ion is depicted as a light-blue solid sphere.

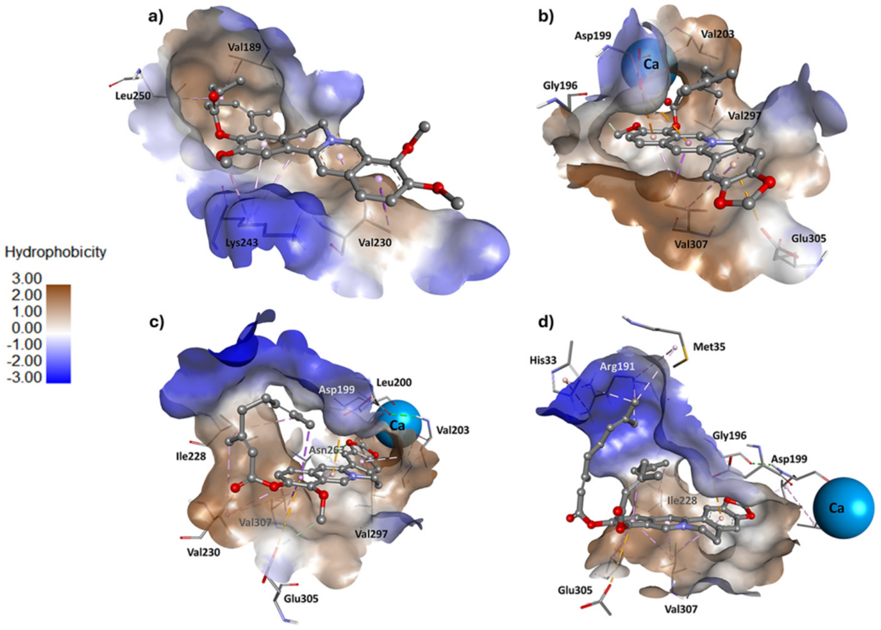

Figure S8. Hydrophobic surface for the obtained poses for molecules (a) **1t**, (b) **2s**, (c) **2t**, and (d) **2u**, showing the non-covalent interactions with the residues in FtsZ (PDB 3VOA).Calcium ion is depicted as a light-blue solid sphere.

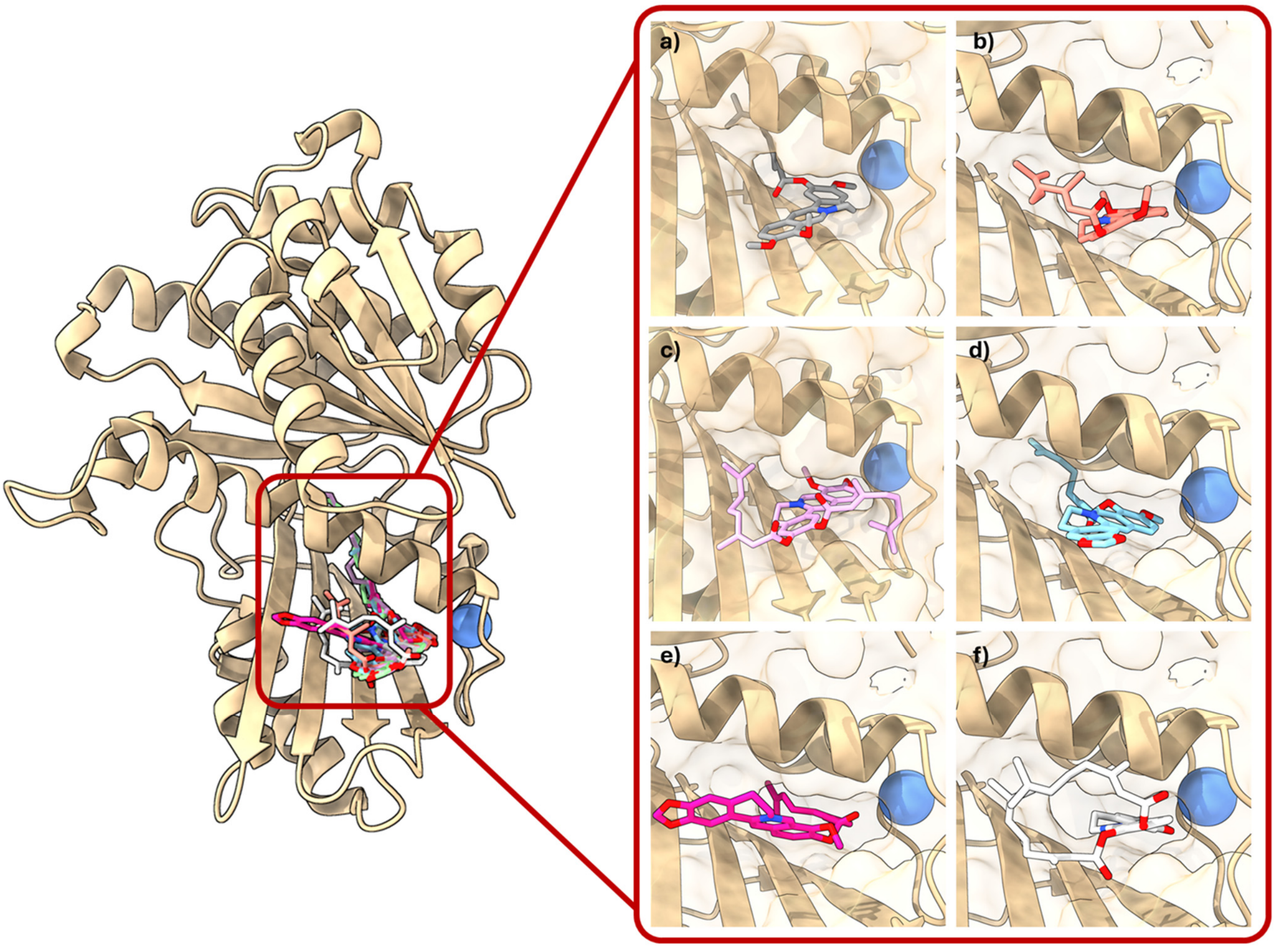

Figure S9. (*left*) Sideview of the FTsZ protein (**6kvq**) with the predicted binding modes of selected berberine-geranic acid derivatives. (*right*) Expanded regions of the docked compounds (a) **1s**, (b) **1t**, (c) **1u,** (d) **2s**, (e) **2t**, and (f) **2u** into the hydrophobic site of FTsZ. Calcium ion is depicted as a light-blue solid sphere.

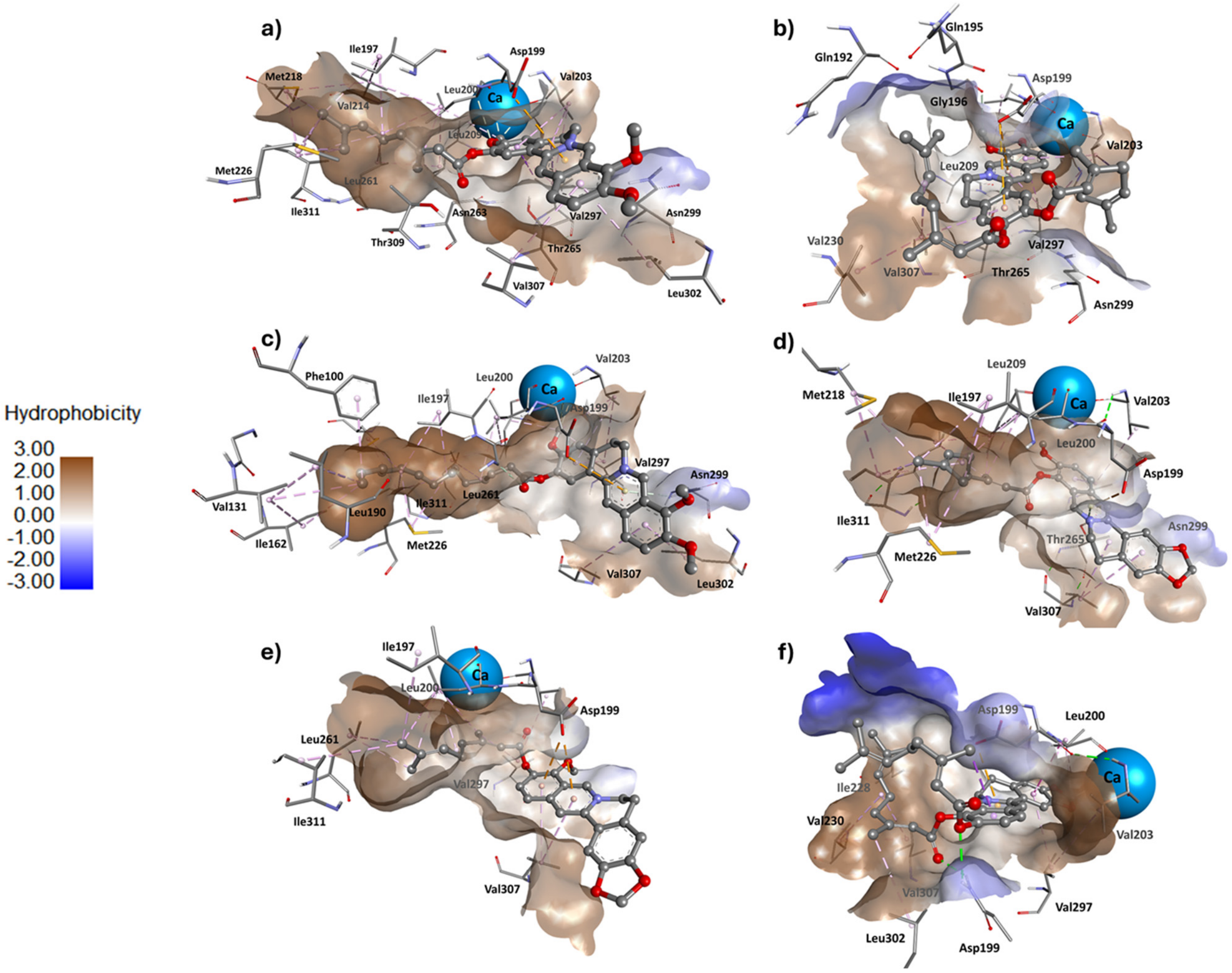

Figure S10. Hydrophobic surface for the obtained poses for molecules (a) **1t**, (b) **1s**, (c) **1u**, (d) **2s**, (e) **2t**, and (f) **2u**, showing the non-covalent interactions with the residues in FtsZ (**6kvq**).

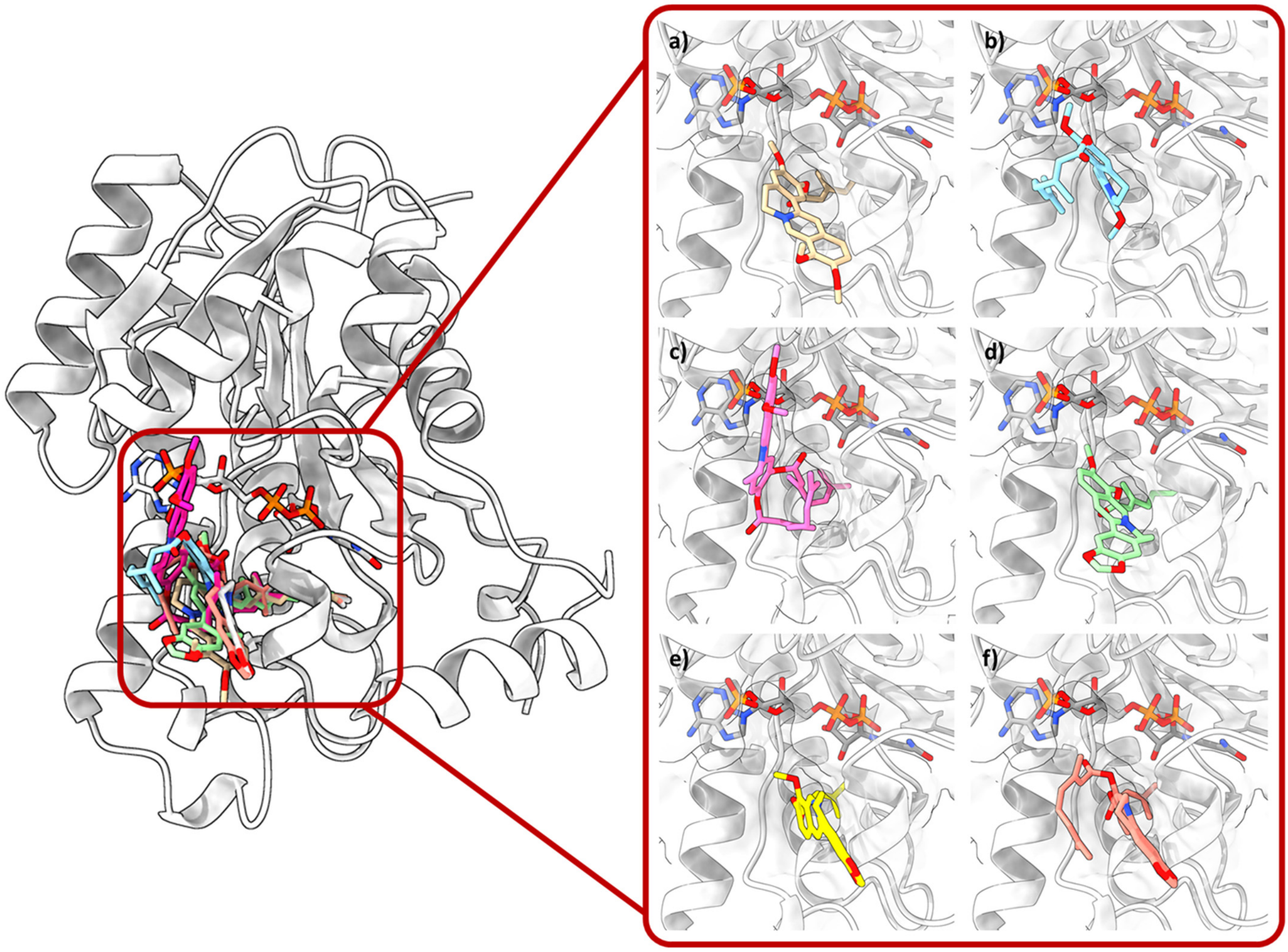

Figure S11. (left) Sideview of the FabI protein (**4alk**) with the predicted binding modes of selected berberine-fatty acid derivatives. (right) Expanded regions of the docked compounds (a) **1s**, (b) **1t**, (c) **1u**, (d) **2s**, (e) **2t**, and (f) **2u** into the hydrophobic site of FabI. It is observed that for all molecules, the alkyl fragment is inside the hydrophobic cleft, while the berberine is oriented towards the solvent.

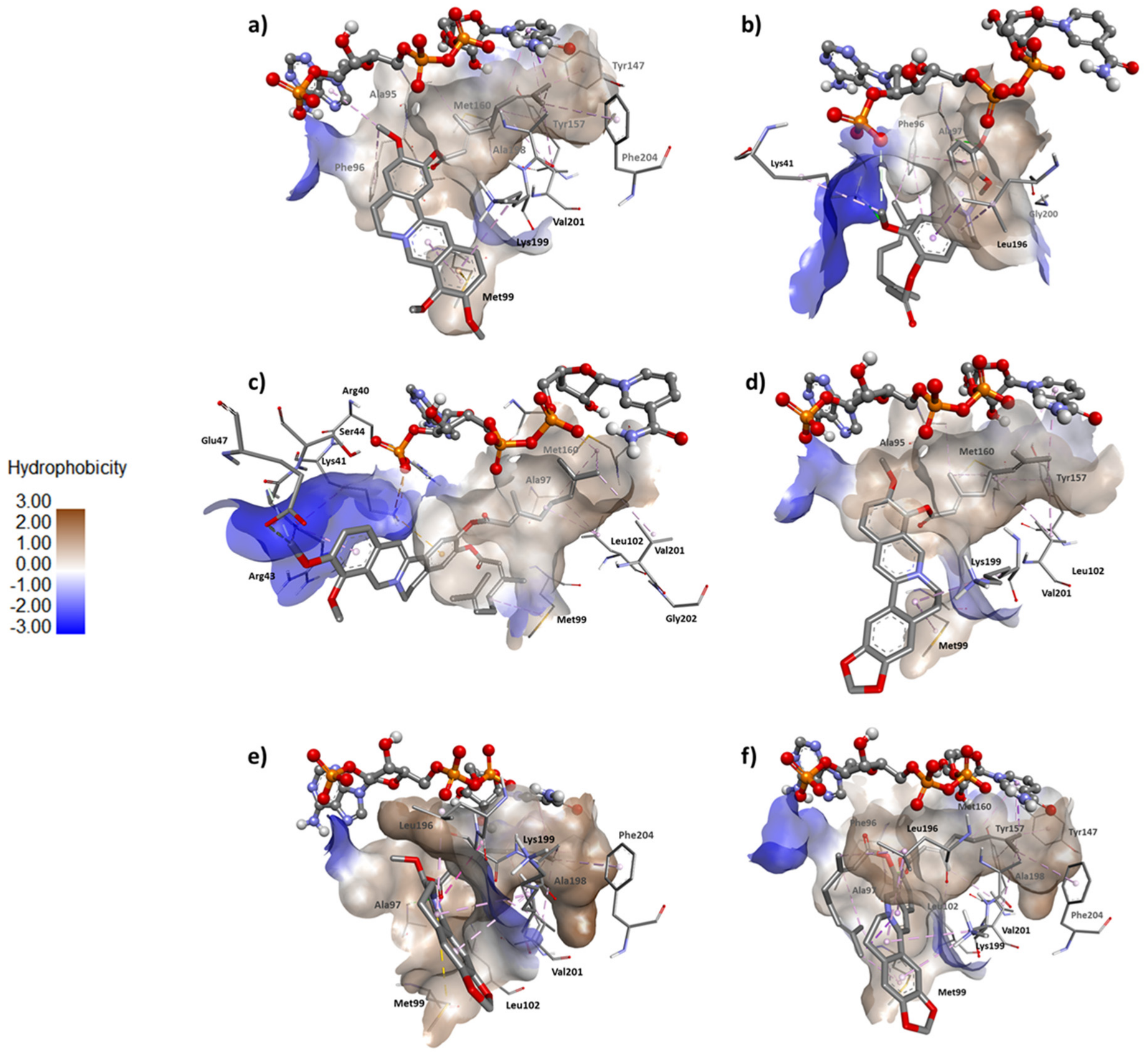

Figure S12. Hydrophobic surface for the obtained poses for molecules (a) 1s, (b) 1t, (c) 1u, (d) 2s, (e) 2t, and (f) 2u, showing the non-covalent interactions with the residues in FabI (4alk).

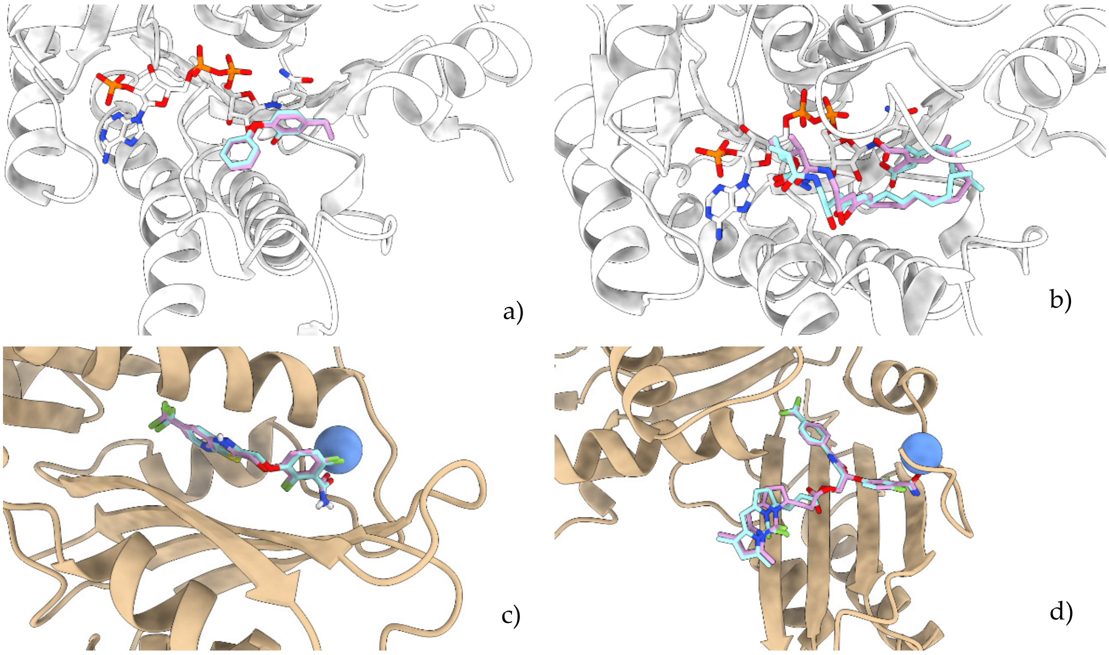

Figure S13. Docking validation of all sistems with ligand, in blue shows the co-cryztalised ligand and in pink the best pose obtainbed with Autodoc Vina. Brackets a and b (white) sows validation of FabI, in white displays NADPH co-cryztalised a) 4alk with RMSD = 0.122 and b) 6tbb with RMSD = 0.8093; c and d shows validation of Ftsz (brown) , calcium ion shows in sphere representation in color blue, c) 5xdt with RMSD = 0.2056 and d) 6kvq with RMSD = 0.4593.

Table S2. Structural and Physicochemical Characteristics of the Allosteric Binding Subpockets of FtsZ (**3voa**).

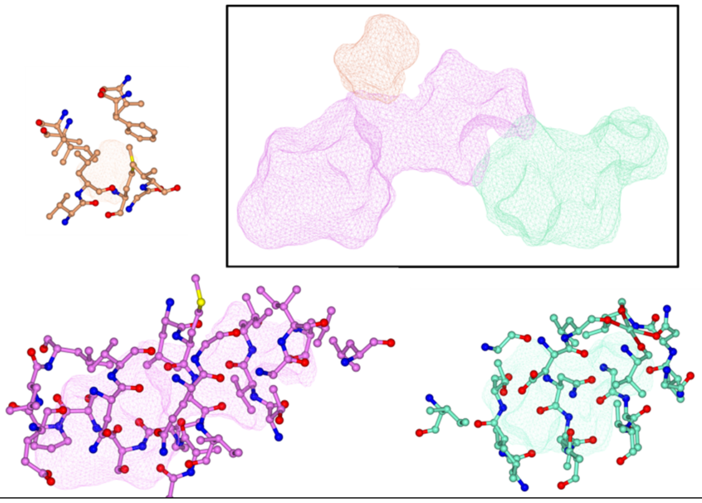

**PDB: 3voa**	Volume [Å^3]	Surface [Å^2]	Depth [Å]	Hydrophobicity ratio	aPolar ratio	Polar ratio	Drug score
**Subpocket 1 (purple)**	450.3	531.86	17.97	0.23	0.58	0.33	0.44
**Subpocket 2 (green)**	265.6	407.41	13.66	0.35	0.47	0.42	0.38
**Subpocket 3 (orange)**	41.09	76.89	0.8	0	0.89	0.11	0.38

Table S3. Structural and Physicochemical Characteristics of the Allosteric Binding Subpockets of FtsZ (**6kvq**).

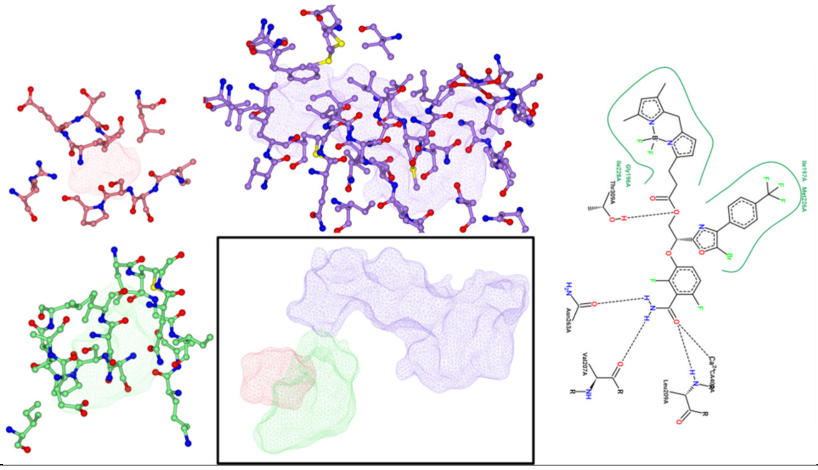

**PDB: 6kvq**	Volume [Å^3]	Surface [Å^2]	Depth [Å]	Hydrophobicity ratio	aPolar ratio	Polar ratio	Drug score
**Subpocket 1 (purple)**	640.83	660.54	22.03	0.37	0.61	0.34	0.76
**Subpocket 2 (green)**	296	384.46	12.05	0.42	0.56	0.33	0.36
**Subpocket 3 (orange)**	82.18	211.4	7.1	0.28	0.4	0.4	0.2

Table S4. Structural and physicochemical characteristics of the binding subpockets of the FabI catalytic site.

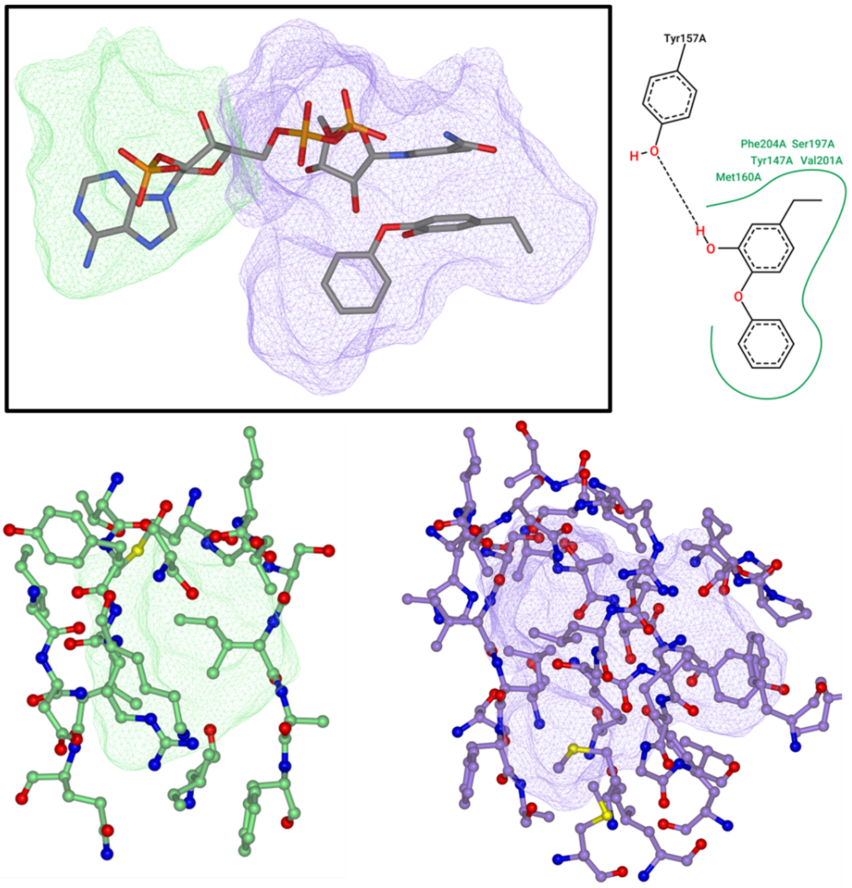

PDB: 4alk	Volume [Å^3]	Surface [Å^2]	Depth [Å]	Hydrophobicity ratio	aPolar ratio	Polar ratio	Drug score
**Subpocket 1 (purple)**	916.67	877.19	17.48	0.44	0.55	0.34	0.84
**Subpocket 2 (green)**	337.34	415.59	12.12	0.3	0.5	0.33	0.42

Table S5. Structural and Physicochemical Characteristics of the binding subpockets of the FabI catalytic site.

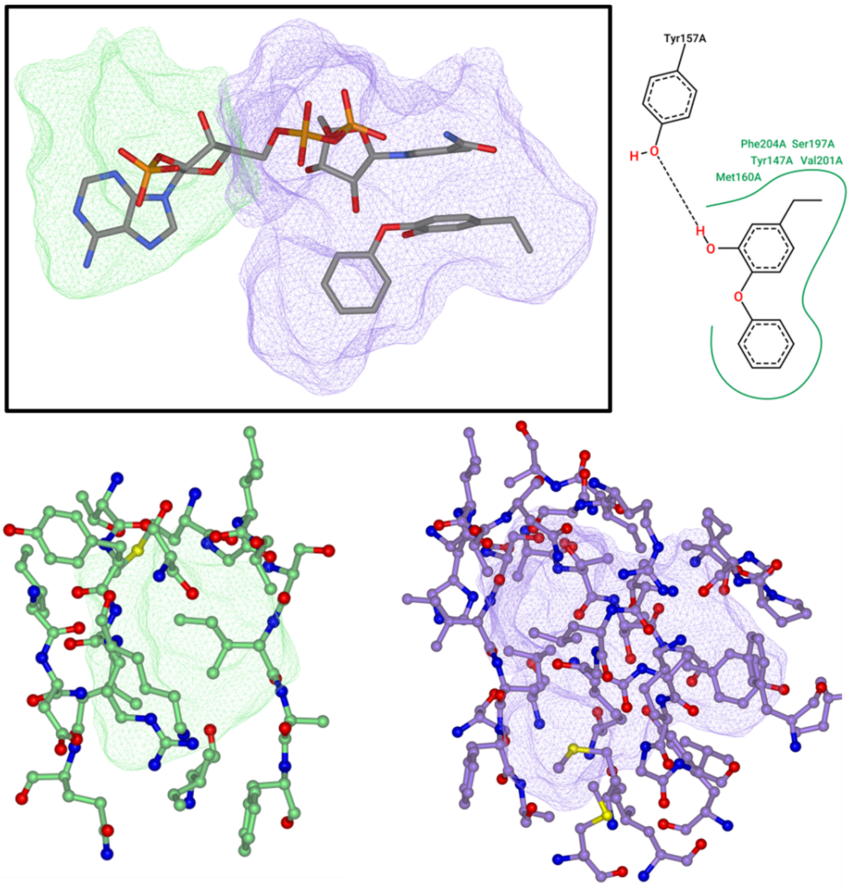

**PDB: 4alk**	Volume [Å^3]	Surface [Å^2]	Depth [Å]	Hydrophobicity ratio	aPolar ratio	Polar ratio	Drug score
**Subpocket 1 (purple)**	916.67	877.19	17.48	0.44	0.55	0.34	0.84
**Subpocket 2 (green)**	337.34	415.59	12.12	0.3	0.5	0.33	0.42

Table S6. Physicochemical Characteristics of previously reported FtsZ and FabI inhibitors and berberine derivatives

Compound	MW	# Rotable bonds	TPSA	XLogp3
**FABIA***	253.4	19	57.87	10.86
**FABIB***	281.45	19	57.87	10.86
**FABIC***	279.44	34	74.94	17.96
**FABID***	277.42	21	57.87	11.94
**FABIE***	303.46	21	57.87	11.94
**FABIF***	296.49	38	74.94	20.13
**FABIG***	294.47	18	57.87	9.92
**FABIH***	214.26	18	57.87	9.92
**FABII***	547.7	32	74.94	17.96
**Bbr**	336.36	2	40.8	3.62
**DMB**	324.35	2	62.8	3.09
**Sanguinarine**	332.33	0	40.8	4.45
**FtsZB****	504.98	8	50.03	6.63
**1a**	576.79	19	57.87	10.86
**1b**	576.79	19	57.87	10.86
**1c**	801.17	34	74.94	17.96
**1d**	604.84	21	57.87	11.94
**1e**	604.84	21	57.87	11.94
**1f**	857.27	38	74.94	20.13
**1g**	574.77	18	57.87	9.92
**1h**	574.77	18	57.87	9.92
**1i**	797.14	32	74.94	17.96
**1j**	602.82	20	57.87	11.01
**1k**	602.82	20	57.87	11.01
**1l**	853.24	36	74.94	15.8
**1m**	600.81	19	57.87	6.83
**1n**	600.81	19	57.87	6.83
**1o**	849.21	34	74.94	9.92
**1p**	624.83	19	57.87	3.09
**1q**	624.83	19	57.87	11.94
**1r**	897.25	34	74.94	9.92
**1s**	488.59	9	57.87	6.83
**1t**	488.59	9	57.87	6.83
**1u**	624.79	14	74.94	9.92
**1v**	548.73	17	57.87	9.77
**1w**	548.73	17	57.87	9.77
**1x**	745.06	30	74.94	15.8
**2a**	560.74	17	57.87	10.73
**2b**	560.74	17	57.87	10.73

**2c**	785.13	32	74.94	17.83
**2d**	588.8	19	57.87	11.81
**2e**	588.8	19	57.87	11.81
**2f**	841.23	36	74.94	20
**2g**	558.73	16	57.87	9.79
**2h**	558.73	16	57.87	9.79
**2i**	781.09	30	74.94	17.83
**2j**	586.78	18	57.87	10.88
**2k**	586.78	18	57.87	10.88
**2l**	837.2	34	74.94	15.67
**2m**	584.76	17	57.87	6.7
**2n**	584.76	17	57.87	6.7
**2o**	833.17	32	74.94	9.79
**2p**	608.79	17	57.87	11.94

FabiA-I*: Reported FabI inhibitors from Figure 2.
FtsZB**: Compound B from Figure 1.

Table S7. ADME properties of previously reported FtsZ and FabI inhibitors and berberine derivatives part 1

Compound	ML	Lipinski # violations	GI absorption	BBB permeability	Pgp substrate	Synthetic Accessibility
**FABIA***	78.38	0	High	Yes	Yes	2.84
**FABIB***	88	1	High	Yes	Yes	3.07
**FABIC***	87.52	1	High	Yes	Yes	3.1
**FABID***	87.05	1	High	No	Yes	3.03
**FABIE***	96.19	1	Low	No	Yes	3.26
**FABIF***	94.26	1	Low	No	Yes	3.16
**FABIG***	93.78	1	Low	No	Yes	3.18
**FABIH***	64.75	0	Low	No	Yes	1.68
**FABII***	153	1	Low	No	Yes	6.03
**Bbr**	94.87	0	Low	No	Yes	2.92
**DMB**	92.86	0	Low	No	Yes	2.59
**Sanguinarine**	94.68	0	Low	No	Yes	2.60
**FtsZB****	140.32	1	Low	No	Yes	3.78
**1a**	174.1	2	Low	No	Yes	5.17
**1b**	174.1	2	Low	No	Yes	5.17
**1c**	246.41	2	Low	No	Yes	7.28
**1d**	183.71	2	High	No	Yes	7.42
**1e**	183.71	2	High	No	Yes	4.92
**1f**	265.63	2	Low	No	Yes	6.74
**1g**	173.63	2	High	Yes	Yes	4.96
**1h**	173.63	2	Low	No	Yes	4.96
**1i**	245.46	2	Low	No	Yes	6.88
**1j**	183.24	2	High	No	Yes	4.71
**1k**	183.24	2	High	No	Yes	4.71
**1l**	264.69	2	Low	No	Yes	6.34
**1m**	182.77	0	Low	No	Yes	4.19
**1n**	182.77	0	Low	No	Yes	4.19
**1o**	263.74	2	Low	No	Yes	5.09
**1p**	191.43	0	Low	No	Yes	3.91
**1q**	191.43	2	Low	No	Yes	5.21
**1r**	281.07	2	Low	No	Yes	4.92
**1s**	144.31	0	Low	No	Yes	5.22
**1t**	144.31	0	Low	No	Yes	5.22
**1u**	186.83	2	Low	No	Yes	7.33
**1v**	164.49	2	Low	No	Yes	5.12
**1w**	164.49	2	Low	No	Yes	5.12

**1x**	227.18	2	Low	No	Yes	7.13
**2a**	167.18	2	Low	No	Yes	5.05
**2b**	167.18	2	Low	No	Yes	5.05
**2c**	239.48	2	Low	No	Yes	7.14
**2d**	176.79	2	High	No	Yes	7.29
**2e**	176.79	2	High	No	Yes	4.8
**2f**	258.71	2	Low	No	Yes	6.61
**2g**	166.71	2	Low	No	Yes	4.85
**2h**	166.71	2	Low	No	Yes	4.85
**2i**	238.54	2	Low	No	Yes	6.75
**2j**	176.32	2	High	No	Yes	4.6
**2k**	176.32	2	High	No	Yes	4.6
**2l**	257.76	2	Low	No	Yes	6.22
**2m**	175.85	0	Low	No	Yes	4.06
**2n**	175.85	0	Low	No	Yes	4.06
**2o**	256.82	2	Low	No	Yes	5.06
**2p**	184.51	2	Low	No	Yes	5.21
**2q**	184.51	2	High	Yes	Yes	5.1
**2r**	274.15	2	High	Yes	Yes	4.8
**2s**	137.39	0	High	Yes	Yes	5.09
**2t**	137.39	0	High	No	Yes	5.09
**2u**	179.9	2	Low	No	Yes	7.19
**2v**	157.57	2	Low	No	Yes	4.99
**2w**	157.57	2	Low	No	Yes	4.99
2x	220.26	Low	No		Yes	6.99

FabiA-I*: Reported FabI inhibitors from Figure 2.
FtsZB**: Compound B from Figure 1.

Table S8. ADME properties of previously reported FtsZ and FabI inhibitors and berberine derivatives part 2

Compound	CYP1A2 inhibitor	CYP2C19 inhibitor	CYP2C9 inhibitor	CYP2D6 inhibitor	CYP3A4 inhibitor
**FABIA**	Yes	No	No	No	No
**FABIB**	Yes	No	No	No	No
**FABIC**	Yes	No	No	No	No
**FABID**	Yes	No	No	No	No
**FABIE**	Yes	No	No	No	No
**FABIF**	Yes	No	No	No	No
**FABIG**	Yes	No	No	No	No
**FABIH**	Yes	Yes	Yes	Yes	No
**FABII**	No	Yes	Yes	No	No
**Bbr**	Yes	No	No	Yes	Yes
**DMB**	Yes	No	No	Yes	Yes
**Sanguinarine**	Yes	Yes	No	No	No
**FtsZB**	No	Yes	Yes	Yes	Yes
**1a**	No	No	No	No	No
**1b**	No	No	No	No	No
**1c**	No	No	No	No	No
**1d**	No	No	No	No	No
**1e**	No	No	No	No	No
**1f**	No	No	No	No	No
**1g**	No	No	No	Yes	No
**1h**	No	No	No	Yes	No
**1i**	No	No	No	No	No
**1j**	No	No	No	No	No
**1k**	No	No	No	No	No
**1l**	No	No	No	No	No
**1m**	No	No	Yes	No	No
**1n**	No	No	Yes	No	No
**1o**	No	No	No	Yes	No
**1p**	Yes	No	No	Yes	Yes
**1q**	No	No	No	No	No
**1r**	No	No	No	Yes	No
**1s**	No	No	Yes	No	No
**1t**	No	No	Yes	No	No
**1u**	No	No	No	Yes	No
**1v**	No	No	No	Yes	No
**1w**	No	No	No	Yes	No

**1x**	No	No	No	No	No
**2a**	No	No	No	Yes	No
**2b**	No	No	No	Yes	No
**2c**	No	No	No	No	No
**2d**	No	No	No	No	No
**2e**	No	No	No	No	No
**2f**	No	No	No	No	No
**2g**	No	No	No	Yes	No
**2h**	No	No	No	Yes	No
**2i**	No	No	No	No	No
**2j**	No	No	No	No	No
**2k**	No	No	No	No	No
**2l**	No	No	No	No	No
**2m**	No	Yes	Yes	No	No
**2n**	No	Yes	Yes	No	No
**2o**	No	No	No	No	No
**2p**	No	No	No	No	No
**2q**	No	No	No	No	No
**2r**	No	No	No	Yes	No
**2s**	No	Yes	Yes	No	No
**2t**	No	Yes	Yes	No	No
**2u**	No	No	No	No	No
**2v**	No	No	No	Yes	Yes
**2w**	No	No	No	Yes	Yes
**2x**	No	No	No	No	No

FabiA-I*: Reported FabI inhibitors from Figure 2.
FtsZB**: Compound B from Figure 1.

Table S9. *In silico* toxicological analysis for berberine, berberine derivatives, fatty acids and designed berberine-fatty acid hybrids.

SMILES	Name	AMES toxicity	Max. tolerated dose (human)	hERG I inhibitor	hERG II inhibitor	Oral Rat Acute Toxicity (LD50)	Oral Rat Chronic Toxicity (LOAEL)	Hepato-toxicity	Skin Sensitisation	*T.Pyrifor-mis* toxicity	Minnow toxicity
COc1ccc2cc3[n+](cc2c1OC)CCc1cc(O)c(O)cc1-3	Berberine	No	0.464	No	Yes	2.807	1.604	No	No	0.349	-0.646
C[n+]1cc2c3c(ccc2c2ccc4cc5c(cc4c21)OCO5)OCO3	Sanguinarine	Yes	0.413	No	Yes	2.43	1.685	No	No	0.308	-0.018
COc1c(OCCCCOc2ccc(Cl)cc2)ccc2cc3[n+](cc12)CCc1cc2c(cc1-3)OCO2	A	No	0.567	No	Yes	3.013	0.759	No	No	0.285	-5.581
CCCCC/C=C\C/C=C\C/C=C\C/C=C\CCCC(=O)Oc1cc2c(cc1OC)CC[n+]1cc3c(OC)c(OC)ccc3cc1-2	2-(((5Z,8Z,11Z,14Z)-icosa-5,8,11,14-tetraenoyl)oxy)-3,9,10-trimethoxy-5,6-dihydroisoquinolino[3,2-a]isoquinolin-7-ium	No	0.295	No	Yes	2.769	1.875	No	No	0.285	-5.168
COc1cc2c(cc1OC(=O)C=C(C)CCC=C(C)C)-c1cc3ccc(OC)c(OC)c3c[n+]1CC2	R1-Arachidonic Acid	No	0.281	No	Yes	2.82	1.648	No	No	0.285	-3.439
CCCCC/C=C\C/C=C\CCCCCCCC(=O)Oc1cc2c(cc1OC)CC[n+]1cc3c(OC)c(OC)ccc3cc1-2	R1-Geranic acid	No	0.297	No	Yes	2.791	1.822	No	No	0.285	-4.941
CCCCCCCCCCCCCC(=O)Oc1cc2c(cc1OC)CC[n+]1cc3c(OC)c(OC)ccc3cc1-2	R1-Linoleic Acid	No	0.344	No	Yes	2.896	1.766	No	No	0.286	-4.232

CCCCCCCC/C=C\CCCCCCCC(=O)Oc1cc2c(cc1OC)CC[n+]1cc3c(OC)c(OC)ccc3cc1-2	R1-Myristic Acid	No	0.302	No	Yes	2.744	1.798	No	No	0.285	-5.068
CCCCCCCCCCCCCCCC(=O)Oc1cc2c(cc1OC)CC[n+]1cc3c(OC)c(OC)ccc3cc1-2	R1-Oleic Acid	No	0.32	No	Yes	2.801	1.769	No	No	0.285	-4.713
CCCCCC/C=C\CCCCCCCC(=O)Oc1cc2c(cc1OC)CC[n+]1cc3c(OC)c(OC)ccc3cc1-2	R1-Palmitic acid	No	0.312	No	Yes	2.849	1.793	No	No	0.285	-4.586
CCCCC/C=C\C/C=C\C/C=C\C/C=C\CCCC(=O)Oc1cc2c(cc1OC(=O)CCC/C=C\C/C=C\C/C=C\C/C=C\CCCCC)-c1cc3ccc(OC)c(OC)c3c[n+]1CC2	R1-Palmitoleic Acid	No	0.469	No	Yes	2.376	1.587	No	No	0.285	-11.865
COc1ccc2cc3[n+](cc2c1OC)CCc1cc(OC(=O)C=C(C)CCC=C(C)C)c(OC(=O)C=C(C)CCC=C(C)C)cc1-3	R1, R2-Arachidonic Acid	No	0.581	No	Yes	2.413	1.272	No	No	0.285	-6.401
CCCCC/C=C\C/C=C\CCCCCCCC(=O)Oc1cc2c(cc1OC(=O)CCCCCCC/C=C\C/C=C\CCCCC)-c1cc3ccc(OC)c(OC)c3c[n+]1CC2	R1, R2-Geranic acid	No	0.468	No	Yes	2.348	1.475	No	No	0.285	-11.41
CCCCCCCCCCCCCC(=O)Oc1cc2c(cc1OC(=O)CCCCCCCCCCCCC)-c1cc3ccc(OC)c(OC)c3c[n+]1CC2	R1, R2-Linoleic Acid	No	0.365	No	Yes	2.317	1.402	No	No	0.285	-9.886
CCCCCCCC/C=C\CCCCCCCC(=O)Oc1cc2c(cc1OC(=O)CCCCCCC/C=C\CCCCCCCC	R1, R2-Myristic Acid	No	0.466	No	Yes	2.347	1.427	No	No	0.285	-11.665

)-c1cc3ccc(OC)c(OC)c3c[n+]1CC2											
CCCCCCCCCCCCCCCC(=O)Oc1cc2c(cc1OC(=O)CCCCCCCCCCCCCCC)-c1cc3ccc(OC)c(OC)c3c[n+]1CC2	R1, R2-Oleic Acid	No	0.439	No	Yes	2.321	1.386	No	No	0.285	-10.956
CCCCCC/C=C\CCCCCCCC(=O)Oc1cc2c(cc1OC(=O)CCCCCCC/C=C\CCCCCC)-c1cc3ccc(OC)c(OC)c3c[n+]1CC2	R1, R2-Palmitic acid	No	0.439	No	Yes	2.327	1.434	No	No	0.285	-10.701
CCCCCCCCCCCCCCCCCC(=O)Oc1cc2c(cc1OC(=O)CCCCCCCCCCCCCCCCC)-c1cc3ccc(OC)c(OC)c3c[n+]1CC2	R1, R2-Palmitoleic Acid	No	0.464	No	Yes	2.346	1.379	No	No	0.285	-11.919
CCCCCCCCCCCCCCCCCC(=O)Oc1cc2c(cc1OC)CC[n+]1cc3c(OC)c(OC)ccc3cc1-2	R1, R2-Stearic Acid	No	0.307	No	Yes	2.698	1.774	No	No	0.285	-5.195
CCCCC/C=C\C/C=C\C/C=C\C/C=C\CCCC(=O)Oc1cc2c(cc1OC)-c1cc3ccc(OC)c(OC)c3c[n+]1CC2	R1-Stearic Acid	No	0.127	No	Yes	2.685	1.919	No	No	0.285	-4.893
COc1cc2c(cc1OC(=O)C=C(C)CCC=C(C)C)CC[n+]1cc3c(OC)c(OC)ccc3cc1-2	R2-Arachidonic Acid	No	0.347	No	Yes	3.077	1.574	No	No	0.288	-3.583
CCCCC/C=C\C/C=C\CCCCCCCC(=O)Oc1cc2c(cc1OC)-c1cc3ccc(OC)c(OC)c3c[n+]1CC2	R2-Geranic acid	No	0.127	No	Yes	2.703	1.866	No	No	0.285	-4.666

CCCCCCCCCCCCCC(=O)Oc1cc2c(cc1OC)-c1cc3ccc(OC)c(OC)c3c[n+]1CC2	R2-Linoleic Acid	No	0.199	No	Yes	2.781	1.81	No	No	0.285	-3.957
CCCCCCCC/C=C\CCCCCCCC(=O)Oc1cc2c(cc1OC)-c1cc3ccc(OC)c(OC)c3c[n+]1CC2	R2-Myristic Acid	No	0.134	No	Yes	2.671	1.842	No	No	0.285	-4.793
CCCCCCCCCCCCCCCC(=O)Oc1cc2c(cc1OC)-c1cc3ccc(OC)c(OC)c3c[n+]1CC2	R2-Oleic Acid	No	0.158	No	Yes	2.713	1.813	No	No	0.285	-4.439
CCCCCC/C=C\CCCCCCCC(=O)Oc1cc2c(cc1OC)-c1cc3ccc(OC)c(OC)c3c[n+]1CC2	R2-Palmitic acid	No	0.149	No	Yes	2.746	1.837	No	No	0.285	-4.312
CCCCCCCCCCCCCCCCCC(=O)Oc1cc2c(cc1OC)-c1cc3ccc(OC)c(OC)c3c[n+]1CC2	R2-Palmitoleic Acid	No	0.14	No	Yes	2.639	1.818	No	No	0.285	-4.92
CCCCC/C=C\C/C=C\C/C=C\C/C=C\CCCC(=O)Oc1c(OC)ccc2cc3[n+](cc12)CCc1cc2c(cc1-3)OCO2	R2-Stearic Acid	No	0.212	No	Yes	2.931	1.48	No	No	0.285	-5.763
COc1ccc2cc3[n+](cc2c1OC(=O)C=C(C)CCC=C(C)C)CCc1cc2c(cc1-3)OCO2	R3-Arachidonic Acid	No	0.217	No	Yes	2.748	0.875	No	No	0.295	-3.755
CCCCC/C=C\C/C=C\CCCCCCCC(=O)Oc1c(OC)ccc2cc3[n+](cc12)CCc1cc2c(cc1-3)OCO2	R3-Geranic Acid	No	0.216	No	Yes	2.943	1.427	No	No	0.285	-5.535

CCCCCCCCCCCCCC(=O)Oc1c(OC)ccc2cc3[n+](cc12)CCc1cc2c(cc1-3)OCO2	R3-Linoleic Acid	No	0.264	No	Yes	3.017	1.369	No	No	0.286	-4.826
CCCCCCCC/C=C\CCCCCCCC(=O)Oc1c(OC)ccc2cc3[n+](cc12)CCc1cc2c(cc1-3)OCO2	R3-Myristic Acid	No	0.228	No	Yes	2.895	1.403	No	No	0.285	-5.663
CCCCCCCCCCCCCCCC(=O)Oc1c(OC)ccc2cc3[n+](cc12)CCc1cc2c(cc1-3)OCO2	R3-Oleic Acid	No	0.249	No	Yes	2.94	1.372	No	No	0.285	-5.308
CCCCCC/C=C\CCCCCCCC(=O)Oc1c(OC)ccc2cc3[n+](cc12)CCc1cc2c(cc1-3)OCO2	R3-Palmitic acid	No	0.232	No	Yes	2.988	1.396	No	No	0.285	-5.181
CCCCC/C=C\C/C=C\C/C=C\C/C=C\CCCC(=O)Oc1ccc2cc3[n+](cc2c1OC(=O)CCC/C=C\C/C=C\C/C=C\C/C=C\CCCCC)CCc1cc2c(cc1-3)OCO2	R3-Palmitoleic Acid	No	0.461	No	Yes	2.245	0.87	No	No	0.285	-12.805
CC(C)=CCC=C(C)CC(=O)Oc1c(OC(=O)C=C(C)CCC=C(C)C)ccc2cc3[n+](cc12)CCc1cc2c(cc1-3)OCO2	R3, R4-Arachidonic Acid	No	0.403	No	Yes	2.996	1.569	No	No	0.285	-6.544
CCCCC/C=C\C/C=C\CCCCCCCC(=O)Oc1ccc2cc3[n+](cc2c1OC(=O)CCCCCCC/C=C\C/C=C\CCCCC)CCc1cc2c(cc1-3)OCO2	R3, R4-Geranic Acid	No	0.459	No	Yes	2.197	0.775	No	No	0.285	-12.35
CCCCCCCCCCCCCC(=O)Oc1ccc2cc3[n+](cc2c1OC(=O)CCCCCCCCCCCCC)CCc1cc2c(cc1-3)OCO2	R3, R4-Linoleic Acid	No	0.38	No	Yes	2.185	0.704	No	No	0.285	-10.826
CCCCCCCC/C=C\CCCCCCCC(=O)Oc1ccc2cc3[n+](cc2c1	R3, R4-Myristic Acid	No	0.458	No	Yes	2.183	0.726	No	No	0.285	-12.605

OC(=O)CCCCCCC/C=C\CCCCCCCC)CCc1cc2c(cc1-3)OCO2											
CCCCCCCCCCCCCCCC(=O)Oc1ccc2cc3[n+](cc2c1OC(=O)CCCCCCCCCCCCCCC)CCc1cc2c(cc1-3)OCO2	R3, R4-Oleic Acid	No	0.435	No	Yes	2.152	0.687	No	No	0.285	-11.896
CCCCCC/C=C\CCCCCCCC(=O)Oc1ccc2cc3[n+](cc2c1OC(=O)CCCCCCC/C=C\CCCCCC)CCc1cc2c(cc1-3)OCO2	R3, R4-palmitic acid	No	0.434	No	Yes	2.175	0.735	No	No	0.285	-11.641
CCCCCCCCCCCCCCCCCC(=O)Oc1ccc2cc3[n+](cc2c1OC(=O)CCCCCCCCCCCCCCCCC)CCc1cc2c(cc1-3)OCO2	R3, R4-Palmitoleic Acid	No	0.456	No	Yes	2.173	0.678	No	No	0.285	-12.859
CCCCCCCCCCCCCCCCCC(=O)Oc1c(OC)ccc2cc3[n+](cc12)CCc1cc2c(cc1-3)OCO2	R3, R4-Stearic Acid	No	0.239	No	Yes	2.847	1.379	No	No	0.285	-5.79
CCCCC/C=C\C/C=C\C/C=C\C/C=C\CCCC(=O)Oc1ccc2cc3[n+](cc2c1OC)CCc1cc2c(cc1-3)OCO2	R3-Stearic Acid	No	0.156	No	Yes	2.9	1.228	No	No	0.285	-5.256
COc1c(OC(=O)C=C(C)CCC=C(C)C)ccc2cc3[n+](cc12)CCc1cc2c(cc1-3)OCO2	R4-Arachidonic Acid	No	0.233	No	Yes	3.092	1.364	No	No	0.289	-4.036
CCCCC/C=C\C/C=C\CCCCCCCC(=O)Oc1ccc2cc3[n+](cc2c1OC)CCc1cc2c(cc1-3)OCO2	R4-Geranic Acid	No	0.173	No	Yes	2.874	1.175	No	No	0.285	-5.028
CCCCCCCCCCCCCC(=O)Oc1ccc2cc3[n+](cc2c1OC)CCc1cc2c(cc1-3)OCO2	R4-Linoleic Acid	No	0.269	No	Yes	2.866	1.12	No	No	0.287	-4.319

CCCCCCCC/C=C\CCCCCCCC(=O)Oc1ccc2cc3[n+](cc2c1OC)CCc1cc2c(cc1-3)OCO2	R4-Myristic Acid	No	0.183	No	Yes	2.828	1.151	No	No	0.285	-5.156
CCCCCCCCCCCCCCCC(=O)Oc1ccc2cc3[n+](cc2c1OC)CCc1cc2c(cc1-3)OCO2	R4-Oleic Acid	No	0.225	No	Yes	2.834	1.122	No	No	0.286	-4.801
CCCCCC/C=C\CCCCCCCC(=O)Oc1ccc2cc3[n+](cc2c1OC)CCc1cc2c(cc1-3)OCO2	R4-Palmitic Acid	No	0.21	No	Yes	2.879	1.146	No	No	0.286	-4.674
CCCCCCCCCCCCCCCCCC(=O)Oc1ccc2cc3[n+](cc2c1OC)CCc1cc2c(cc1-3)OCO2	R4-Palmitoleic Acid	No	0.192	No	Yes	2.782	1.127	No	No	0.285	-5.283
COc1ccc2cc3[n+](cc2c1OCCCOc1ccccc1)CCc1cc2c(cc1-3)OCO2	R4-Stearic Acid	No	0.611	No	Yes	2.725	0.693	No	No	0.286	-4.895
COc1ccc2cc3[n+](cc2c1OCCCOc1ccc(Cl)cc1)CCc1cc2c(cc1-3)OCO2	B	No	0.56	No	Yes	2.756	0.517	No	No	0.286	-4.987
COc1ccc(OCCCOc2c(OC)ccc3cc4[n+](cc23)CCc2cc3c(cc2-4)OCO3)cc1	C	No	0.598	No	Yes	3.23	0.759	No	No	0.285	-4.283
COc1ccc2cc3[n+](cc2c1OCCCOc1ccccc1C)CCc1cc2c(cc1-3)OCO2	D	No	0.616	No	Yes	2.742	0.718	No	No	0.286	-4.992
COc1ccc2cc3[n+](cc2c1OCCCOc1ccc([N+](=O)[O-])cc1)CCc1cc2c(cc1-3)OCO2	E	Yes	0.341	No	Yes	2.757	0.581	No	No	0.285	-5.652
COc1ccc2cc3[n+](cc2c1OCCCCOc1ccccc1)CCc1cc2c(cc1-3)OCO2	F	No	0.592	No	Yes	2.639	0.607	No	No	0.286	-5.176
COc1ccc2cc3[n+](cc2c1OCCCBr)CCc1cc2c(cc1-3)OCO2	G	No	0.167	No	Yes	2.427	0.575	Yes	No	0.303	-1.954

CCCCCC/C=C\CCCCCCCC(=O)O	Palmitoleic acid	No	-0.713	No	No	1.449	3.109	No	Yes	0.865	-0.956
CCCCCCCC/C=C\CCCCCCCC(=O)O	Oleic acid	No	-0.81	No	No	1.417	3.259	No	Yes	0.676	-1.438
CCCCC/C=C\C/C=C/CCCCCCCC(=O)O	Linoleic acid	No	-0.827	No	No	1.429	3.187	Yes	Yes	0.701	-1.31
CC/C=C\C/C=C\C/C=C\CCCCCCCC(=O)O	Alpha linolenic acid	No	-0.84	No	No	1.441	3.115	Yes	Yes	0.722	-1.183
CCCCC/C=C\C/C=C\C/C=C\C/C=C\CCCC(=O)O	Arachidonic acid	No	-0.92	No	No	1.435	3.196	Yes	Yes	0.562	-1.538
CCCCCCCC/C=C\CCCCCCCC(=O)OC	Methyl oleate	No	0.04	No	No	1.637	3.075	No	Yes	1.529	-1.727
CCCCC/C=C\C/C=C\CCCCCCCC(=O)OC	Methyl linoleate	No	-0.019	No	No	1.617	3.004	No	Yes	1.603	-1.6
CCc1ccc(Oc2ccccc2)c(O)c1	5-ethyl-2-phenoxyphenol (E9P)	No	1.215	No	No	1.685	2.286	No	Yes	1.307	0.45
C=C(CC/C=C\C=C\CC(C)CC(=O)CC(O)CNC(=O)C(C)C(C)OC(N)=O)CC(C)C=C(C)CC(=O)O	Kalimantacin A	No	-0.478	No	No	2.214	2.061	Yes	No	0.286	0.073
CCCCC/C=C\C/C=C\CCCCCCCC(=O)O[C@H]1CC[C@@]2(C)C(=CC[C@H]3[C@@H]4CC[C@H]([C@H](C)CCCC(C)C)[C@@]4(C)CC[C@@H]32)C1	Cholesteryl linoleate	No	-0.006	No	Yes	2.379	3.435	No	No	0.285	-7.994
CCCCC/C=C\C/C=C\C/C=C\C/C=C\CCCC(=O)O[C@H]1CC[C@@]2(C)C(=CC[C@H]3[C@@H]4CC[C@H]([C@H](C)CCCC(C)C)[C@@]4(C)CC[C@@H]32)C1	Cholesteryl arachidonate	No	0.034	No	Yes	2.375	3.444	No	No	0.285	-8.221

Table S10. Predicted MIC values of most affine berberine derivatives for FtsZ and FabI

Compound	FabI MIC (mM)	FtsZ MIC (μg/mL)	FtsZ IC_{50} (mM)
**1s**	0.456	625.43	1.280
**1t**	0.478	6.35	0.013
**1u**	0.159	158.59	0.254
**2s**	0.263	5.86	0.012
**2t**	0.188	8.14	0.017
**2u**	0.146	24.73	0.041

Table S11. Docking parameters per crystal

PDB ID	COORDINATES			DIMENSIONS		
	X	Y	Z	X	Y	Z
**4alk**	-40	-34.87	40	29	36	28.5676
**6tbb**	-19.61	30.293	-74.5988	29	33.27	28.5676
**3voa**	-0.5647	13.791	25.9557	27	34	33.5
**5xdt**	-0.5647	13.791	25.9557	27	34	33.5
**6kvq**	-1.9109	10.1823	24.1717	23.5	24.65	23.3
